# Highly enantioselective metallation–substitution alpha to a chiral nitrile†
†Electronic supplementary information (ESI) available: Experimental details, spectroscopic data, ReactIR, kinetics details, X-ray and DFT data, and NMR spectra. CCDC 1477823. For ESI and crystallographic data in CIF or other electronic format see DOI: 10.1039/c6sc03712g



**DOI:** 10.1039/c6sc03712g

**Published:** 2016-10-25

**Authors:** Arghya Sadhukhan, Melanie C. Hobbs, Anthony J. H. M. Meijer, Iain Coldham

**Affiliations:** a Department of Chemistry , University of Sheffield , Brook Hill , Sheffield , S3 7HF , UK . Email: i.coldham@sheffield.ac.uk

## Abstract

We report the deprotonation of a chiral nitrile and reaction of the resulting chiral organometallic species with a variety of electrophiles to give highly enantiomerically enriched 2-substituted nitrile products. The nitrile was treated with TMPMgCl and the resulting anion, an asymmetric alpha cyano Grignard species, was found to be configurationally stable at low temperature for a short time (half-life several minutes at –104 °C).

## Abstract 2

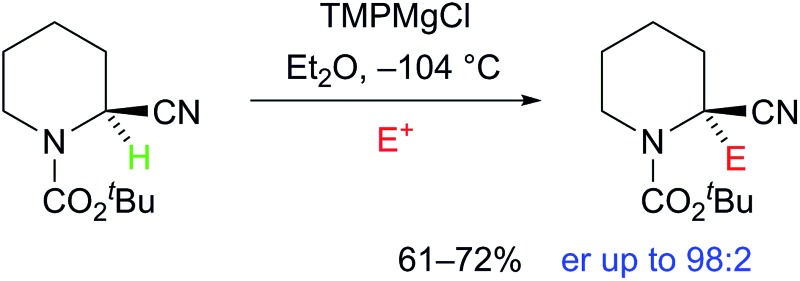
Nitrile anions do not necessarily lack stereochemical integrity and we show good results for stereospecific reaction with a simple magnesium base.

## Introduction

**Fig. 1:**
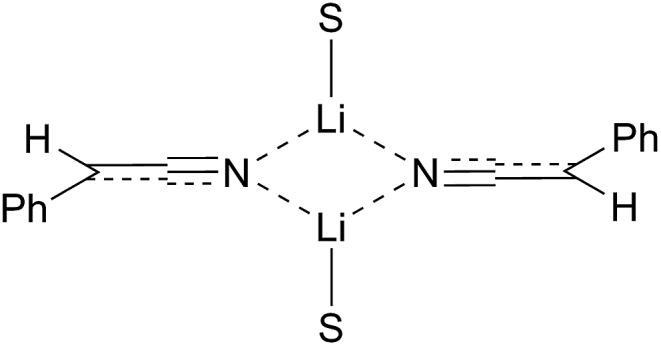
Structure of lithiatiated phenylacetonitrile, S = solvent.^3,4^

Metallated nitriles are well-used intermediates in synthetic chemistry due to their excellent reactivity as nucleophiles.^1^ The formation of the organometallic species and its reaction with an electrophile such as an alkyl halide or aldehyde allows a high-yielding preparation of the desired substituted nitrile that can then be converted readily to other functional groups. The most common method to prepare the metallated nitrile is to treat the nitrile with a base such as lithium diisopropylamide (LDA) and this is known to give the lithiated nitrile in which the lithium ion normally resides on the nitrogen atom.^2–5^ Although this gives a reactive nitrile anion, one of its drawbacks is that this provides an achiral organometallic species (*e.g.* Fig. 1),^3,4^ even starting from a chiral, enantiomerically enriched nitrile. Therefore it would be expected that achiral products would result from using chiral enantiomerically enriched nitrile starting materials and this is typically the case.^6–8^

**Fig. 2:**
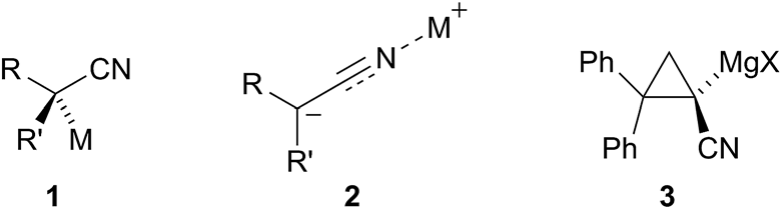
Simplified representations of metallated nitriles, M = metal.

Remarkably, however, Takeda and co-workers reported recently that it is possible in certain cases at low temperature with *in situ* reactive electrophiles to trap the intermediate anions to give enantioenriched products.^9,10^ At about the same time, we began to explore this possibility but by using magnesiated nitriles.^11^ The idea that magnesiated nitriles may allow asymmetric reaction through a chiral organometallic species **1** rather than **2** (Fig. 2) was based on results from several groups including that of Carlier and co-workers, who reported the first metallated nitrile with macroscopic configurational stability, albeit a cyclopropyl derivative **3** (prepared by Br–Mg exchange).^12^ In addition, Fleming and co-workers had found opposing selectivities for reactions of lithiated and magnesiated nitriles and surmised that the magnesium cation has a preference for location on carbon.^13,14^

If the metal atom is located on the carbon atom, as illustrated by the contact ion pair **1** (or its related solvent-separated ion pair in which the metal cation is nearby), then the metallated nitrile is chiral and has the possibility to transfer its chirality to the product on reaction with an electrophile. However, very little is known about the rate of enantiomerisation of such nitrile anions.^11,15^ The importance of nitrogen-containing heterocycles in natural products and medicinal compounds led us to explore the metallation of nitrile **4** with the aim to determine whether the deprotonation–electrophilic quench would be feasible and how fast the intermediate magnesiated nitrile undergoes racemisation. Herein, we describe the first high yielding, highly enantioselective metallation–substitutions of a chiral α-amino-nitrile by using a simple magnesium base.

## Results and discussion

The carboxylic acid *N*-Boc-pipecolic acid is commercially available as the (*S*) enantiomer and this was converted to (*S*)-*N*-Boc-2-cyanopiperidine **4** in two steps (see ESI†). This method involved simple amide formation with ethyl chloroformate and ammonia to give the primary amide, followed by dehydration to give the nitrile (*S*)-**4**. The corresponding racemic nitrile could be prepared in the same way starting from racemic pipecolic acid by initial *N*-Boc protection with Boc_2_O, Et_3_N, CH_2_Cl_2_ followed by using the same method.

Initially we investigated the deprotonation of the nitrile (*S*)-**4** with LDA in THF at –78 °C. After 10 min, the anion was quenched by addition of various electrophiles. Under these conditions we generally obtained good yields of racemic products (see ESI†), as determined by chiral stationary phase (CSP) GC or HPLC analysis. The formation of racemic products was expected under these conditions as lithiated nitriles are known to exist with the lithium cation on the nitrogen atom^2–5^ and therefore the stereochemistry at the carbon centre would be readily lost.

We then turned to the asymmetric reaction by deprotonation and electrophilic quench, particularly on using a softer metal counterion where we hoped that the metal would remain on the carbon centre for long enough to maintain the configuration. We had previously obtained some success in this approach by using magnesium bases,^11^ with one equivalent of ^i^PrMgCl, TMPMgCl·LiCl, or TMPMgCl. All these bases resulted in significant enantioenrichment after leaving the magnesiated intermediate for 5 s at –107 °C prior to addition of acetone (enantiomer ratio, er, up to 96 : 4 of the substituted product **5e**). However the conversion in these reactions was low and this product could not be isolated.

**Fig. 3:**
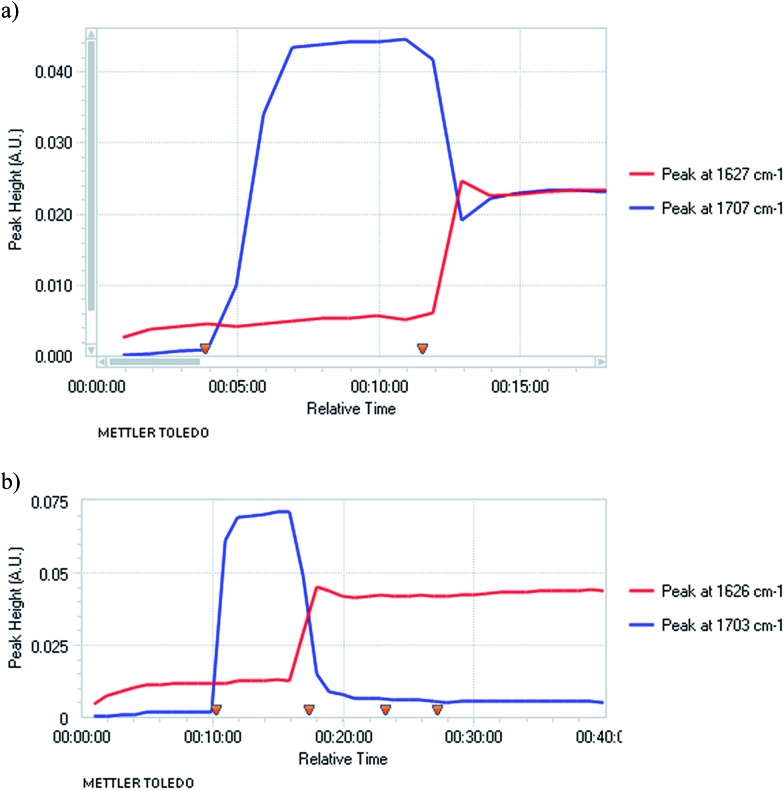
*In situ* IR spectroscopy of the deprotonation of **4** with TMPMgCl in THF/Et_2_O at –104 °C with time in h:min:sec. (a) One equiv. TMPMgCl added at time 12 min (*ν*_C=O_ **4** 1707 cm^–1^, *ν*_C=O_ metallated **4** 1627 cm^–1^); (b) three equiv. TMPMgCl added at time 16 min (*ν*_C=O_ **4** 1703 cm^–1^, *ν*_C=O_ metallated **4** 1626 cm^–1^).

To optimise the reaction we carried out *in situ* IR studies and found that only partial conversion occurs with one equivalent of TMPMgCl (Fig. 3a). However, almost complete conversion occurs with two equivalents and full conversion with three equivalents of TMPMgCl (Fig. 3b).

**Scheme 1:**
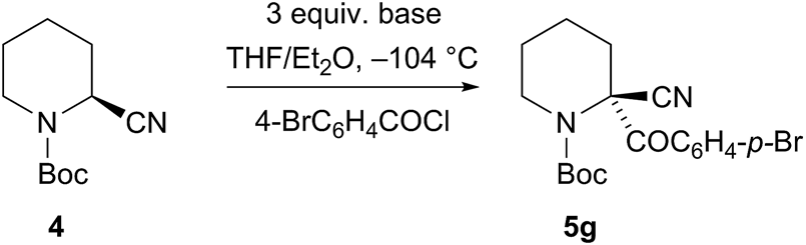
Metallation-quench of nitrile **4** with various bases.

Several bases were tested under these optimised conditions (Scheme 1). The bases CuO^*t*^Bu, mesityl copper, or TMPZnCl·MgCl_2_ were unsuccessful. The method developed by Takeda and co-workers^10^ with NaHMDS (and 4-BrC_6_H_4_COCl *in situ*) did give the product **5g** but the enantioselectivity was poor (71% yield, er 53 : 47). With the magnesium base iPrMgCl the yield was low (10% yield of product **5a** with PhSSO_2_Ph as the *in situ* electrophile, er not determined). However the base TMPMgCl was much more successful and a good yield and er of the product **5g** was obtained (68% yield **5g**, er 81 : 19).

**Fig. 4:**
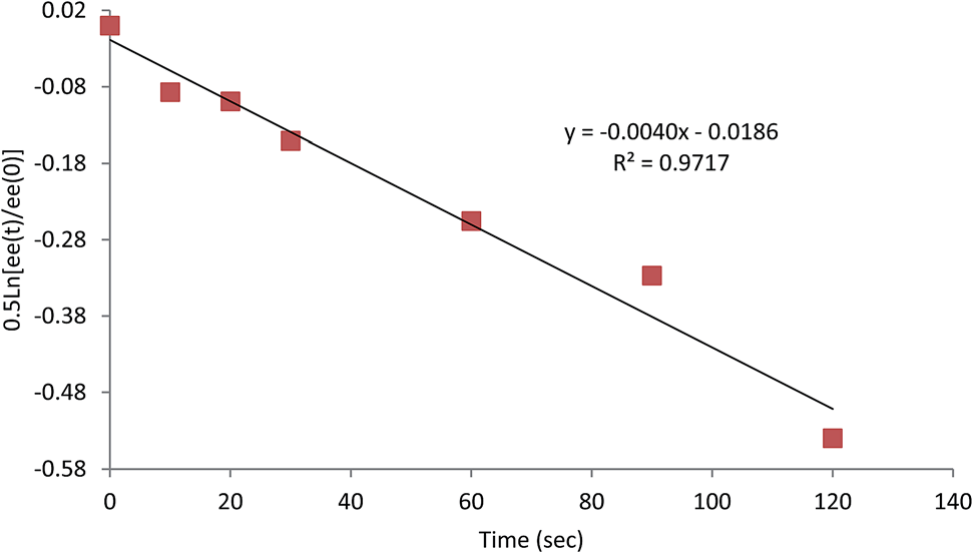
log plot of loss of ee against time of **4** + TMPMgCl in Et_2_O at –104 °C.

We therefore selected TMPMgCl as the most suitable base. The enantioselectivity was not optimal and we were aware that Carlier and co-workers had found that a magnesiated cyclopropylnitrile racemises more rapidly in THF than in Et_2_O.^15^ Therefore we conducted kinetic experiments to determine the rate of enantiomerisation of this organomagnesium species in THF/Et_2_O (1 : 1) and in Et_2_O (see ESI† and Fig. 4). At –104 °C the intermediate organomagnesium compound was trapped after various time periods to give the product **5a** or **5f** and the er was measured by CSP HPLC or GC respectively. These gave good first order plots and revealed rates for inversion *k* ∼ 6.5 × 10^–3^ s^–1^ in THF/Et_2_O (see ESI†) and *k* ∼ 4 × 10^–3^ s^–1^ in Et_2_O (Fig. 4).

**Scheme 2:**
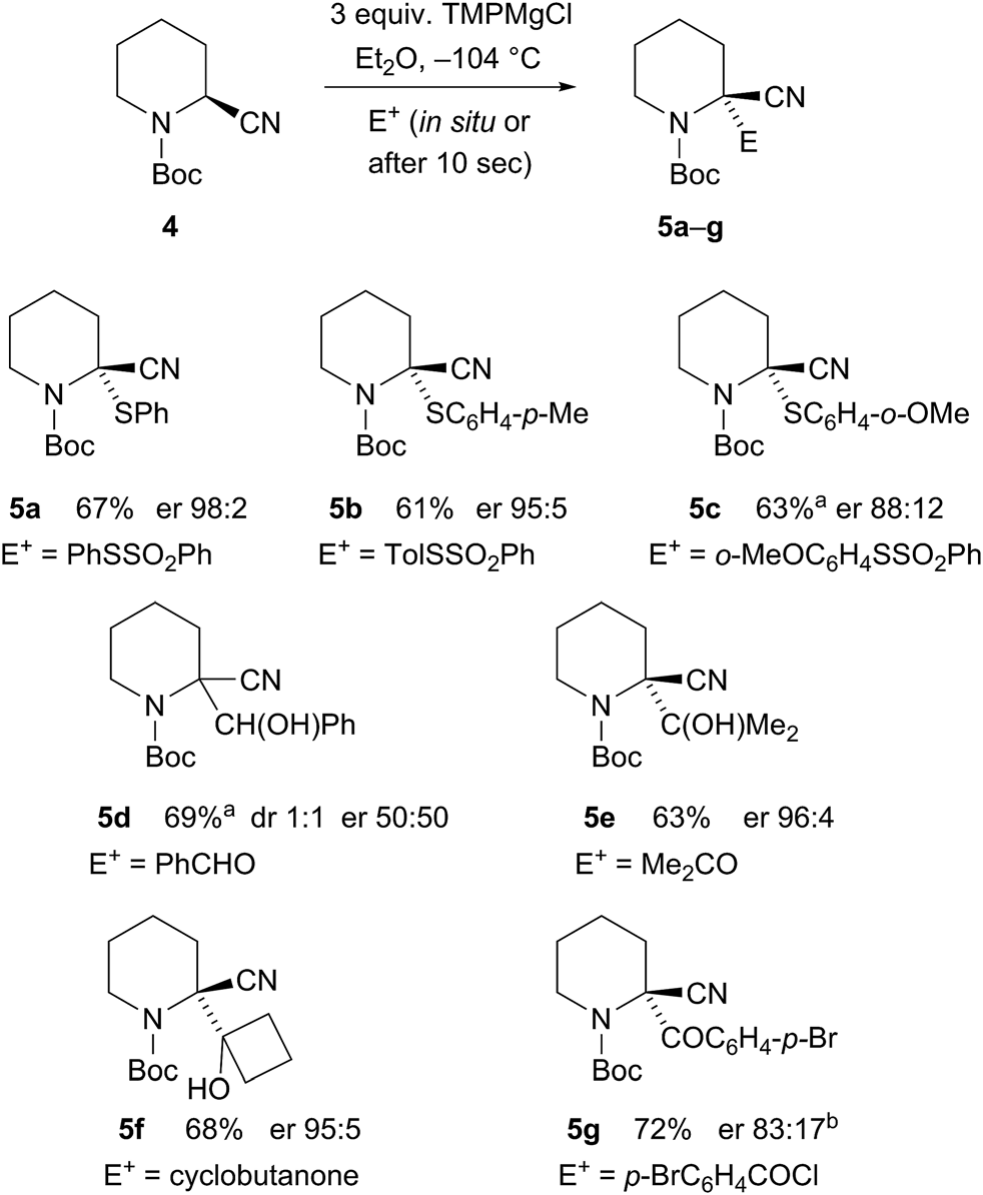
Enantiospecific magnesiation-quench of nitrile **4**. ^a^Reaction in Et_2_O–THF 4 : 1. ^b^Recrystallization gave er 99 : 1.

The kinetic data demonstrate a slightly slower rate for inversion of the intermediate organomagnesium species in Et_2_O. In the case of the magnesiated nitrile **4**, the enantiomerisation half-life *t*_1/2_ ∼ 3 min in Et_2_O and only ∼2 min in THF/Et_2_O, presumably as THF helps to solvate the magnesium cation. Therefore, we carried out the deprotonation in pure Et_2_O and were pleased to find that this improved the enantioselectivity of the metallation–electrophilic quench reaction (Scheme 2). The optimised conditions involved rapid addition of three equivalents of TMPMgCl (prepared from i-PrMgCl and TMPH in Et_2_O) to the nitrile **4** in Et_2_O at –104 °C, either with the electrophile added *in situ* pre-mixed with the nitrile **4** (in the case of the *S*-aryl benzenesulfonates) or with the electrophile added after about 10 s (for the carbonyl electrophiles).

High enantioselectivities of the arylthio derivatives **5a–c** were obtained by the *in situ* method. With the *ortho*-methoxy compound **5c**, the electrophile was only partially soluble in pure Et_2_O, so this reaction was carried out with some THF and this may account for the reduced selectivity. The organomagnesium intermediate has sufficient configurational stability to allow its formation followed by electrophilic quench without the need for an *in situ* electrophile. Benzaldehyde provided racemic product **5d**, possibly due to single electron transfer. However, acetone gave highly enantioenriched alcohol **5e** and cyclobutanone gave the alcohol **5f** also with excellent er. The electrophile *p*-bromobenzoyl chloride gave the nitrile **5g** with er 83 : 17 after 10 s quench and similar selectivity with *in situ* quench.

**Fig. 5:**
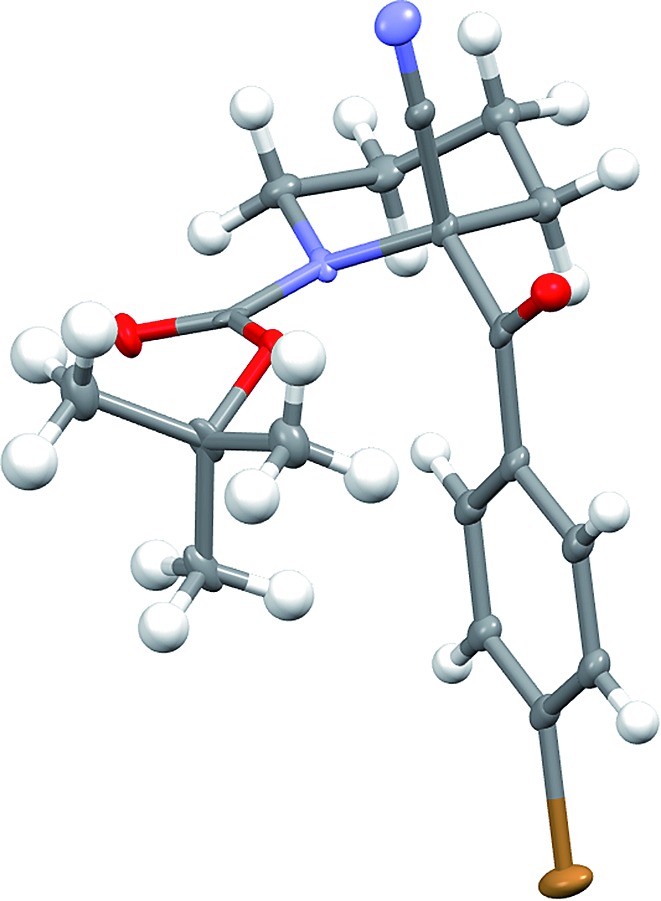
X-ray of nitrile **5g** (er 99 : 1), thermal ellipsoids at 50% calculated displacement parameter.

**Scheme 3:**
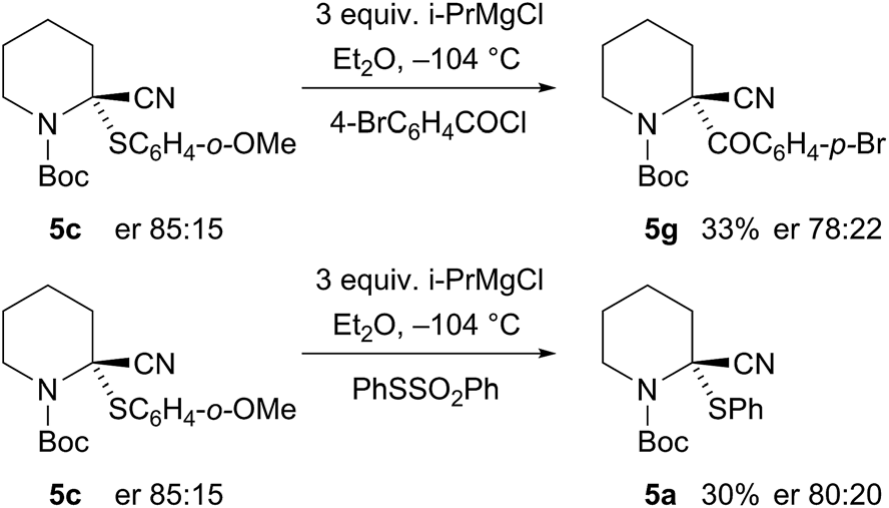
Sulfur–magnesium exchange and electrophilic quench to determine the absolute configuration of nitrile **5c**.

Recrystallisation of the nitrile **5g** gave essentially enantiopure compound (er 99 : 1 by CSP-HPLC) and the absolute configuration was determined by single crystal X-ray analysis (Fig. 5).† This demonstrated that the electrophilic quench occurred with retention of configuration. To determine the absolute configuration of the sulfides, we carried out sulfur–magnesium exchange with i-PrMgCl.^16^ This transformation has not to our knowledge been reported with an enantioenriched sulfide and it was intriguing to discover whether it would be possible to transfer chirality by this method. Addition of i-PrMgCl to the sulfide **5c** in Et_2_O at –104 °C followed by addition of *p*-bromobenzoyl chloride gave the product **5g** in moderate yield and only partial loss of enantioselectivity (Scheme 3). The major enantiomer of the product **5g** had the same configuration as that obtained by the direct addition of *p*-bromobenzoyl chloride, thereby demonstrating that the sulfide **5c** (and hence also likely the sulfides **5a** and **5b**) has the stereochemistry as shown, and was formed by reaction with retention of configuration. We have not been able to determine the absolute configurations of the alcohols **5e** and **5f**, but these are likely to be as shown with reaction by retention of configuration, and this would be in line with other known electrophilic quenches of metallated *N*-Boc-piperidines.^17^ A similar reaction was carried out, in which sulfur–magnesium exchange was followed, after 10 s, by addition of the electrophile PhSSO_2_Ph to give the product **5a** (Scheme 3). This was formed in moderate yield without significant loss of enantiopurity, together with what appeared to be an alkene by-product from elimination.

The impressive enantioselectivities that can be obtained with the simple *N*-Boc-2-cyanopiperidine **4** and base TMPMgCl demonstrate that this method has potential for asymmetric synthesis. The magnesium metal likely has a preference for attachment to the carbon atom at least initially. A possible intermediate, supported by analogy to that proposed by Carlier and co-workers for magnesiated cyclopropylnitriles,^15^ would have two magnesium atoms, one on the carbon atom and one on the nitrile nitrogen atom, connected by a bridging chloride. Dimeric magnesium amides with bridging chloride ligands are well known.^18,19^

To investigate this further, Density Functional Theory (DFT) calculations were performed [6-311G(d,p) basis set with B3LYP functional: see Computational methods section below]. Our calculations show, as expected, that nitrile **4** should be present as two rotamers corresponding to different orientations of the Boc group with approximate equal probability. The rate of rotation of the Boc group is slow at the temperatures used for the reaction. Upon deprotonation this will lead to either the C=O group or the C–O^*t*^Bu group pointing towards the deprotonated carbon. Complexes of these rotamers, with a bridging chloride ligand between two magnesium ions, were found and are shown in the ESI.† These complexes have very different conformations for the two rotamers. In the case where the O^*t*^Bu group points towards the magnesium, a ring-flip occurs to provide a lower energy structure in which the Boc group sits on the opposite side of the ring from the magnesium.

**Fig. 6:**
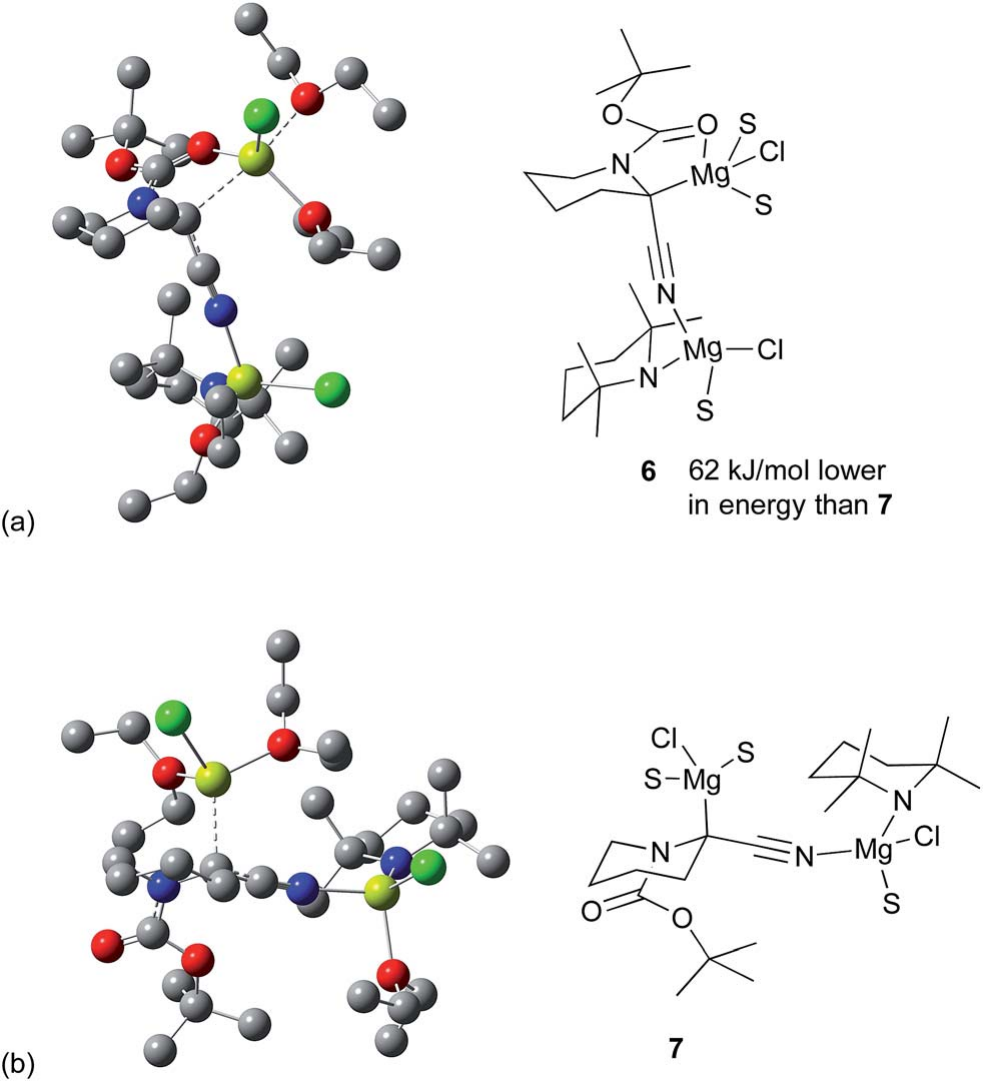
Possible magnesiated intermediates by DFT [6-311G(d,p) basis set with B3LYP functional] and their ChemDraw representations. (a) **6**, chelated structure. (b) **7** non-chelated structure. All hydrogens have been removed for reasons of clarity.

The case where an additional Et_2_O coordinates at the proximal magnesium was also considered. In contrast to that of Carlier and co-workers,^15^ this leads to an additional stabilization energy and opens up the possibility of structures without the bridging Cl atom, which could be expected to be less strained. The two rotamers with the lowest energy are shown in Fig. 6. Other orientations were attempted as well, but all lead to higher energy structures. Fig. 6a (structure **6**) has chelation of the C=O group to the magnesium. Fig. 6b (structure **7**) derives from the other rotamer without this chelation and this structure is 62 kJ mol^–1^ higher in Gibbs energy than **6**. As the Boc group is not rotating at low temperature, both species should be present in solution and able to react with the electrophiles as shown earlier.

The organomagnesium species **6** and **7** could racemise by breakage of the C–Mg bond followed by carbanion inversion and reattachment of the magnesium to the opposite face. Alternatively racemisation could take place by formation of the *N*-magnesiated ketene imine type structure. However, by whatever mechanism racemisation occurs, the experimental data show that the C-magnesiated intermediates have sufficient lifetime at low temperature for addition of an electrophile and reaction to give highly enantiomerically enriched products.

## Experimental

A representative method for the deprotonation and quench of nitrile **4** is given below. For further details and all data, see ESI.†

TMPMgCl (1.6 mL, 0.75 mmol) was added to the nitrile **4** (54 mg, 0.25 mmol) in Et_2_O (1 mL) at –104 °C. After 10 s, cyclobutanone (0.056 mL, 0.75 mmol) was added. After 30 min, saturated aqueous NH_4_Cl (0.3 mL) was added. The mixture was allowed to warm to room temperature and was extracted with Et_2_O (3 × 1 mL), dried (MgSO_4_) and the solvent was evaporated. Purification by column chromatography on silica gel, eluting with petrol–EtOAc (9 : 1), gave the alcohol **5f** (47 mg, 68%); [*α*]21D –25.7 (*c* 0.4, CHCl_3_); er 95 : 5 by CSP-GC.

## Computational methods

All calculations were performed using the D.01 version of Gaussian 09.^20^ Density functional theory was used throughout using the B3LYP^21^ functional including dispersion interactions *via* the GD3-BJ^22^ correction. All calculations used the 6-311G(d,p)^23^ basis set. Solvent was included *via* the PCM method^24^ as implemented in Gaussian with the default parameters for Et_2_O. Frequency calculations were performed on all optimized structures to confirm that these were all true minima as evidenced by the absence of imaginary frequencies. No complete conformational search for any added Et_2_O molecule was performed. Instead, the calculations were all started with Et_2_O in the conformation of its free molecule. All Gibbs energies were evaluated at 298.15 K.

## Conclusions

In conclusion, the base TMPMgCl can be used at low temperatures to deprotonate a chiral nitrile without significant loss of enantiopurity even in the absence of an *in situ* electrophile. The intermediate magnesiated nitrile can be trapped with a variety of electrophiles to give enantioenriched substituted nitrile products with overall retention of configuration. The organomagnesium intermediate racemises fairly rapidly and the half-life is slightly slower in the presence of the less polar solvent Et_2_O than in THF. In addition we have shown that sulfur–magnesium exchange can occur with retention of configuration. Calculations support the experimental that two magnesium ions are present in the intermediate complexes. These results suggest that, despite their general lack of use for asymmetric synthesis, chiral nitrile anions can be valuable intermediates that do not always lose their configuration but can be converted to highly enantiomerically enriched products.

## References

[cit1] Enders D., Kirchhoff J., Gerdes P., Mannes D., Raabe G., Runsink J., Boche G., Marsch M., Ahlbrecht H., Sommer H., Fleming F., Shook B., Opatz T., Otto N., Opatz T., López R., Palomo C. (1998). Eur. J. Org. Chem..

[cit2] Purzycki M., Liu W., Hilmersson G., Fleming F. F. (2013). Chem. Commun..

[cit3] Carlier P. R., Lo C. W. S. (2000). J. Am. Chem. Soc..

[cit4] Boche G., Marsch M., Harms K. (1986). Angew. Chem., Int. Ed..

[cit5] Sott R., Granander J., Hilmersson G. (2004). J. Am. Chem. Soc..

[cit6] Sasaki M., Kawanishi E., Shirakawa Y., Kawahata M., Masu H., Yamaguchi K., Takeda K. (2008). Eur. J. Org. Chem..

[cit7] For examples of forming enantiomerically enriched nitriles from silyl ketene imines, see Denmark S. E. Wilson T. W. Burk M. T., Chem.–Eur. J., 2014, 20 , 9268 Guin J. Varseev G. List B., J. Am. Chem. Soc., 2013, 135 , 2100 Zhao J. Fang B. Luo W. Hao X. Liu X. Lin L. Feng X., Angew. Chem., Int. Ed., 2015, 54 , 241 .25043283

[cit8] For examples of forming enantiomerically enriched products from racemic nitriles in the presence of an electrophile and a chiral catalyst, see Shirakawa S. Liu K. Ito H. Le T. N. Maruoka K., Adv. Synth. Catal., 2011, 353 , 2614 Trost B. M. Miller J. R. Hoffman C. M., J. Am. Chem. Soc., 2011, 133 , 8165 Breistein P. Johansson J. Ibrahem I. Lin S. Deiana L. Sun J. Cordova A., Adv. Synth. Catal., 2012, 354 , 1156 Yin L. Kanai M. Shibasaki M., Tetrahedron, 2012, 68 , 3497 Ohmatsu K. Goto A. Ooi T., Chem. Commun., 2012, 48 , 7913 Qin T.-Y. Liao W.-W. Zhang Y.-J. Zhang S. X.-A., Org. Biomol. Chem., 2013, 11 , 984 Eitel S. H. Jautze S. Frey W. Peters R., Chem. Sci., 2013, 4 , 2218 Sureshkumar D. Ganesh V. Kumagai N. Shibasaki M., Chem.–Eur. J., 2014, 20 , 15723 Badiola E. Fiser B. Gómez-Bengoa E. Mielgo A. Olaizola I. Urruzuno I. García J. M. Odriozola J. M. Razkin J. Oiarbide M. Palomo C., J. Am. Chem. Soc., 2014, 136 , 17869 Turnbull B. W. H. Evans P. A., J. Am. Chem. Soc., 2015, 137 , 6156 Hu Z.-P. Zhuang Z. Liao W.-W., J. Org. Chem., 2015, 80 , 4627 Vita M. V. Caramenti P. Waser J., Org. Lett., 2015, 17 , 5832 Izquierdo J. Landa A. Bastida I. López R. Oiarbide M. Palomo C., J. Am. Chem. Soc., 2016, 138 , 3282 Zou G.-F. Zhuang S.-Q. Wang J.-X. Liao W.-W., J. Org. Chem., 2016, 81 , 5717 .

[cit9] Sasaki M., Takegawa T., Ikemoto H., Kawahata M., Yamaguchi K., Takeda K. (2012). Chem. Commun..

[cit10] Sasaki M. Takegawa T. Sakamoto K. Kotomori Y. Otani Y. Ohwada T. Kawahata M. Yamaguchi K. Takeda K., Angew. Chem., Int. Ed., 2013, 52 , 12956 , . See also, Kotomori Y. Sasaki M. Kawahata M. Yamaguchi K. Takeda K., J. Org. Chem., 2015, 80 , 11013 . 10.1002/anie.201306443 24123536

[cit11] Barker G., Alshawish M. R., Skilbeck M. C., Coldham I. (2013). Angew. Chem., Int. Ed..

[cit12] Carlier P. R., Zhang Y. (2007). Org. Lett..

[cit13] Fleming F. F., Wei Y., Liu W., Zhang Z. (2008). Tetrahedron.

[cit14] Yang X., Nath D., Fleming F. F. (2015). Org. Lett..

[cit15] Gao M., Patwardhan N. N., Carlier P. R., Patwardhan N. N., Gao M., Carlier P. R. (2013). J. Am. Chem. Soc..

[cit16] Nath D. Skilbeck M. C. Coldham I. Fleming F. F., Org. Lett., 2014, 16 , 62 , . See also, Rayner P. J. O'Brien P. Horan R. A. J., J. Am. Chem. Soc., 2013, 135 , 8071 Nath D. Fleming F. F., Chem.–Eur. J., 2012, 19 , 2023 . 24328754 10.1021/ol403020s

[cit17] Sheikh N. S., Leonori D., Barker G., Firth J. D., Campos K. R., Meijer A. J. H. M., O'Brien P., Coldham I. (2012). J. Am. Chem. Soc..

[cit18] Armstrong D. R., García-Álvarez P., Kennedy A. R., Mulvey R. E., Parkinson J. A. (2010). Angew. Chem., Int. Ed..

[cit19] Neufeld R., Teuteberg T. L., Herbst-Irmer R., Mata R. A., Stalke D. (2016). J. Am. Chem. Soc..

[cit20] Frisch M. J., Trucks G. W., Schlegel H. B., Scuseria G. E., Robb M. A., Cheeseman J. R., Scalmani G., Barone V., Mennucci B., Petersson G. A., Nakatsuji H., Caricato M., Li X., Hratchian H. P., Izmaylov A. F., Bloino J., Zheng G., Sonnenberg J. L., Hada M., Ehara M., Toyota K., Fukuda R., Hasegawa J., Ishida M., Nakajima T., Honda Y., Kitao O., Nakai H., Vreven T., Montgomery Jr J. A., Peralta J. E., Ogliaro F., Bearpark M., Heyd J. J., Brothers E., Kudin K. N., Staroverov V. N., Kobayashi R., Normand J., Raghavachari K., Rendell A., Burant J. C., Iyengar S. S., Tomasi J., Cossi M., Rega N., Millam J. M., Klene M., Knox J. E., Cross J. B., Bakken V., Adamo C., Jaramillo J., Gomperts R., Stratmann R. E., Yazyev O., Austin A. J., Cammi R., Pomelli C., Ochterski J. W., Martin R. L., Morokuma K., Zakrzewski V. G., Voth G. A., Salvador P., Dannenberg J. J., Dapprich S., Daniels A. D., Farkas Ö., Foresman J. B., Ortiz J. V., Cioslowski J. and Fox D. J., Gaussian 09, Revision D.01, Gaussian, Inc., Wallingford CT, 2009.

[cit21] Becke A. D. (1993). J. Chem. Phys..

[cit22] Grimme S., Ehrlich S., Goerigk L. (2011). J. Comput. Chem..

[cit23] McLean A. D., Chandler G. S., Raghavachari K., Binkley J. S., Seeger R., Pople J. A. (1980). J. Chem. Phys..

[cit24] Scalmani G. Frisch M. J., J. Chem. Phys., 2010, 132 , 114110 Cossi M. Rega N. Scalmani G. Barone V., J. Comput. Chem., 2003, 24 , 669 , and references therein . 20331284 10.1063/1.3359469

