# Supplementary material for: Highly enantioselective metallation–substitution alpha to a chiral nitrile
Source: Chem Sci. 2016 Oct 25;8(2):1436–41. doi: 10.1039/c6sc03712g (PMC5460602; doi:10.1039/c6sc03712g)
Supplement: Supplementary file 1 [file SC-008-C6SC03712G-s001.pdf]

Electronic Supplementary Material (ESI) for Chemical Science.
This journal is © The Royal Society of Chemistry 2016

Electronic Supplementary Information (ESI)

Highly enantioselective metallation–substitution alpha to a chiral nitrile

Arghya Sadhukhan, Melanie C. Hobbs, Anthony J. H. M. Meijer and Iain Coldham*

Contents

1. Experimental Details 2
   1.1 General 2
   1.2 Experimental Procedures and Characterisation Data 2
2. In situ IR spectra 10
3. Kinetics of enantiomerization 11
4. X-ray data for compound **5g** 13
5. DFT data 27
6. HPLC/GC traces 69
7. $^{1}H/^{13}C$ NMR spectra 77

1. Experimental details

1.1 General

All reagents were obtained from commercial suppliers and were used without further purification unless otherwise specified. Solvents were purified using a Grubbs dry solvent system (model SPS-200-6). Petrol refers to petroleum ether (b.p. 40–60 °C). Reactions were carried out under N_2 using oven-dried and/or flame-dried glassware. Thin layer chromatography was performed on silica plates and visualised by UV irradiation at 254 nm or by staining with an alkaline $KMnO_4$ dip. Column chromatography was performed using silica gel (40–63 micron mesh). Infrared spectra were recorded on Perkin Elmer Spectrum RX Fourier Transform IR System. In situ ReactIR™ infra-red spectroscopic monitoring was performed on a Mettler-Toledo React-IR 4000 spectrometer equipped with a diamond-tipped (DiComp) probe. ^{1}H NMR spectra were recorded on a Bruker AC400 (400 MHz) instrument. Chemical shifts are reported in ppm with respect to the residual solvent peaks, with multiplicities given as s = singlet, d = doublet, t = triplet, q = quartet, m = multiplet. Coupling constants, *J*, are quoted to the nearest 0.5 Hz. ^{13}C NMR were recorded on the above instrument at 100 MHz. Low and high resolution (accurate mass) mass spectra were recorded on a Walters LCT instrument for Electro–Spray (ES). Chiral stationary phase (CSP) HPLC was performed on a Gilson instrument and a multiple wavelength, UV/Vis diode array detector; integration was performed at 254 nm. Gas chromatography (CSP GC) was performed on a Perkin Elmer Arnel Autosystem XL GC using an Astec CHIRALDEX Beta cyclodextrin, permethyl fused silica capillary column (30 m x 0.25 mm).

^{i}PrMgCl (2.0 M in THF or diethyl ether) was obtained commercially and used without further purification. TMPMgCl (0.5 M in THF or diethyl ether) was prepared by addition of ^{i}PrMgCl to TMPH (2,2,6,6-tetramethylpiperidine) according to A. Krasovskiy, V. Krasovskaya, P. Knochel, *Angew. Chem. Int. Ed.* **2006**, *45*, 2958.

1.2 Experimental Procedures and Characterisation Data

General Procedure A: LDA mediated metallation–trapping

n-BuLi (2.5 M solution in hexanes, 1.1 eq) was added dropwise to a stirred solution of diisopropylamine (1.2 eq.) in THF at –25 °C under N_2. After 30 min, the mixture was cooled to –78 °C and the nitrile **4** (1.0 eq.) was added. After 10 min, the electrophile (1.2 eq.) was added. The resulting solution was stirred for 30 min, then warmed to room temperature. Saturated aqueous NH_4Cl (2 mL) was added, the layers were separated, and the aqueous layer was extracted with Et_2O (3 × 10 mL). The combined organic layers were dried (Na_2SO_4) and evaporated under reduced pressure.

General Procedure B: TMPMgCl mediated *in situ* metallation–trapping

TMPMgCl (3.0 eq.) prepared in Et_2O (2.5 mL) or THF–Et_2O (2.5 mL, 1:1) was added via a syringe to a stirred solution of nitrile **4** (1 eq.) and electrophile (3 eq.) in Et_2O (0.5 mL) at –104 °C under N_2. After 30 min, saturated aqueous NH_4Cl (4 mL) was added. The resulting solution was allowed to

warm to room temperature and the layers were separated. The aqueous layer was extracted with Et_2O (3 × 10 mL). The combined organic layers were dried (Na_2SO_4) and evaporated under reduced pressure.

General Procedure C: TMPMgCl mediated metallation–trapping

TMPMgCl (3.0 eq.) prepared in Et_2O (2.5 mL) or THF–Et_2O (2.5 mL, 1:1) was added rapidly via a syringe to the nitrile **4** (1.0 eq.) in Et_2O (0.5 mL) at –104 °C under N_2. After 10 sec, the electrophile (3.0 eq.) was added in one portion. After 30 min, saturated aqueous NH_4Cl (4 mL) was added. The resulting solution was allowed to warm to room temperature and the layers were separated. The aqueous layer was extracted with Et_2O (3 × 10 mL). The combined organic layers were dried ($MgSO_4$) and evaporated under reduced pressure.

***tert*-Butyl (*S*)-2-Cyanopiperidin-1-carboxylate 4**

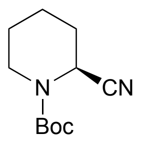

4

Ethyl chloroformate (4.2 mL, 44 mmol) was added to commercial (*S*)-*N*-Boc-pipecolic acid (4.5 g, 20 mmol) and triethylamine (2.7 mL, 20 mmol) in THF (30 mL) at −15 °C. After 1 h, 35% ammonia solution in water (8 mL) was added. After 16 h, the solvent was evaporated and the residue was taken up in EtOAc (100 mL) and washed with 10% citric acid (100 mL), $NaHCO_3$ (100 mL) and brine (100 mL), dried ($MgSO_4$) and the solvent was evaporated to give the primary amide (4.15 g, 93%) as an amorphous solid; $[\alpha]^D{}_{23}$ −112 (1, $CHCl_3$); m.p. 89−91 °C; IR ν_{max} (film)/cm^{-1} 3380, 3180, 2945, 1680; ^1H NMR (400 MHz, $CDCl_3$) δ = 6.05 (1H, br s), 5.80 (1H, m), 4.81−4.69 (1H, m), 4.13−3.95 (1H, m), 2.87−2.76 (1H, m), 2.32−2.22 (1H, m), 1.68−1.56 (2H, m), 1.54−1.36 (3H, m), 1.47 (9H, s); ^{13}C NMR (100 MHz, $CDCl_3$) δ = 174.2, 155.5, 80.7, 54.2, 42.4 (CH_2), 28.5, 25.5, 25.0, 20.6; HRMS (ES) Found MH^+ 229.1560. $C_{11}H_{21}N_2O_3$ requires MH^+, 229.1552.

Trifluoroacetic anhydride (2.9 mL, 21 mmol) was added to the amide prepared as above (4.0 g, 17.5 mmol) and triethylamine (5.8 mL, 42 mmol) in THF (60 mL) at 0 °C. The mixture was stirred at room temperature for 18 h, then water (30 mL) was added and the solvent was evaporated. The residue was taken up in CH_2Cl_2 (100 mL), washed with aqueous HCl (100 mL, 0.1 M) and aqueous NaOH (100 mL, 0.1 M), dried ($MgSO_4$) and the solvent was evaporated. Purification by column chromatography on silica gel, eluting with petrol−EtOAc (4:1), gave nitrile **4** (2.98 g, 81%) as a solid; $[\alpha]^D{}_{21}$ −130 (1, $CHCl_3$); m.p. 79−80 °C; R_f [petrol−EtOAc (4:1)] 0.74; ^1H NMR (400 MHz, $CDCl_3$) δ = 5.40−5.08 (1H, m), 4.15−3.95 (1H, m), 3.04−2.83 (1H, m), 1.97−1.56 (6H, m), 1.47 (9H, s); ^{13}C NMR (100 MHz, $CDCl_3$) δ = 154.1, 117.8, 81.5, 44.2, 41.5, 28.6, 28.4, 24.7, 20.4; HRMS (ES) Found MH^+ 211.1438. $C_{11}H_{19}N_2O_2$ requires MH^+, 211.1447.

***tert*-Butyl 2-Cyano-2-(2-phenylsulfanyl)piperidin-1-carboxylate 5a**

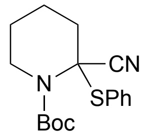

5a

Racemic method (general procedure A):

n-BuLi (0.44 mL, 1.1 mmol, 2.5 M in hexanes) was added dropwise to diisopropylamine (0.17 mL, 1.2 mmol) in THF (2 mL) at –78 °C under N_2. After 10 min, the nitrile **4** (0.21 g, 1 mmol) was added. After 10 min, $PhSSO_2Ph$ (0.275 g, 1.2 mmol) was added. The resulting solution was stirred for 30 min, then warmed to room temperature. Saturated aqueous NH_4Cl (2 mL) was added, the layers were separated, and the aqueous layer was extracted with Et_2O (3 × 10 mL). The combined organic layers were dried (Na_2SO_4) and evaporated under reduced pressure. Purification by column chromatography on silica gel, eluting with petrol−EtOAc (9:1), gave the nitrile **5a** (251 mg, 79%) as an oil; R_f [petrol−EtOAc (4:1)] 0.50; FT-IR ν_{max} (film)/cm^{-1} 2930, 2865, 1705; ^{1}H NMR (400 MHz, $CDCl_3$) δ = 7.72–7.69 (2H, m), 7.48–7.35 (3H, m), 4.14−4.10 (1H, m), 3.23 (1H, td, *J* 13, 3), 2.30−2.12 (2H, m), 1.98−1.70 (2H, m), 1.58−1.52 (2H, m), 1.35 (9H, s); ^{13}C NMR (100 MHz, $CDCl_3$) δ = 153.5, 137.4, 130.4, 129.5, 129.3, 119.4, 83.0, 62.7, 40.4, 37.5, 27.9, 23.9, 19.1; HRMS (ES) Found MH^+ 319.1495. $C_{17}H_{22}N_2O_2S$ requires MH^+, 319.1480. The enantiomers were resolved by chiral stationary phase HPLC using an AD column with 0.5% ^{i}PrOH in hexanes at 1 mL/min, detection at 254 nm, retention times 10.3 and 11.3 min.

Asymmetric method (general procedure B):

TMPMgCl (1.6 mL, 0.75 mmol) was added to premixed nitrile **4** (54 mg, 0.25 mmol) and $PhSSO_2Ph$ (171 mg, 0.75 mmol) in Et_2O (1 mL) at −104 °C. After 30 min, saturated aqueous NH_4Cl (0.3 mL) was added. The mixture was extracted with Et_2O (3 × 1 mL), dried ($MgSO_4$) and the solvent was evaporated. Purification by column chromatography on silica gel, eluting with petrol−EtOAc (9:1), gave the nitrile **5a** (53 mg, 67%); $[\alpha]^{21}_D$ –21.6 (0.5, $CHCl_3$); data as above. The enantiomer ratio was determined to be 98:2 by CSP-HPLC (major enantiomer eluted at 11.7 min and minor enantiomer at 10.2 min).

Method from the sulfide **5c**

^{i}PrMgCl (0.375 mL, 0.75 mmol, 2 M in Et_2O) was added to the nitrile **5c** (87 mg, 0.25 mmol, er 85:15) in Et_2O (2 mL) at −104 °C. After 10 sec, $PhSSO_2Ph$ (171 mg, 0.75 mmol) in Et_2O (1 mL) was added at −104 °C. After 30 min, saturated aqueous NH_4Cl (0.3 mL) was added. The mixture was extracted with Et_2O (3 × 1 mL), dried ($MgSO_4$) and the solvent was evaporated. Purification by column chromatography on silica gel, eluting with petrol−EtOAc (9:1), gave the nitrile **5a** (24 mg, 30%); $[\alpha]^{21}_D$ –16.0 (0.4, $CHCl_3$); data as above. The enantiomer ratio was determined to be 80:20 by CSP-HPLC.

***tert*-Butyl 2-Cyano-2-(*p*-tolylsulfanyl)piperidin-1-carboxylate 5b**

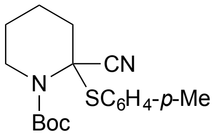

5b

Racemic method (general procedure A):

n-BuLi (0.44 mL, 1.1 mmol, 2.5 M in hexanes) was added dropwise to diisopropylamine (0.17 mL, 1.2 mmol) in THF (2 mL) at –78 °C under N_2. After 10 min, the nitrile **4** (0.21 g, 1 mmol) was added. After 10 min, *p*-TolylSSO$_2$Ph (316 mg, 1.2 mmol) was added. The resulting solution was stirred for 30 min, then warmed to room temperature. Saturated aqueous NH_4Cl (2 mL) was added, the layers were separated, and the aqueous layer was extracted with Et_2O (3 × 10 mL). The combined organic layers were dried (Na_2SO_4) and evaporated under reduced pressure. Purification by column chromatography on silica gel, eluting with petrol−EtOAc (9:1), gave the nitrile **5b** (259 mg, 78%) as an oil; R_f [petrol−EtOAc (4:1)] 0.50; FT-IR ν_{max} (film)/cm^{-1} 2930, 2865, 1705; ^{1}H NMR (400 MHz, $CDCl_3$) δ = 7.59 (2H, d, *J* 8), 7.35–7.22 (2H, m), 4.14–4.10 (1H, m), 3.23 (1H, td, *J* 13, 3), 2.39 (3H, s), 2.29–2.24 (1H, m), 2.19–2.11 (1H, m), 2.00–1.70 (4H, m), 1.36 (9H, s); ^{13}C NMR (100 MHz, CDCl3) δ = 152.6, 139.8, 136.3, 129.1, 124.9, 118.5, 81.9, 61.7, 39.3, 36.3, 28.7, 26.9, 22.9, 20.3; HRMS (ES) Found MH$^+$ 333.1634. $C_{18}H_{24}N_2O_2S$ requires MH$^+$, 333.1631. The enantiomers were resolved by chiral stationary phase HPLC using an AD column with 0.5% ^{i}PrOH in hexanes at 1 mL/min, detection at 254 nm, retention times 10.3 and 12.0 min.

Asymmetric method (general procedure B):

TMPMgCl (1.6 mL, 0.75 mmol) was added to premixed nitrile **4** (54 mg, 0.25 mmol) and *p*-TolylSSO$_2$Ph (198 mg, 0.75 mmol) in Et_2O (1 mL) at −104 °C. After 30 min, saturated aqueous NH_4Cl (0.3 mL) was added. The mixture was extracted with Et_2O (3 × 1 mL), dried ($MgSO_4$) and the solvent was evaporated. Purification by column chromatography on silica gel, eluting with petrol−EtOAc (9:1), gave the nitrile **5b** (56 mg, 68%); $[\alpha]^{21}_D$ –24.9 (0.4, $CHCl_3$); data as above. The enantiomer ratio was determined to be 95:5 by CSP-HPLC (major enantiomer eluted at 12.8 min and minor enantiomer at 10.7 min).

***tert*-Butyl 2-Cyano-2-(*o*-methoxyphenylsulfanyl)piperidin-1-carboxylate 5c**

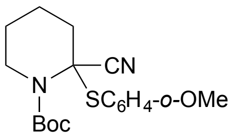

5c

Racemic method (general procedure A):

n-BuLi (0.44 mL, 1.1 mmol, 2.5 M in hexanes) was added dropwise to diisopropylamine (0.17 mL, 1.2 mmol) in THF (2 mL) at –78 °C under N_2. After 10 min, the nitrile **4** (0.21 g, 1 mmol) was added. After 10 min, *o*-OMePhSSO$_2$Ph (336 mg, 1.2 mmol) was added. The resulting solution was stirred for 30 min, then warmed to room temperature. Saturated aqueous NH_4Cl (2 mL) was added, the layers were separated, and the aqueous layer was extracted with Et_2O (3 × 10 mL). The combined organic

layers were dried (Na_2SO_4) and evaporated under reduced pressure. Purification by column chromatography on silica gel, eluting with petrol−EtOAc (9:1), gave the nitrile **5b** (254 mg, 73%) as an oil; R_f [petrol−EtOAc (4:1)] 0.40; FT-IR ν_{max} (film)/cm^{-1} 2930, 2860, 1725; ^{1}H NMR (400 MHz, $CDCl_3$) δ = 7.79 (1H, dd, *J* 7.5, 1.5), 7.47 (1H, td, *J* 7.5, 1.5), 7.03 (1H, td, *J* 7.5, 1), 6.93 (1H, dd, *J* 7.5, 1), 4.13−4.10 (1H, m), 3.88 (3H, s), 3.39 (1H, td, *J* 13, 4), 2.38−2.32 (1H, m), 2.22 (1H, td, *J* 13, 4), 2.03–1.93 (1H, m), 1.82−1.71 (2H, m), 1.57−1.47 (1H, m), 1.32 (9H, s); ^{13}C NMR (100 MHz, $CDCl_3$) δ = 160.3, 152.6, 139.3, 131.4, 120.2, 118.4, 116.7, 81.7, 62.0, 54.6, 39.2, 37.3, 28.7, 26.8, 22.9; HRMS (ES) Found MH$^+$ 349.1581. $C_{18}H_{24}N_2O_3S$ requires MH$^+$, 349.1580. The enantiomers were resolved by chiral stationary phase HPLC using an AD column with 0.5% ^{i}PrOH in hexanes at 1 mL/min, detection at 254 nm, retention times 14.9 and 17.0 min.

Asymmetric method (general procedure B):

TMPMgCl (1.6 mL, 0.75 mmol) was added to premixed nitrile **4** (54 mg, 0.25 mmol) and *o*-OMePhSSO$_2$Ph (210 mg, 0.75 mmol) in Et_2O (1 mL) at −104 °C. After 30 min, saturated aqueous NH_4Cl (0.3 mL) was added. The mixture was extracted with Et_2O (3 × 1 mL), dried ($MgSO_4$) and the solvent was evaporated. Purification by column chromatography on silica gel, eluting with petrol−EtOAc (9:1), gave the nitrile **5c** (55 mg, 63%); $[\alpha]^{21}_D$ –22.0 (0.4, $CHCl_3$); data as above. The enantiomer ratio was determined to be 88:12 by CSP-HPLC (major enantiomer eluted at 17.2 min and minor enantiomer at 15.0 min).

tert-Butyl 2-Cyano-2-[hydroxy(phenyl)methyl]piperidine-1-carboxylate 5d

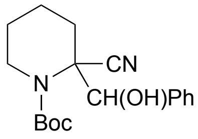

5d

n-BuLi (0.44 mL, 1.1 mmol, 2.5 M in hexanes) was added dropwise to diisopropylamine (0.17 mL, 1.2 mmol) in THF (2 mL) at –25 °C under N_2. After 30 min, the mixture was cooled to –78 °C and the nitrile **4** (0.21 g, 1 mmol) was added. After 10 min, benzaldehyde (0.116 g, 1.1 mmol) was added. The resulting solution was stirred for 30 min, then warmed to room temperature. Saturated aqueous NH_4Cl (2 mL) was added, the layers were separated, and the aqueous layer was extracted with Et_2O (3 × 10 mL). The combined organic layers were dried (Na_2SO_4) and evaporated under reduced pressure. Purification by column chromatography on silica gel, eluting with petrol−EtOAc (9:1), gave the nitriles **5d** as separable diastereomers (diastereomer A: 126 mg, 40%, and diastereomer B: 132 mg, 42%) as amorphous solids;

Diastereomer A m.p. 147−149 °C; R_f [petrol−EtOAc (4:1)] 0.30; FT-IR ν_{max} (film)/cm^{-1} 2975, 2115, 1700, 1680; ^{1}H NMR (400 MHz, $CDCl_3$) δ = 7.42−7.36 (5H, m), 5.60 (1H, s), 4.72 (1H, s), 3.71−3.65 (2H, m), 2.40−2.45 (1H, m), 2.04−1.96 (1H, m), 1.78−1.72 (4H, m), 1.61 (9H, s); ^{13}C NMR (100 MHz, $CDCl_3$) δ = 154.9, 137.5, 128.5, 128.2, 127.5, 119.8, 82.4, 74.1, 62.9, 39.7, 28.4, 27.4, 21.4, 16.6; HRMS (ES) Found MNa$^+$ 339.1682. $C_{18}H_{24}N_2O_3Na$ requires MNa$^+$, 339.1679. The enantiomers were resolved by chiral stationary phase HPLC using a Cellulose-2 column with 10% ^{i}PrOH in hexanes at 1 mL/min, detection at 220 nm, retention times 5.6 and 6.6 min.

Diastereomer 2 m.p. 139−141 °C; R_f [petrol−EtOAc (4:1)] 0.27; FT-IR ν_{max} (film)/cm^{-1} 2975, 2115, 1695, 1680; ^{1}H NMR (400 MHz, $CDCl_3$) δ = 7.48 (2H, d, *J* 7.5), 7.40−7.33 (3H, m), 5.48 (1H, s), 3.91 (1H, s), 3.86−3.80 (1H, m), 3.00–2.93 (1H, m), 2.29−2.22 (1H, m), 1.82−1.59 (5H, m), 1.56 (9H,

s); ^{13}C NMR (100 MHz, $CDCl_3$) δ = 154.9, 137.5, 128.5, 128.2, 127.6, 119.8, 82.4, 74.1, 63.0, 39.8, 28.4, 27.4, 21.4, 16.6; HRMS (ES) Found MNa^+ 339.1683. $C_{18}H_{24}N_2O_3Na$ requires MNa^+, 339.1679. The enantiomers were resolved by chiral stationary phase HPLC using a Cellulose-1 column with 10% ^iPrOH in hexanes at 1 mL/min, detection at 220 nm, retention times 6.1 and 10.1 min.

tert-Butyl 2-Cyano-2-(2-hydroxypropan-2-yl)piperidin-1-carboxylate 5e

and

1,1-Dimethyl-3-oxo-hexahydro-1H-[1,3]oxazolo[3,4-a]pyridine-8a-carbonitrile 5e'

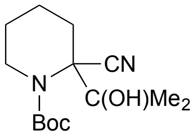

5e

and

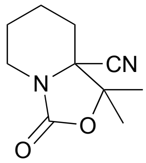

5e'

Racemic method (general procedure A):

n-BuLi (0.44 mL, 1.1 mmol, 2.5 M in hexanes) was added dropwise to diisopropylamine (0.17 mL, 1.2 mmol) in THF (2 mL) at –25 °C under N_2. After 30 min, the mixture was cooled to –78 °C and the nitrile **4** (0.21 g, 1 mmol) was added. After 10 min, acetone (0.088 mL, 1.2 mmol) was added. The resulting solution was stirred for 30 min, then warmed to room temperature. Saturated aqueous NH_4Cl (2 mL) was added, the layers were separated, and the aqueous layer was extracted with Et_2O (3 × 10 mL). The combined organic layers were dried (Na_2SO_4) and evaporated under reduced pressure. Purification by column chromatography on silica gel, eluting with petrol−EtOAc (9:1), gave the nitrile **5e** (120 mg, 45%) as needles and the nitrile **5e'** (54 mg, 28%) as needles;

Data for **5e**:

m.p. 90−91 °C; R_f [petrol−EtOAc (4:1)] 0.27; FT-IR ν_{max} (film)/cm^{-1} 2920, 2855, 1750; ^1H NMR (400 MHz, $CDCl_3$) δ = 5.61 (1H, br s), 4.02−3.96 (1H, m), 3.07−2.95 (1H, m), 2.08−2.05 (1H, m), 1.94−1.82 (3H, m), 1.71−1.62 (2H, m), 1.52 (9H, s), 1.35 (3H, s), 1.27 (3H, s); ^{13}C NMR (100 MHz, $CDCl_3$) δ = 156.6, 118.6, 82.9, 76.6, 67.1, 40.2, 29.8, 28.3, 26.2, 24.5, 20.7, 16.4; HRMS (ES) Found MH^+ 269.1854. $C_{14}H_{25}N_2O_3$ requires MH^+, 269.1865. The enantiomers were resolved by chiral stationary phase GC (CSP-GC) using a Perkin Elmer Arnel Autosystem XL GC using an Astec CHIRALDEX beta cyclodextrin, permethylated fused silica capillary column (30 m x 0.25 mm) at 160 °C and 1.40 mL/min, retention times 8.7 and 8.8 min (see GC trace below).

Data for **5e'**:

m.p. 81−84 °C; R_f [petrol−EtOAc (4:1)] 0.14; FT-IR ν_{max} (film)/cm^{-1} 2950, 2860, 1750; ^1H NMR (400 MHz, $CDCl_3$) δ = 3.92–3.86 (1H, m), 3.05 (1H, td, *J* 13, 3.5), 2.07–1.98 (1H, m), 1.94–1.65 (5H, m), 1.69 (3H, s), 1.43 (3H, s); ^{13}C NMR (100 MHz, $CDCl_3$) δ = 154.9, 116.3, 81.5, 65.4, 40.5, 29.8, 25.5, 23.3, 22.2, 20.9; HRMS (ES) Found MH^+ 195.1126. $C_{10}H_{15}N_2O_2$ requires MH^+, 195.1134; LRMS *m/z* (ES) 195 (100%).

Asymmetric method (general procedure C):

TMPMgCl (1.6 mL, 0.75 mmol) was added to carbamate **4** (54 mg, 0.25 mmol) in Et_2O (1 mL) at −104 °C. After 10 sec, acetone (0.088 mL, 1.2 mmol) was added. After 30 min, saturated aqueous NH_4Cl (0.3 mL) was added. The mixture was extracted with Et_2O (3 × 1 mL), dried ($MgSO_4$) and the solvent was evaporated. Purification by column chromatography on silica gel, eluting with petrol−EtOAc (9:1), gave the nitrile **5e** (42 mg, 63%); $[\alpha]^{21}_D$ −12 (1.0, $CHCl_3$); data as above. The enantiomer ratio was determined to be 96:4 by CSP-GC (major enantiomer eluted at 8.6 min and minor at 9 min).

***tert*-butyl 2-cyano-2-(1-hydroxycyclobutyl)piperidine-1-carboxylate 5f**

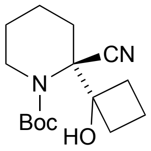

5f

Racemic method (general procedure A):

n-BuLi (0.44 mL, 1.1 mmol, 2.5 M in hexanes) was added dropwise to diisopropylamine (0.17 mL, 1.2 mmol) in THF (2 mL) at –25 °C under N_2. After 30 min, the mixture was cooled to –78 °C and the nitrile **4** (0.21 g, 1 mmol) was added. After 10 min, cyclobutanone (0.09 mL, 1.2 mmol) was added. The resulting solution was stirred for 30 min, then warmed to room temperature. Saturated aqueous NH_4Cl (2 mL) was added, the layers were separated, and the aqueous layer was extracted with Et_2O (3 × 10 mL). The combined organic layers were dried (Na_2SO_4) and evaporated under reduced pressure. Purification by column chromatography on silica gel, eluting with petrol−EtOAc (9:1), gave the nitrile **5f** (118 mg, 42%) as needles; m.p. 95−98 °C; R_f [petrol−EtOAc (4:1)] 0.31; FT-IR ν_{max} (film)/cm^{-1} 2980, 2865, 1710; ^1H NMR (400 MHz, $CDCl_3$) δ = 4.88 (1H, br s), 3.96 (1H, dt, *J* 14, 4.5), 3.47 (1H, ddd, *J* 14, 10.5, 3.5) 2.69−2.61 (1H, m), 2.46–2.41 (1H, m), 2.25−1.81 (8H, m), 1.75−1.63 (2H, m), 1.48 (9H, s); ^{13}C NMR (100 MHz, $CDCl_3$) δ = 154.2, 119.2, 82.0, 78.7, 67.6, 44.9, 34.5, 32.7, 29.4, 28.2, 23.9, 20.4, 14.0; HRMS (ES) Found MH^+ 281.1860. $C_{15}H_{25}N_2O_3$ requires MH^+, 281.1860. The enantiomers were resolved by chiral stationary phase GC (CSP-GC) using a Perkin Elmer Arnel Autosystem XL GC using an Astec CHIRALDEX beta cyclodextrin, permethylated fused silica capillary column (30 m x 0.25 mm) at 178 °C and 1.40 mL/min, retention times 20.7 and 21.1 min (see GC trace below).

Asymmetric method (general procedure C):

TMPMgCl (1.6 mL, 0.75 mmol) was added to carbamate **4** (54 mg, 0.25 mmol) in Et_2O (1 mL) at −104 °C. After 10 sec, cyclobutanone (0.056 mL, 0.75 mmol) was added. After 30 min, saturated aqueous NH_4Cl (0.3 mL) was added. The mixture was extracted with Et_2O (3 × 1 mL), dried ($MgSO_4$) and the solvent was evaporated. Purification by column chromatography on silica gel, eluting with petrol−EtOAc (9:1), gave the nitrile **5f** (47 mg, 68%); $[\alpha]^{21}_D$ –25.7 (0.4, $CHCl_3$); data as above. The enantiomer ratio was determined to be 95:5 by CSP-GC (major enantiomer eluted at 21.0 min and minor at 21.4 min).

***tert*-Butyl 2-(4-Bromobenzoyl)-2-cyanopiperidin-1-carboxylate 5g**

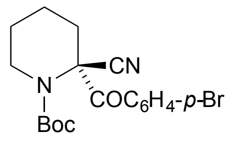

5g

Racemic method (general procedure A):

n-BuLi (0.44 mL, 1.1 mmol, 2.5 M in hexanes) was added dropwise to diisopropylamine (0.17 mL, 1.2 mmol) in THF (2 mL) at –25 °C under N_2. After 30 min, the mixture was cooled to –78 °C and the nitrile **4** (0.21 g, 1 mmol) was added. After 10 min, *p*-bromobenzoyl chloride (0.24 g, 1.1 mmol) was added. The resulting solution was stirred for 30 min, then warmed to room temperature. Saturated aqueous NH_4Cl (2 mL) was added, the layers were separated, and the aqueous layer was extracted with Et_2O (3 × 10 mL). The combined organic layers were dried (Na_2SO_4) and evaporated under reduced pressure. Purification by column chromatography on silica gel, eluting with petrol−EtOAc (9:1), gave the nitrile **5g** (333 mg, 85%) as a solid; m.p. 117−119 °C; R_f [petrol−EtOAc (4:1)] 0.30; FT-IR ν_{max} (film)/cm^{-1} 2980, 2155, 1720, 1690; ^{1}H NMR (400 MHz, $CDCl_3$) δ = 8.06 (2H, d, *J* 8), 7.61 (2H, d, *J* 8), 4.21 (1H, br d, *J* 12.5), 3.12 (1H, td, *J* 12.5, 2.5), 2.17−2.14 (2H, m), 2.07–1.97 (2H, m), 1.86−1.60 (2H, m), 1.26 (9H, s); ^{13}C NMR (100 MHz, $CDCl_3$, one aromatic C cannot be observed) δ = 188.8, 155.3, 131.8, 130.4, 128.4, 116.4, 84.1, 67.6, 43.6, 33.2, 27.7, 23.7, 20.3; HRMS (ES) Found MNa^+ 415.0629. $C_{18}H_{21}N_2O_3BrNa$ requires MNa^+, 415.0628. The enantiomers were resolved by chiral stationary phase HPLC using a cellulose 1 column with 1% ^{i}PrOH in hexanes at 1 mL/min, detection at 254 nm, retention times 59 and 64 min.

Asymmetric method (general procedure C):

TMPMgCl (1.6 mL, 0.75 mmol) was added to the nitrile **4** (54 mg, 0.25 mmol) in Et_2O (1 mL) at −104 °C. After 10 sec, *p*-bromobenzoyl chloride (165 mg, 0.75 mmol) in Et_2O (1 mL) was added at −104 °C. After 30 min, saturated aqueous NH_4Cl (0.3 mL) was added. The mixture was extracted with Et_2O (3 × 1 mL), dried ($MgSO_4$) and the solvent was evaporated. Purification by column chromatography on silica gel, eluting with petrol−EtOAc (9:1), gave the nitrile **5g** (71 mg, 72%); $[\alpha]^{21}_D$ –39.0 (0.4, $CHCl_3$); data as above. The enantiomer ratio was determined to be 83:17 by CSP-HPLC (major enantiomer eluted at 57 min and minor enantiomer at 65 min). Recrystallization gave er 99:1 by CSP HPLC. X-ray data deposited at CCDC 1477823.

Method from the sulfide **5c**

^{i}PrMgCl (0.375 mL, 0.75 mmol, 2 M in Et_2O) was added to the nitrile **5c** (87 mg, 0.25 mmol, er 85:15) in Et_2O (2 mL) at −104 °C. After 10 sec, *p*-bromobenzoyl chloride (165 mg, 0.75 mmol) in Et_2O (1 mL) was added at −104 °C. After 30 min, saturated aqueous NH_4Cl (0.3 mL) was added. The mixture was extracted with Et_2O (3 × 1 mL), dried ($MgSO_4$) and the solvent was evaporated. Purification by column chromatography on silica gel, eluting with petrol−EtOAc (9:1), gave the nitrile **5g** (32 mg, 33%); $[\alpha]^{21}_D$ –35.0 (0.4, $CHCl_3$); data as above. The enantiomer ratio was determined to be 78:22 by CSP-HPLC.

2. In situ IR spectra

A ReactIR probe was placed in THF/Et_2O (10 mL) at the required temperature and a background scan was run. After about 5 min, the nitrile **4** (1 mmol) was added in THF/Et_2O (2 mL). When the peak for the carbonyl group (at about 1705 cm^{-1}) had levelled out, TMPMgCl (1–3 molar equivalents) was added dropwise. After the metallated species had formed, the reaction was quenched by addition of MeOH or an electrophile.

At –78 °C the ReactIR traces are shown below. With 1 equiv. TMPMgCl, there is incomplete metallation. With two equiv. TMPMgCl, there is complete metallation within a few minutes.

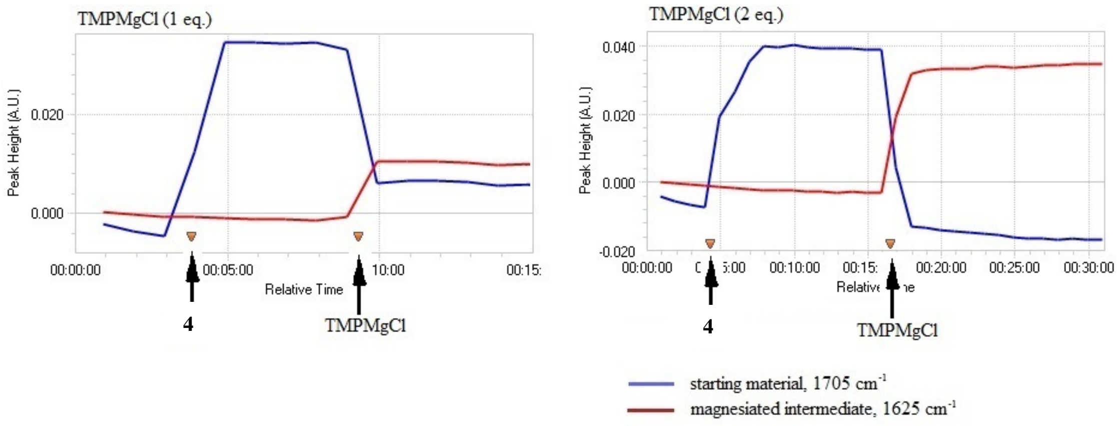

At –107 °C the ReactIR traces are shown below. With 1 equiv. TMPMgCl, there is incomplete metallation. With two equiv. TMPMgCl, there is almost complete metallation within a few minutes. With three equiv. TMPMgCl, there is complete metallation.

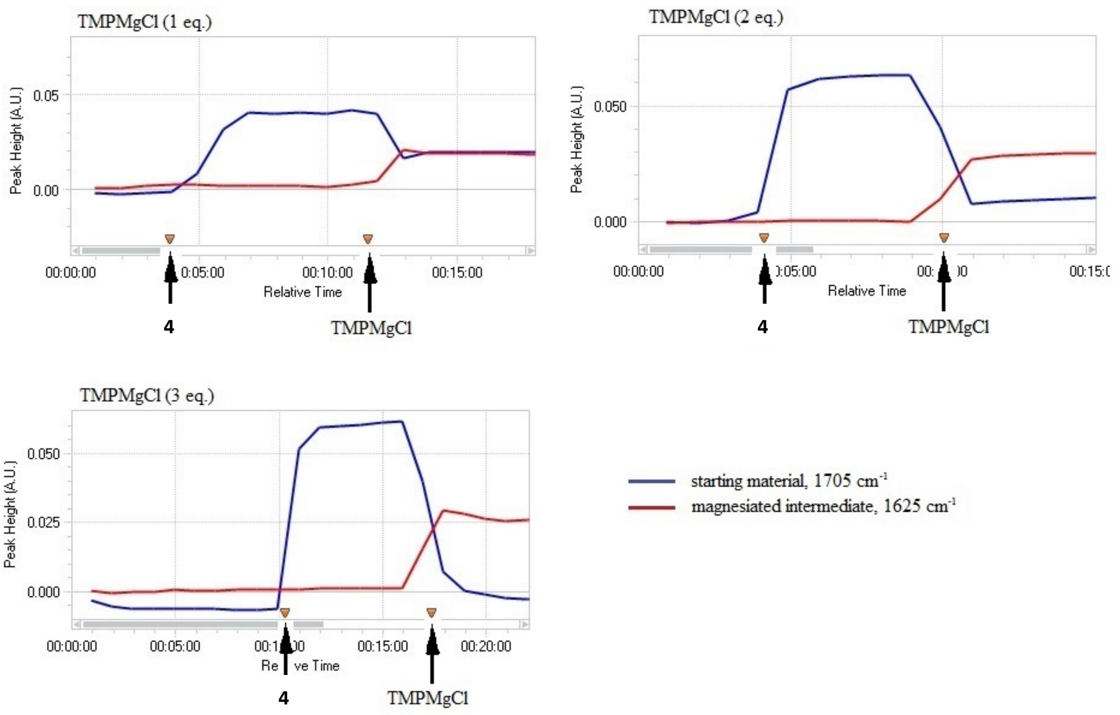

3. Kinetics of enantiomerization

(i) Kinetics of enantiomerization in Et_2O as solvent

TMPMgCl (1.6 mL, 0.75 mmol, 0.47 M in Et_2O) was added to the nitrile (*S*)-*N*-Boc-2-cyanopiperidine **4** (54 mg, 0.25 mmol, er >99:1) in Et_2O (1 mL) at –104 °C (total conc. 0.096 mM). The electrophile cyclobutanone (56 µL, 0.75 mmol) was added after various times as given below. The mixture was then quenched with aqueous NH_4Cl (0.6 mL) at –104 °C and was allowed to warm to room temperature. The mixture was extracted with Et_2O (5 mL) and the organic layer was evaporated. The enantiomer ratio was determined by using CSP-HPLC as described above.

Time	er (*S*:*R*) (of product **5f**)	
0 sec	100:0	(extrapolated for time zero)
10 sec	92:8	
20 sec	91:9	
30 sec	87:13	
60 sec	80:20	
90 sec	76:24	
120 sec	67:33	

First order plot of 0.5ln(ee at time t/ee at time 0) against time (sec):

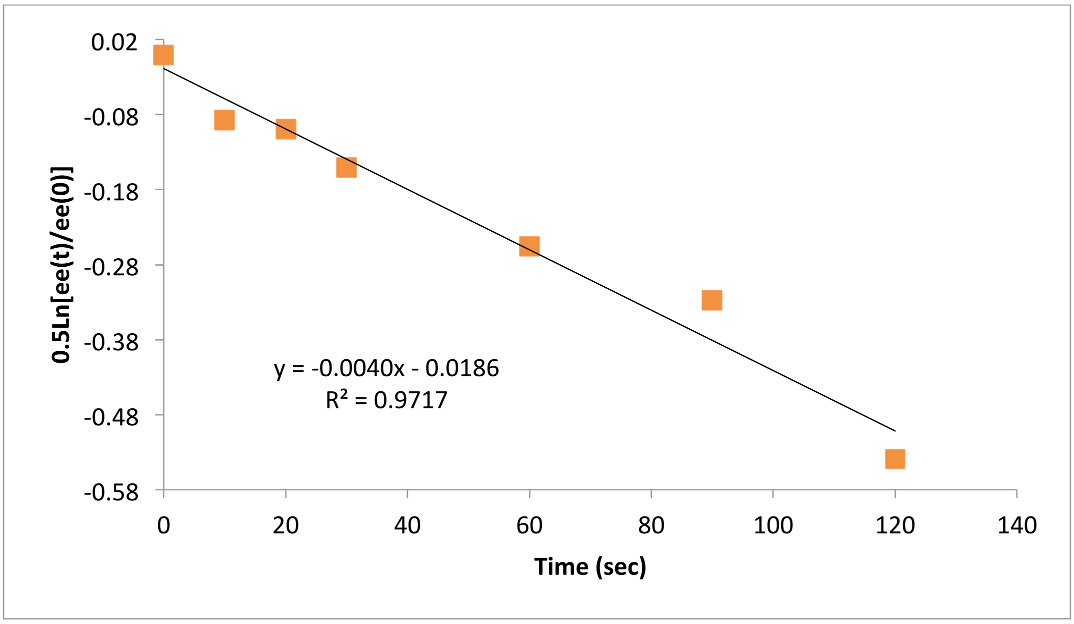

Gradient is –*k*(enantiomerization)

so enantiomerization (inversion) rate constant $k \approx 4 \times 10^{-3}\ s^{-1}$

Half-life for enantiomerization, $t_{1/2} \approx$ 173 sec (~2.9 min) at –104 °C

Barrier to enantiomerization, $\Delta G^{\ddagger} \approx$ 48.3 kJ/mol (11.6 kcal/mol) at –104 °C

(ii) Kinetics of enantiomerization in THF/Et_2O mixture as solvent

TMPMgCl (1.5 mL, 0.75 mmol, 0.5 M in THF) was added to the nitrile (*S*)-*N*-Boc-2-cyanopiperidine **4** (54 mg, 0.25 mmol, er >99:1) in Et_2O (1.1 mL) at –104 °C (total conc. 0.096 mM). The electrophile $PhSSO_2Ph$ (188 mg, 0.75 mmol) was added after various times as given below. The mixture was then quenched with aqueous NH_4Cl (0.6 mL) at –104 °C and was allowed to warm to room temperature. The mixture was extracted with Et_2O (5 mL) and the organic layer was evaporated. The enantiomer ratio was determined by using CSP-HPLC as described above.

Time	er (*R*:*S*) (of product **5a**)	
0 sec	100:0	(extrapolated, although *in situ* quench gave er 91:9)
10 sec	90:10	
30 sec	86:14	
60 sec	74:26	
90 sec	66:34	
120 sec	60:40	
150 sec	57:43	

First order plot of 0.5ln(ee at time t/ee at time 0) against time (sec):

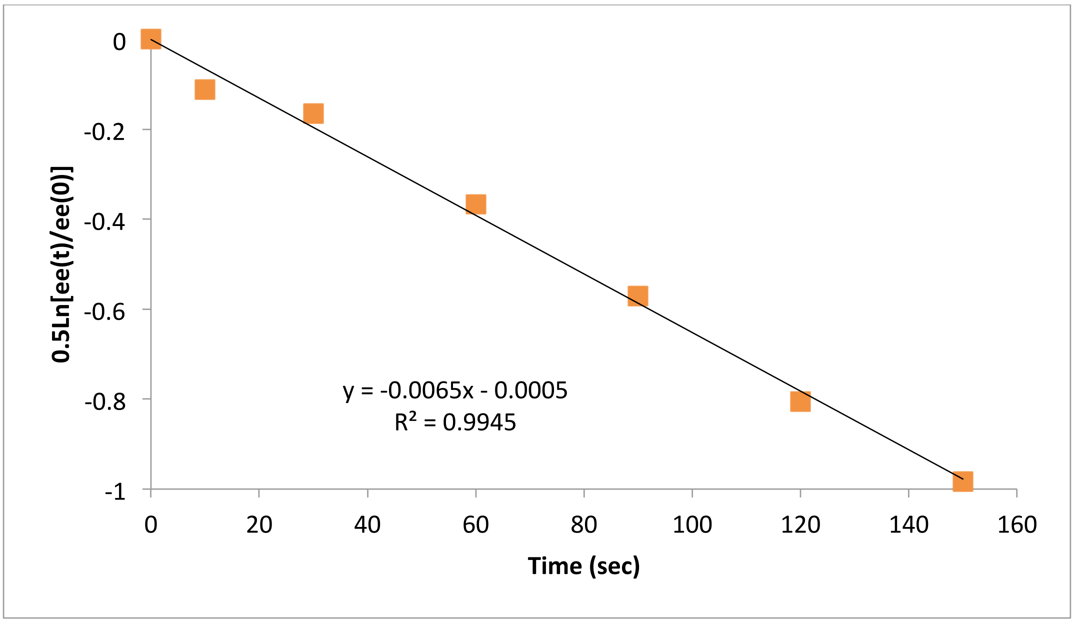

so enantiomerization (inversion) rate constant $k \approx 6.5 \times 10^{-3}\ s^{-1}$

Half-life for enantiomerization, $t_{1/2} \approx$ 107 sec (~1.8 min) at –104 °C

Barrier to enantiomerization, $\Delta G^{\ddagger} \approx$ 47.7 kJ/mol (11.4 kcal/mol) at –104 °C

4. X-ray data for compound 5g

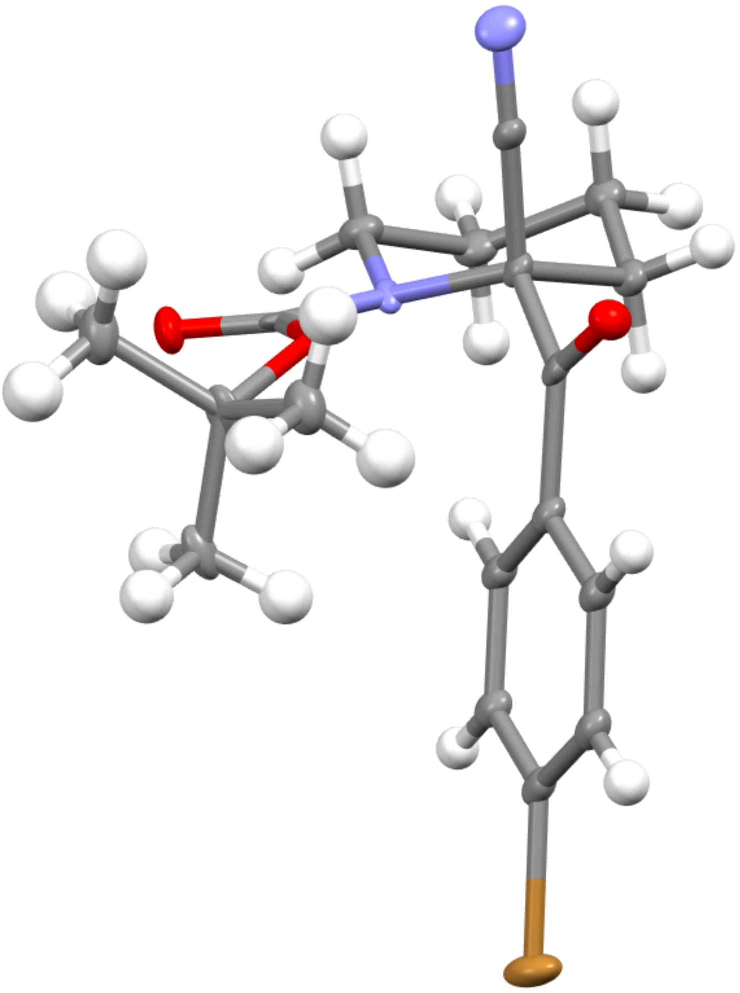

Table 1. Crystal data and structure refinement for compound **5g** (internal reference number: oic260redo_0m).

Identification code	shelx	
Empirical formula	C18 H21 Br N2 O3	
Formula weight	393.28	
Temperature	100(2) K	
Wavelength	1.54178 Å	
Crystal system	Monoclinic	
Space group	$P2_1$	
Unit cell dimensions	a = 8.8396(3) Å	a= 90°.
	b = 17.5572(7) Å	b= 94.347(2)°.
	c = 11.6494(4) Å	g = 90°.
Volume	1802.77(11) $Å^3$	
Z	4	
Density (calculated)	1.449 Mg/m^3	

Absorption coefficient	3.259 mm^{-1}
F(000)	808
Crystal size	0.120 x 0.120 x 0.100 mm^3
Theta range for data collection	3.805 to 67.017°.
Index ranges	-10<=h<=10, -20<=k<=20, -13<=l<=13
Reflections collected	44389
Independent reflections	44389 [R(int) = ?]
Completeness to theta = 67.000°	98.9 %
Absorption correction	Semi-empirical from equivalents
Max. and min. transmission	0.76 and 0.54
Refinement method	Full-matrix least-squares on F^2
Data / restraints / parameters	44389 / 261 / 440
Goodness-of-fit on F^2	1.022
Final R indices [I>2sigma(I)]	R1 = 0.0825, wR2 = 0.2026
R indices (all data)	R1 = 0.1283, wR2 = 0.2326
Absolute structure parameter	0.040(14)
Extinction coefficient	n/a
Largest diff. peak and hole	1.757 and -0.621 e.$Å^{-3}$

Table 2. Atomic coordinates (x 10^4) and equivalent isotropic displacement parameters ($Å^2$x 10^3) for **5g**. U(eq) is defined as one third of the trace of the orthogonalized U^{ij} tensor.

	x	y	z	U(eq)
Br(1)	3794(2)	2470(1)	4326(2)	27(1)
O(1)	3707(14)	5347(8)	426(10)	19(4)
O(2)	6813(15)	3466(8)	-1702(10)	19(3)
O(3)	4726(14)	3986(7)	-990(10)	17(3)
N(1)	7026(17)	4422(9)	-373(13)	13(4)
N(2)	5909(19)	6063(11)	-1548(14)	28(5)
C(1)	4120(20)	3200(12)	3175(16)	18(3)
C(2)	3070(20)	3743(12)	2947(15)	19(3)
C(3)	3280(20)	4296(12)	2129(15)	20(3)
C(4)	4620(20)	4282(12)	1521(15)	18(3)
C(5)	5690(20)	3726(11)	1777(15)	18(3)
C(6)	5440(20)	3162(12)	2601(15)	18(3)
C(7)	4750(20)	4913(12)	642(15)	18(3)

C(8)	6260(20)	5071(12)	132(16)	18(3)
C(9)	7310(20)	5403(12)	1137(14)	18(3)
C(10)	8910(20)	5604(12)	809(16)	21(3)
C(11)	9600(20)	4870(12)	346(15)	21(3)
C(12)	8630(20)	4570(12)	-668(15)	20(4)
C(13)	6230(20)	3914(11)	-1102(16)	16(4)
C(14)	3620(20)	3378(11)	-1457(16)	17(3)
C(15)	3630(20)	3340(12)	-2750(15)	19(4)
C(16)	2160(20)	3692(12)	-1096(16)	24(4)
C(17)	3956(19)	2597(12)	-904(14)	20(4)
C(18)	6000(20)	5646(12)	-790(16)	18(3)
Br(00)	11167(2)	7519(1)	9656(2)	28(1)
O(1A)	11572(15)	4752(8)	5559(11)	19(3)
O(2A)	7898(15)	6682(8)	3624(10)	21(3)
O(3A)	10154(14)	6110(7)	4158(10)	14(3)
N(1A)	7980(17)	5588(9)	4674(12)	12(4)
N(2A)	9310(19)	4112(11)	3273(15)	21(4)
C(1A)	10910(20)	6796(12)	8464(15)	17(3)
C(2A)	9490(20)	6686(11)	7889(14)	17(3)
C(3A)	9290(20)	6146(11)	7020(15)	17(3)
C(4A)	10490(20)	5734(12)	6717(17)	16(3)
C(5A)	11970(20)	5834(12)	7299(15)	18(3)
C(6A)	12150(20)	6366(12)	8185(15)	19(3)
C(7A)	10450(20)	5149(12)	5763(15)	15(3)
C(8A)	8930(20)	4930(11)	5098(15)	15(3)
C(9A)	8130(20)	4450(11)	5957(15)	18(3)
C(10A)	6520(20)	4234(12)	5514(15)	20(3)
C(11A)	5620(20)	4980(12)	5251(15)	20(3)
C(12A)	6350(20)	5441(12)	4337(15)	20(4)
C(13A)	8650(20)	6176(11)	4093(16)	15(4)
C(14A)	11130(20)	6795(11)	3964(17)	20(4)
C(15A)	12690(20)	6445(12)	4168(17)	26(4)
C(16A)	10949(19)	7420(14)	4840(14)	21(4)
C(17A)	10900(20)	7056(13)	2740(16)	27(4)
C(18A)	9220(20)	4464(11)	4069(16)	14(3)

Table 3. Bond lengths [Å] and angles [°] for **5g**.

Br(1)-C(1)	1.891(19)
O(1)-C(7)	1.21(2)
O(2)-C(13)	1.20(2)
O(3)-C(13)	1.35(2)
O(3)-C(14)	1.52(2)
N(1)-C(13)	1.39(2)
N(1)-C(8)	1.47(2)
N(1)-C(12)	1.50(2)
N(2)-C(18)	1.14(2)
C(1)-C(2)	1.34(3)
C(1)-C(6)	1.39(2)
C(2)-C(3)	1.38(3)
C(2)-H(2)	0.9500
C(3)-C(4)	1.42(3)
C(3)-H(3)	0.9500
C(4)-C(5)	1.38(3)
C(4)-C(7)	1.52(3)
C(5)-C(6)	1.41(3)
C(5)-H(5)	0.9500
C(6)-H(6)	0.9500
C(7)-C(8)	1.53(3)
C(8)-C(18)	1.48(3)
C(8)-C(9)	1.55(3)
C(9)-C(10)	1.54(2)
C(9)-H(9A)	0.9900
C(9)-H(9B)	0.9900
C(10)-C(11)	1.54(3)
C(10)-H(10A)	0.9900
C(10)-H(10B)	0.9900
C(11)-C(12)	1.50(3)
C(11)-H(11A)	0.9900
C(11)-H(11B)	0.9900
C(12)-H(12A)	0.9900
C(12)-H(12B)	0.9900
C(14)-C(15)	1.51(2)
C(14)-C(16)	1.49(3)

C(14)-C(17)	1.54(3)
C(15)-H(15A)	0.9800
C(15)-H(15B)	0.9800
C(15)-H(15C)	0.9800
C(16)-H(16A)	0.9800
C(16)-H(16B)	0.9800
C(16)-H(16C)	0.9800
C(17)-H(17A)	0.9800
C(17)-H(17B)	0.9800
C(17)-H(17C)	0.9800
Br(00)-C(1A)	1.882(19)
O(1A)-C(7A)	1.25(2)
O(2A)-C(13A)	1.21(2)
O(3A)-C(13A)	1.33(2)
O(3A)-C(14A)	1.50(2)
N(1A)-C(13A)	1.39(2)
N(1A)-C(8A)	1.49(2)
N(1A)-C(12A)	1.49(2)
N(2A)-C(18A)	1.12(2)
C(1A)-C(6A)	1.38(3)
C(1A)-C(2A)	1.40(2)
C(2A)-C(3A)	1.39(3)
C(2A)-H(2A)	0.9500
C(3A)-C(4A)	1.35(3)
C(3A)-H(3A)	0.9500
C(4A)-C(5A)	1.44(3)
C(4A)-C(7A)	1.51(3)
C(5A)-C(6A)	1.39(3)
C(5A)-H(5A)	0.9500
C(6A)-H(6A)	0.9500
C(7A)-C(8A)	1.55(3)
C(8A)-C(18A)	1.49(3)
C(8A)-C(9A)	1.52(2)
C(9A)-C(10A)	1.52(3)
C(9A)-H(9A1)	0.9900
C(9A)-H(9A2)	0.9900
C(10A)-C(11A)	1.55(3)
C(10A)-H(10C)	0.9900

C(10A)-H(10D)	0.9900
C(11A)-C(12A)	1.52(3)
C(11A)-H(11C)	0.9900
C(11A)-H(11D)	0.9900
C(12A)-H(12C)	0.9900
C(12A)-H(12D)	0.9900
C(14A)-C(17A)	1.50(2)
C(14A)-C(15A)	1.52(3)
C(14A)-C(16A)	1.52(3)
C(15A)-H(15D)	0.9800
C(15A)-H(15E)	0.9800
C(15A)-H(15F)	0.9800
C(16A)-H(16D)	0.9800
C(16A)-H(16E)	0.9800
C(16A)-H(16F)	0.9800
C(17A)-H(17D)	0.9800
C(17A)-H(17E)	0.9800
C(17A)-H(17F)	0.9800

C(13)-O(3)-C(14)	120.8(14)
C(13)-N(1)-C(8)	121.0(15)
C(13)-N(1)-C(12)	114.4(15)
C(8)-N(1)-C(12)	115.4(15)
C(2)-C(1)-C(6)	122.0(18)
C(2)-C(1)-Br(1)	118.9(15)
C(6)-C(1)-Br(1)	119.1(15)
C(1)-C(2)-C(3)	120.5(19)
C(1)-C(2)-H(2)	119.7
C(3)-C(2)-H(2)	119.7
C(2)-C(3)-C(4)	119(2)
C(2)-C(3)-H(3)	120.3
C(4)-C(3)-H(3)	120.3
C(5)-C(4)-C(3)	119.3(18)
C(5)-C(4)-C(7)	125.3(18)
C(3)-C(4)-C(7)	115.4(18)
C(4)-C(5)-C(6)	120.1(19)
C(4)-C(5)-H(5)	119.9
C(6)-C(5)-H(5)	119.9

C(1)-C(6)-C(5)	118.6(19)
C(1)-C(6)-H(6)	120.7
C(5)-C(6)-H(6)	120.7
O(1)-C(7)-C(4)	120.6(17)
O(1)-C(7)-C(8)	118.5(18)
C(4)-C(7)-C(8)	120.4(17)
N(1)-C(8)-C(18)	106.8(15)
N(1)-C(8)-C(7)	117.3(17)
C(18)-C(8)-C(7)	108.4(16)
N(1)-C(8)-C(9)	108.9(15)
C(18)-C(8)-C(9)	110.0(17)
C(7)-C(8)-C(9)	105.4(15)
C(10)-C(9)-C(8)	114.2(14)
C(10)-C(9)-H(9A)	108.7
C(8)-C(9)-H(9A)	108.7
C(10)-C(9)-H(9B)	108.7
C(8)-C(9)-H(9B)	108.7
H(9A)-C(9)-H(9B)	107.6
C(9)-C(10)-C(11)	106.8(16)
C(9)-C(10)-H(10A)	110.4
C(11)-C(10)-H(10A)	110.4
C(9)-C(10)-H(10B)	110.4
C(11)-C(10)-H(10B)	110.4
H(10A)-C(10)-H(10B)	108.6
C(12)-C(11)-C(10)	110.7(17)
C(12)-C(11)-H(11A)	109.5
C(10)-C(11)-H(11A)	109.5
C(12)-C(11)-H(11B)	109.5
C(10)-C(11)-H(11B)	109.5
H(11A)-C(11)-H(11B)	108.1
C(11)-C(12)-N(1)	111.8(14)
C(11)-C(12)-H(12A)	109.3
N(1)-C(12)-H(12A)	109.3
C(11)-C(12)-H(12B)	109.3
N(1)-C(12)-H(12B)	109.3
H(12A)-C(12)-H(12B)	107.9
O(2)-C(13)-O(3)	126.0(18)
O(2)-C(13)-N(1)	124.2(18)

O(3)-C(13)-N(1)	109.8(16)
C(15)-C(14)-O(3)	109.6(13)
C(15)-C(14)-C(16)	111.6(16)
O(3)-C(14)-C(16)	100.8(15)
C(15)-C(14)-C(17)	111.3(16)
O(3)-C(14)-C(17)	112.2(15)
C(16)-C(14)-C(17)	110.8(15)
C(14)-C(15)-H(15A)	109.5
C(14)-C(15)-H(15B)	109.5
H(15A)-C(15)-H(15B)	109.5
C(14)-C(15)-H(15C)	109.5
H(15A)-C(15)-H(15C)	109.5
H(15B)-C(15)-H(15C)	109.5
C(14)-C(16)-H(16A)	109.5
C(14)-C(16)-H(16B)	109.5
H(16A)-C(16)-H(16B)	109.5
C(14)-C(16)-H(16C)	109.5
H(16A)-C(16)-H(16C)	109.5
H(16B)-C(16)-H(16C)	109.5
C(14)-C(17)-H(17A)	109.5
C(14)-C(17)-H(17B)	109.5
H(17A)-C(17)-H(17B)	109.5
C(14)-C(17)-H(17C)	109.5
H(17A)-C(17)-H(17C)	109.5
H(17B)-C(17)-H(17C)	109.5
N(2)-C(18)-C(8)	174(2)
C(13A)-O(3A)-C(14A)	120.1(14)
C(13A)-N(1A)-C(8A)	119.2(15)
C(13A)-N(1A)-C(12A)	116.1(15)
C(8A)-N(1A)-C(12A)	117.6(16)
C(6A)-C(1A)-C(2A)	120.7(19)
C(6A)-C(1A)-Br(00)	119.3(14)
C(2A)-C(1A)-Br(00)	120.0(15)
C(3A)-C(2A)-C(1A)	120.1(19)
C(3A)-C(2A)-H(2A)	120.0
C(1A)-C(2A)-H(2A)	120.0
C(4A)-C(3A)-C(2A)	119.9(19)
C(4A)-C(3A)-H(3A)	120.0

C(2A)-C(3A)-H(3A)	120.0
C(3A)-C(4A)-C(5A)	121.0(19)
C(3A)-C(4A)-C(7A)	125.4(19)
C(5A)-C(4A)-C(7A)	113.6(18)
C(6A)-C(5A)-C(4A)	118.6(19)
C(6A)-C(5A)-H(5A)	120.7
C(4A)-C(5A)-H(5A)	120.7
C(5A)-C(6A)-C(1A)	119.7(18)
C(5A)-C(6A)-H(6A)	120.2
C(1A)-C(6A)-H(6A)	120.2
O(1A)-C(7A)-C(4A)	123.1(18)
O(1A)-C(7A)-C(8A)	115.9(18)
C(4A)-C(7A)-C(8A)	120.5(17)
N(1A)-C(8A)-C(18A)	106.6(14)
N(1A)-C(8A)-C(9A)	111.5(15)
C(18A)-C(8A)-C(9A)	110.1(16)
N(1A)-C(8A)-C(7A)	114.8(16)
C(18A)-C(8A)-C(7A)	110.0(16)
C(9A)-C(8A)-C(7A)	103.9(14)
C(8A)-C(9A)-C(10A)	112.6(15)
C(8A)-C(9A)-H(9A1)	109.1
C(10A)-C(9A)-H(9A1)	109.1
C(8A)-C(9A)-H(9A2)	109.1
C(10A)-C(9A)-H(9A2)	109.1
H(9A1)-C(9A)-H(9A2)	107.8
C(9A)-C(10A)-C(11A)	108.1(17)
C(9A)-C(10A)-H(10C)	110.1
C(11A)-C(10A)-H(10C)	110.1
C(9A)-C(10A)-H(10D)	110.1
C(11A)-C(10A)-H(10D)	110.1
H(10C)-C(10A)-H(10D)	108.4
C(12A)-C(11A)-C(10A)	110.3(16)
C(12A)-C(11A)-H(11C)	109.6
C(10A)-C(11A)-H(11C)	109.6
C(12A)-C(11A)-H(11D)	109.6
C(10A)-C(11A)-H(11D)	109.6
H(11C)-C(11A)-H(11D)	108.1
N(1A)-C(12A)-C(11A)	111.3(15)

N(1A)-C(12A)-H(12C)	109.4
C(11A)-C(12A)-H(12C)	109.4
N(1A)-C(12A)-H(12D)	109.4
C(11A)-C(12A)-H(12D)	109.4
H(12C)-C(12A)-H(12D)	108.0
O(2A)-C(13A)-O(3A)	126.8(18)
O(2A)-C(13A)-N(1A)	121.6(18)
O(3A)-C(13A)-N(1A)	111.6(16)
C(17A)-C(14A)-O(3A)	110.6(15)
C(17A)-C(14A)-C(15A)	109.1(17)
O(3A)-C(14A)-C(15A)	100.3(15)
C(17A)-C(14A)-C(16A)	114.0(18)
O(3A)-C(14A)-C(16A)	112.9(15)
C(15A)-C(14A)-C(16A)	109.1(16)
C(14A)-C(15A)-H(15D)	109.5
C(14A)-C(15A)-H(15E)	109.5
H(15D)-C(15A)-H(15E)	109.5
C(14A)-C(15A)-H(15F)	109.5
H(15D)-C(15A)-H(15F)	109.5
H(15E)-C(15A)-H(15F)	109.5
C(14A)-C(16A)-H(16D)	109.5
C(14A)-C(16A)-H(16E)	109.5
H(16D)-C(16A)-H(16E)	109.5
C(14A)-C(16A)-H(16F)	109.5
H(16D)-C(16A)-H(16F)	109.5
H(16E)-C(16A)-H(16F)	109.5
C(14A)-C(17A)-H(17D)	109.5
C(14A)-C(17A)-H(17E)	109.5
H(17D)-C(17A)-H(17E)	109.5
C(14A)-C(17A)-H(17F)	109.5
H(17D)-C(17A)-H(17F)	109.5
H(17E)-C(17A)-H(17F)	109.5
N(2A)-C(18A)-C(8A)	174(2)

Symmetry transformations used to generate equivalent atoms:

Table 4. Anisotropic displacement parameters ($Å^2$x 10^3) for **5g**. The anisotropic displacement factor exponent takes the form: $-2p^2[h^2 a^{*2}U^{11} + ... + 2 h k a^* b^* U^{12}]$

	U^{11}	U^{22}	U^{33}	U^{23}	U^{13}	U^{12}
Br(1)	34(1)	31(1)	15(1)	9(1)	1(1)	-10(1)
O(1)	18(9)	23(9)	15(8)	4(6)	5(7)	3(7)
O(2)	27(8)	18(8)	13(7)	-5(6)	2(6)	3(6)
O(3)	25(8)	13(8)	14(7)	-4(5)	5(6)	-10(6)
N(1)	12(9)	12(9)	16(9)	-4(7)	-3(7)	1(7)
N(2)	15(10)	39(13)	30(11)	12(9)	4(8)	3(9)
C(1)	21(6)	22(7)	11(6)	2(5)	0(5)	-6(5)
C(2)	20(6)	25(7)	13(6)	3(5)	1(5)	-6(5)
C(3)	20(6)	25(7)	14(6)	2(5)	2(5)	-3(5)
C(4)	20(6)	23(6)	10(5)	-1(4)	2(5)	-1(5)
C(5)	24(6)	22(7)	10(6)	0(5)	2(5)	-1(5)
C(6)	23(6)	22(7)	10(6)	-1(5)	0(5)	-2(5)
C(7)	23(6)	21(6)	10(6)	-2(5)	4(5)	2(5)
C(8)	22(6)	22(6)	10(5)	-1(5)	5(5)	3(5)
C(9)	24(6)	22(7)	10(6)	-2(5)	5(5)	2(5)
C(10)	26(6)	26(7)	12(6)	-3(5)	4(5)	-2(6)
C(11)	24(7)	28(7)	13(7)	-3(6)	2(5)	1(6)
C(12)	23(8)	25(8)	12(7)	-2(6)	5(6)	1(7)
C(13)	17(12)	18(12)	12(11)	10(9)	5(9)	9(9)
C(14)	19(8)	14(8)	18(7)	-1(6)	6(7)	-11(7)
C(15)	20(10)	20(10)	18(8)	-1(7)	4(8)	-13(8)
C(16)	26(9)	20(10)	25(10)	-5(8)	6(8)	-5(8)
C(17)	10(8)	15(10)	37(9)	9(8)	12(7)	-2(8)
C(18)	19(8)	22(8)	13(7)	2(6)	4(6)	3(7)
Br(00)	36(1)	32(1)	17(1)	-10(1)	4(1)	-11(1)
O(1A)	16(8)	19(9)	21(8)	-2(6)	0(6)	3(7)
O(2A)	28(9)	14(8)	22(8)	6(6)	1(6)	5(6)
O(3A)	18(8)	12(7)	11(7)	2(5)	1(6)	-1(6)
N(1A)	16(10)	10(9)	11(8)	1(6)	6(7)	5(7)
N(2A)	12(10)	30(12)	21(10)	-3(8)	6(8)	4(8)
C(1A)	19(6)	20(7)	11(6)	4(5)	-1(5)	-6(5)
C(2A)	19(6)	20(7)	12(6)	2(5)	1(5)	-4(5)
C(3A)	20(6)	19(6)	11(6)	2(5)	0(5)	-3(5)

C(4A)	19(5)	19(6)	10(5)	3(4)	-1(5)	-2(5)
C(5A)	19(6)	21(7)	13(6)	3(5)	-1(5)	-1(5)
C(6A)	22(6)	22(7)	14(6)	4(5)	-3(5)	-3(5)
C(7A)	20(6)	18(6)	8(6)	4(5)	-2(5)	-2(5)
C(8A)	19(6)	16(6)	9(6)	3(5)	0(5)	-2(5)
C(9A)	25(6)	17(6)	12(6)	2(5)	3(5)	-4(5)
C(10A)	24(6)	21(7)	14(6)	0(5)	3(6)	-3(5)
C(11A)	24(7)	24(7)	14(7)	-3(6)	4(6)	-1(6)
C(12A)	22(8)	23(8)	14(8)	-3(6)	0(7)	-4(7)
C(13A)	18(12)	14(11)	11(11)	-8(8)	-5(9)	1(9)
C(14A)	15(7)	15(8)	29(8)	8(6)	4(7)	-7(6)
C(15A)	24(9)	23(10)	30(10)	7(8)	6(8)	0(8)
C(16A)	12(8)	13(9)	39(9)	6(8)	2(7)	2(9)
C(17A)	21(10)	27(11)	32(9)	12(8)	5(8)	1(9)
C(18A)	16(7)	15(8)	11(7)	1(6)	-2(6)	-2(6)

Table 5. Hydrogen coordinates (x 10^4) and isotropic displacement parameters (Å^{2}x 10 ^{3}) for **5g**.

	x	y	z	U(eq)
H(2)	2176	3749	3351	23
H(3)	2544	4683	1974	23
H(5)	6605	3723	1397	22
H(6)	6151	2765	2761	22
H(9A)	6830	5868	1427	22
H(9B)	7391	5028	1772	22
H(10A)	9540	5791	1491	25
H(10B)	8865	6007	212	25
H(11A)	10630	4976	113	26
H(11B)	9679	4480	962	26
H(12A)	9073	4090	-937	24
H(12B)	8616	4943	-1305	24
H(15A)	2825	2996	-3057	29

H(15B)	4615	3149	-2956	29
H(15C)	3456	3850	-3076	29
H(16A)	1981	4197	-1437	36
H(16B)	2209	3733	-255	36
H(16C)	1324	3351	-1358	36
H(17A)	3802	2622	-80	30
H(17B)	5009	2454	-1005	30
H(17C)	3271	2215	-1272	30
H(2A)	8646	6981	8093	20
H(3A)	8316	6065	6639	20
H(5A)	12812	5544	7084	21
H(6A)	13106	6433	8598	23
H(9A1)	8089	4739	6684	22
H(9A2)	8719	3980	6128	22
H(10C)	6031	3932	6101	24
H(10D)	6541	3923	4806	24
H(11C)	4557	4855	4978	24
H(11D)	5592	5286	5963	24
H(12C)	6253	5160	3598	24
H(12D)	5809	5932	4222	24
H(15D)	13463	6823	4002	38
H(15E)	12775	6003	3662	38
H(15F)	12856	6282	4973	38
H(16D)	9907	7614	4761	32
H(16E)	11656	7836	4707	32
H(16F)	11170	7216	5617	32
H(17D)	9816	7063	2498	40
H(17E)	11423	6706	2246	40
H(17F)	11322	7569	2674	40

Table 6. Hydrogen bonds for **5g** [Å and °].

D-H...A	d(D-H)	d(H...A)	d(D...A)	<(DHA)
C(17A)-H(17F)...O(2)#1	0.98	2.60	3.47(2)	147.9
C(17A)-H(17D)...O(2A)	0.98	2.32	2.99(2)	125.2
C(16A)-H(16D)...O(2A)	0.98	2.69	3.22(2)	114.6
C(6A)-H(6A)...N(2)#2	0.95	2.58	3.36(3)	139.6
C(17)-H(17B)...O(2)	0.98	2.56	3.15(2)	118.8
C(15)-H(15B)...O(2)	0.98	2.41	2.99(2)	117.6
C(15)-H(15A)...O(2A)#3	0.98	2.47	3.34(2)	147.0
C(2)-H(2)...N(2A)#4	0.95	2.61	3.44(3)	145.9

Symmetry transformations used to generate equivalent atoms:

#1 -x+2,y+1/2,-z #2 x+1,y,z+1 #3 -x+1,y-1/2,-z

#4 x-1,y,z

5. DFT data

All calculations were performed as described in the main text of the paper. Optimizations were started from a number of chemically feasible magnesiated nitrile structures. It is noted that in particular for the non-bridged structures, the lability of coordinated diethylether means that a number of isomers are possible. As far as we can ascertain all these structures have been generated, although only the lowest energy structures are reported here. After convergence, frequencies were calculated in the harmonic approximation to confirm whether a minimum was found through the absence of imaginary frequencies.

4.1 1st rotamer of 4

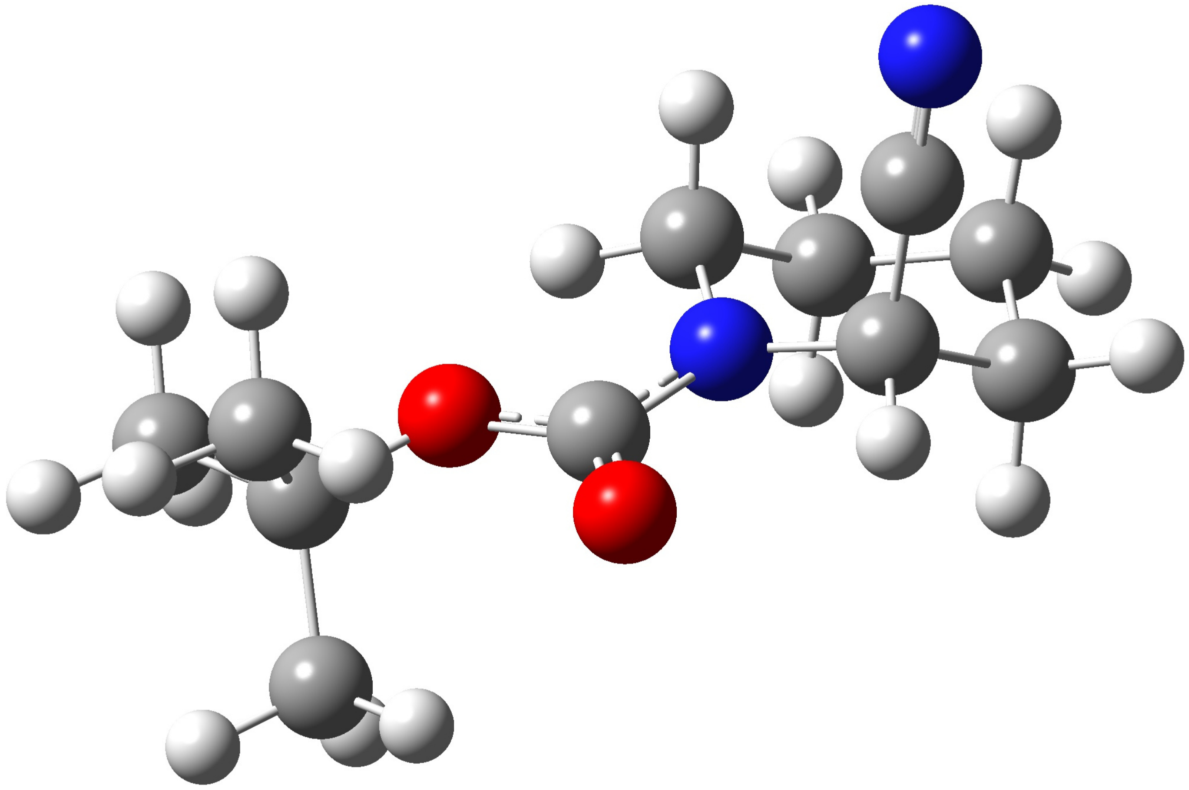

Figure S1: 1st rotamer of *4*

Route	: # opt freq b3lyp/6-311g(d,p) scrf=(solvent=diethylether) geom=connectivity empiricaldispersion=gd3bj int=ultrafine pop=(regular,mk)	
SMILES	: CC(C)(C)OC(=O)N1CCCCC1C#N	
Formula	: $C_{11}H_{18}N_2O_2$	
Charge	: 0	
Multiplicity	: 1	
Dipole	: 13.6600	Debye
Energy	: -690.23731225	a.u.
Gibbs Energy	: -689.99801600	a.u.

4.1.1 Cartesian Co-ordinates (XYZ format)

33

C	-2.08972311	-1.44285202	-1.08634698
C	-1.75053704	0.99568301	-0.71651202
C	-2.34531808	0.87544602	0.69872200
C	-2.14887094	-0.53078002	1.26988399
C	-2.70493293	-1.58412504	0.30634999
H	-2.56423092	-2.11134791	-1.79660201
H	-1.02045095	-1.69014704	-1.05313802
H	-1.89509296	1.63407195	1.34240103
H	-3.41183400	1.10103202	0.61249900
H	-1.08098698	-0.71238798	1.43694401
H	-2.63851595	-0.60539901	2.24356103
H	-2.50560999	-2.59157300	0.68070501
H	-3.79147696	-1.47238803	0.22578099
N	-2.24924397	-0.06904600	-1.57936502
C	-2.58337092	0.26471099	-2.87026095
O	-2.58636189	1.41127801	-3.28364706
O	-2.91705799	-0.82396001	-3.58109593
C	-3.34819293	-0.71490300	-4.99208689
C	-4.62627077	0.11646300	-5.08197880
C	-2.21279597	-0.14024501	-5.83655596
C	-3.61893511	-2.16986489	-5.36301088
H	-5.38872290	-0.29214299	-4.41445780
H	-4.43861294	1.15449703	-4.81553316
H	-5.01033878	0.07714500	-6.10405111
H	-1.30489898	-0.73219901	-5.69916821
H	-2.49228501	-0.18165600	-6.89201880
H	-2.00754309	0.89324200	-5.56568813
H	-3.94472098	-2.23221803	-6.40321398
H	-2.71414399	-2.76934910	-5.24456596
H	-4.40165615	-2.58831596	-4.72697401
C	-0.27324501	0.96360302	-0.67479402
N	0.87763798	0.91216701	-0.63615602
H	-2.03997898	1.94199097	-1.17207694

4.1.2 Frequencies

**Mode**	**IR frequency**	**IR intensity**
1	13.62160000	1.48590000
2	37.79710000	1.29040000
3	67.47100000	4.82960000
4	104.75250000	3.16540000
5	114.95770000	1.45210000
6	125.10550000	1.38750000
7	140.73230000	3.24330000
8	204.55000000	0.19430000
9	228.64720000	6.77090000
10	242.69000000	1.15190000
11	252.84830000	0.51900000

12	270.50240000	0.02010000
13	278.40110000	2.19320000
14	304.31950000	2.76450000
15	346.20480000	25.88200000
16	350.80720000	2.66320000
17	373.14210000	2.72460000
18	400.69800000	6.40600000
19	416.53700000	1.58520000
20	440.22450000	4.00210000
21	458.49780000	3.37910000
22	491.53890000	4.32330000
23	517.66110000	9.58440000
24	602.60090000	3.06130000
25	624.47610000	3.83390000
26	695.04060000	1.19450000
27	770.51140000	6.35880000
28	774.50680000	30.49840000
29	807.58290000	0.86900000
30	825.87490000	12.46580000
31	852.86060000	7.71370000
32	865.45460000	53.80030000
33	884.54060000	40.73970000
34	928.91820000	0.06510000
35	930.29350000	3.98860000
36	934.30930000	27.55310000
37	945.95460000	9.42240000
38	971.62100000	0.18040000
39	1005.46270000	17.77180000
40	1045.39900000	83.43710000
41	1052.65260000	0.58610000
42	1057.37290000	0.49920000
43	1082.94800000	13.25010000
44	1108.38720000	55.83840000
45	1152.11930000	67.00980000
46	1156.22430000	52.03160000
47	1180.70690000	514.64460000
48	1203.02730000	16.01840000
49	1269.95730000	18.97130000
50	1270.59620000	25.94150000
51	1277.33770000	163.26600000
52	1291.67330000	35.33420000
53	1294.72960000	137.23670000
54	1310.98440000	15.64560000
55	1355.18360000	80.63720000
56	1367.73940000	43.50500000
57	1385.58010000	14.64860000
58	1387.06260000	8.36380000
59	1396.07430000	29.07300000
60	1397.72160000	43.49210000
61	1404.30450000	56.53710000
62	1422.49850000	31.57030000
63	1434.06640000	366.81770000
64	1467.08140000	0.46000000
65	1475.77570000	5.42880000
66	1483.88940000	12.30480000

67	1486.17860000	2.69840000
68	1486.94450000	0.07990000
69	1490.23770000	22.93750000
70	1493.05910000	3.44540000
71	1500.46530000	1.89830000
72	1507.90510000	5.84890000
73	1521.49850000	29.00810000
74	1730.28420000	540.88630000
75	2346.12740000	10.54030000
76	2998.36460000	37.60960000
77	3022.05230000	16.28150000
78	3029.20420000	48.88850000
79	3037.14560000	13.20100000
80	3039.36760000	34.52200000
81	3045.88510000	27.26000000
82	3046.01060000	16.98710000
83	3075.20590000	51.27490000
84	3078.83930000	59.92000000
85	3094.20740000	37.04230000
86	3101.00430000	10.00840000
87	3102.63200000	21.80280000
88	3108.53420000	12.62730000
89	3110.44670000	44.45730000
90	3115.35120000	61.82630000
91	3144.21240000	1.15330000
92	3147.24600000	22.11370000
93	3166.25310000	4.96030000

4.2 2nd rotamer of 4

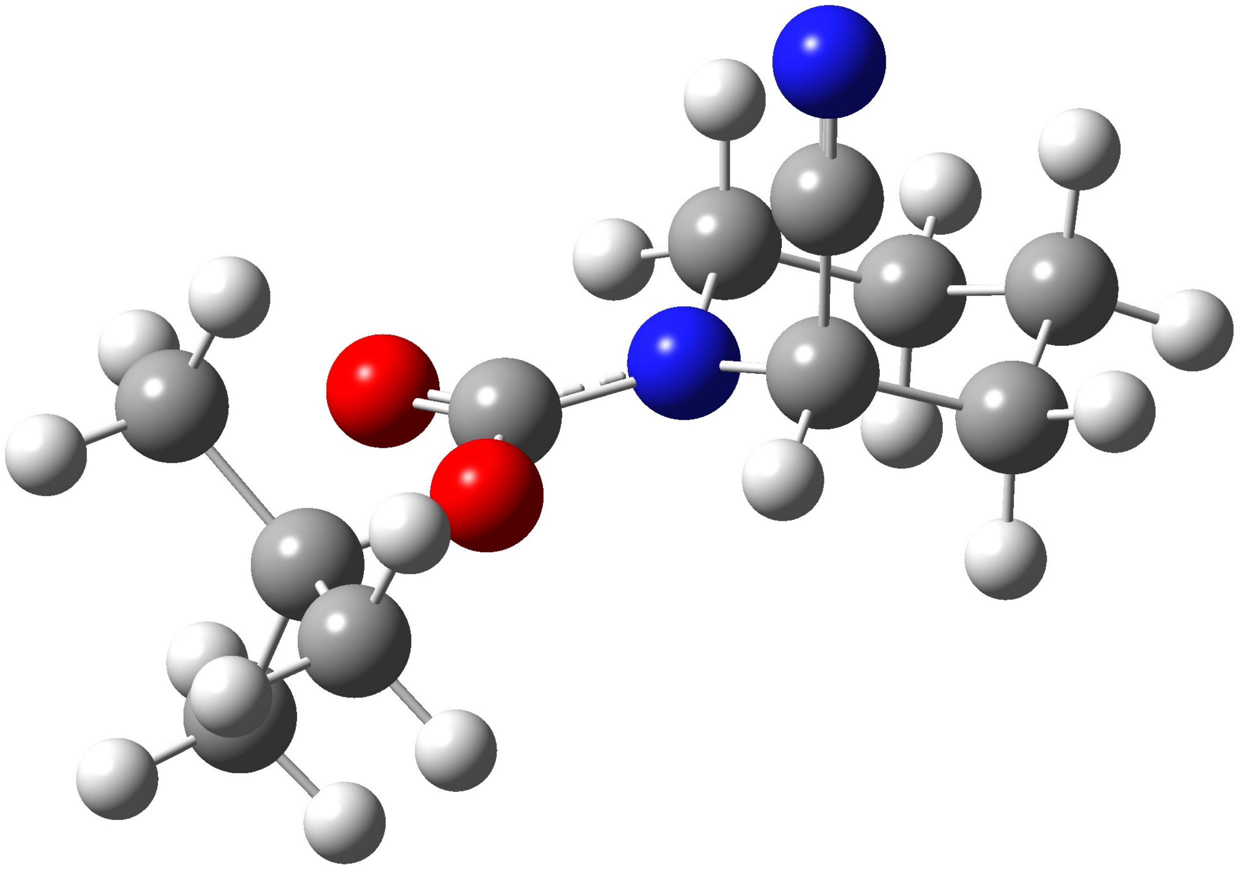

Figure S2: 2nd rotamer of **4**

Route	:	# opt freq b3lyp/6-311g(d,p) scrf=(solvent=diethylether) geom=connectivity empiricaldispersion=gd3bj int=ultrafine pop=(regular,mk)	
SMILES	:	CC(C)(C)OC(=O)N1CCCCC1C#N	
Formula	:	$C_{11}H_{18}N_2O_2$	
Charge	:	0	
Multiplicity	:	1	
Dipole	:	10.6334	Debye
Energy	:	-690.23722685	a.u.
Gibbs Energy	:	-689.99767700	a.u.

4.2.1 Cartesian Co-ordinates (XYZ format)

33

C	-2.19942999	-1.52823198	-0.92590398
C	-1.94777906	0.95107299	-0.87313598
C	-2.27751899	0.95040399	0.63196301

C	-1.90434504	-0.37582800	1.29566097
C	-2.56467009	-1.54303801	0.55610001
H	-2.75032592	-2.27977204	-1.48428905
H	-1.12793803	-1.73520994	-1.05171597
H	-1.76865602	1.79247904	1.10586905
H	-3.35433292	1.12121999	0.71689802
H	-0.81543303	-0.50029999	1.28295505
H	-2.20956492	-0.36143401	2.34446597
H	-2.25435996	-2.49817705	0.98728597
H	-3.65309596	-1.47519004	0.65666699
N	-2.51416898	-0.22607900	-1.52752399
C	-2.95725894	-0.19089900	-2.83110809
O	-3.30607200	-1.17983103	-3.44632292
O	-2.96652389	1.06978703	-3.30195689
C	-3.41425490	1.37583399	-4.67815495
C	-2.50219107	0.67633998	-5.68374300
C	-4.88149691	0.98711300	-4.84534121
C	-3.23972011	2.88989401	-4.74771881
H	-1.45884395	0.93410802	-5.48766184
H	-2.61727595	-0.40431601	-5.63214016
H	-2.75472403	1.01163197	-6.69242477
H	-5.48753119	1.46014404	-4.06891680
H	-5.23798418	1.33643103	-5.81729794
H	-5.01006413	-0.09183200	-4.78951216
H	-3.53340697	3.25150394	-5.73511219
H	-3.86184692	3.38251090	-3.99752903
H	-2.19723892	3.16276693	-4.57223415
C	-0.48745099	1.00675297	-1.09986699
N	0.65114200	1.02595496	-1.27871597
H	-2.37563109	1.83588302	-1.33799005

4.2.2 Frequencies

Mode	IR frequency	IR intensity
1	17.44460000	2.32220000
2	34.36320000	1.30730000
3	72.77520000	4.71620000
4	103.26300000	3.39250000
5	112.96120000	2.22780000
6	125.71570000	1.82510000
7	143.22420000	2.24360000
8	208.34870000	0.11310000
9	224.03990000	6.89590000
10	247.79120000	1.69400000
11	256.41560000	0.74470000
12	273.13770000	0.07830000
13	278.70440000	5.00200000
14	302.57820000	0.32860000
15	342.93710000	20.94630000
16	351.33130000	3.56860000
17	367.96540000	1.74380000
18	398.31090000	6.17490000
19	419.86940000	0.39060000

20	443.46440000	3.06140000
21	458.70670000	3.87630000
22	500.46900000	0.43720000
23	511.74840000	9.10240000
24	570.46720000	7.18430000
25	625.67530000	5.29280000
26	721.33880000	9.18400000
27	764.50460000	8.01600000
28	776.79430000	32.34980000
29	808.40350000	5.46630000
30	825.51710000	4.70110000
31	857.02150000	5.17710000
32	868.13790000	41.87120000
33	877.07320000	47.19120000
34	929.55980000	0.16540000
35	930.23530000	1.60480000
36	937.50630000	17.56700000
37	946.41750000	3.52700000
38	972.01920000	0.15390000
39	1005.94360000	23.72770000
40	1049.61190000	68.88470000
41	1052.85700000	0.79100000
42	1058.02260000	0.91930000
43	1085.17330000	3.19730000
44	1110.88310000	77.35410000
45	1140.13320000	102.15560000
46	1154.43600000	19.38450000
47	1179.55690000	526.64760000
48	1204.64170000	17.60200000
49	1258.88350000	109.57610000
50	1270.67430000	21.65260000
51	1282.68800000	58.67620000
52	1292.42460000	8.62000000
53	1296.59390000	173.38470000
54	1309.49900000	7.82120000
55	1354.35270000	126.01730000
56	1365.12890000	22.70470000
57	1384.15510000	24.65430000
58	1388.45640000	0.97050000
59	1396.53880000	28.78480000
60	1398.48310000	32.88480000
61	1404.97780000	38.42350000
62	1423.07730000	45.00700000
63	1425.26150000	277.63870000
64	1467.66360000	0.42900000
65	1475.65040000	5.79040000
66	1483.87950000	12.27510000
67	1486.60530000	2.66810000
68	1487.30100000	0.03140000
69	1490.53320000	26.13810000
70	1493.06910000	6.45330000
71	1501.33780000	1.90000000
72	1507.22470000	9.87500000
73	1521.90510000	25.30290000
74	1744.01060000	566.24870000

75	2345.33470000	9.42640000
76	2992.57200000	42.44280000
77	3021.95330000	17.34910000
78	3030.47400000	47.93360000
79	3036.96030000	13.60840000
80	3039.14740000	33.26650000
81	3045.23980000	21.26030000
82	3045.97840000	21.31550000
83	3076.09320000	52.09850000
84	3079.61940000	57.68560000
85	3093.12770000	35.64910000
86	3100.56710000	9.69180000
87	3103.37830000	21.18420000
88	3109.91510000	45.09180000
89	3115.75810000	61.67400000
90	3132.23090000	5.38800000
91	3143.62190000	1.17360000
92	3146.89210000	20.05720000
93	3147.36680000	7.42030000

4.3 1st rotamer of 4 with chelated bridged Mg dimer

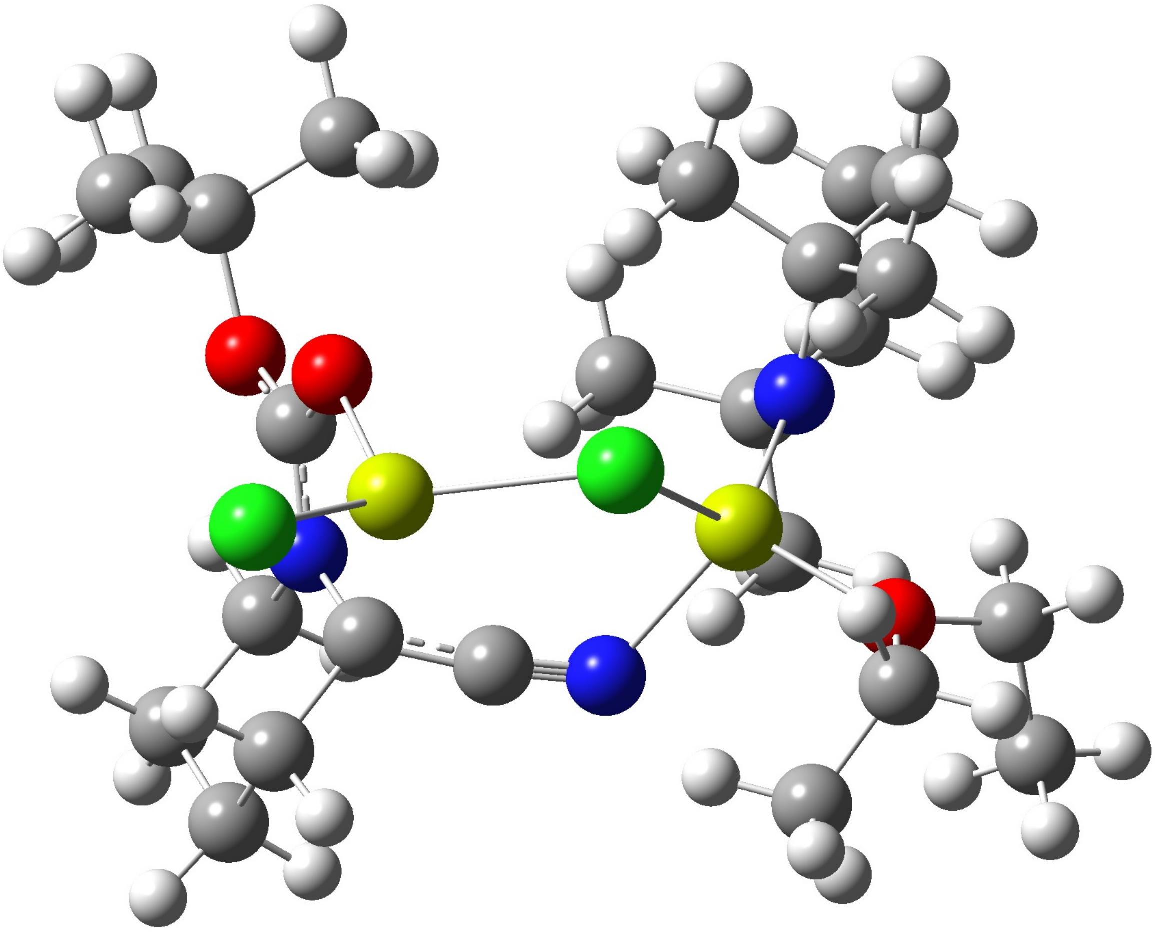

Figure S3: 1st rotamer of **4** with chelated bridged Mg dimer

Route	: # opt freq b3lyp/6-311g(d,p) scrf=(solvent=diethylether) geom=connectivity empiricaldispersion=gd3bj int=ultrafine pop=(regular,mk)	
SMILES	: CCO(CC)[Mg]1(N=[C][C]2CCCCN2[C](O[Mg](Cl1)Cl)OC(C)(C)C)N3C(CCCC3(C)C)(C)C	
Formula	: $C_{24}H_{45}Cl_2Mg_2N_3O_3$	
Charge	: 0	
Multiplicity	: 1	
Dipole	: 20.7324	Debye
Energy	: -2653.12437408	a.u.
Gibbs Energy	: -2652.52840400	a.u.

4.3.1 Cartesian Co-ordinates (XYZ format)

79

C	-2.91936994	-0.15763500	-2.52398491
C	-1.85763097	-1.68479395	-0.84978002
C	-2.68504000	-2.86707211	-1.35483694
C	-3.10280299	-2.67034006	-2.81997991
C	-3.79315591	-1.31501806	-3.01325893
H	-3.44895005	0.78915799	-2.57238007
H	-2.01458812	-0.07545400	-3.13666010
H	-2.11260796	-3.78945804	-1.23202503
H	-3.57533908	-2.96360302	-0.72563499
H	-2.21155596	-2.71903992	-3.45730805
H	-3.76836610	-3.47881103	-3.13536906
H	-4.03414106	-1.15141106	-4.06715822
H	-4.73742104	-1.30057096	-2.45770502
N	-2.50128889	-0.39829099	-1.13386905
C	-2.71107507	0.46449500	-0.11964700
O	-2.37700796	0.20244300	1.05927801
O	-3.28746200	1.59708905	-0.47566399
C	-3.70809197	2.63415909	0.51463097
C	-4.72567511	2.03221512	1.47828496
C	-2.47997999	3.19844389	1.21918595
C	-4.35339403	3.68080592	-0.38635701
H	-5.54615593	1.57044899	0.92443800
H	-4.26864290	1.28763199	2.12683296
H	-5.14001417	2.82963395	2.09927011
H	-1.75936306	3.56400490	0.48612899
H	-2.78677893	4.03840399	1.84670103
H	-1.99904299	2.45045304	1.84484994
H	-4.70525789	4.51799011	0.21918400
H	-3.63131595	4.05612087	-1.11385298
H	-5.20442915	3.25593591	-0.92200100
C	-0.50496602	-1.64486897	-1.20840394
N	0.65331900	-1.55160201	-1.35499597
Mg	-1.67487204	-1.65595102	1.44893003
Mg	1.91166103	-0.61963600	0.06940300
Cl	0.66671097	-1.53315496	2.00234103

N	2.36980891	1.29997396	-0.07423600
O	3.58796692	-1.76878905	0.19926800
C	2.02347589	2.05281806	-1.29019701
C	2.56161404	2.04298902	1.18441999
C	3.68112397	-3.09896398	0.77543402
H	3.30436993	-3.01791406	1.79589498
H	4.73774195	-3.36863804	0.81974399
C	4.79921007	-1.23170900	-0.39543799
H	4.59093904	-0.16603200	-0.50296801
H	5.61122417	-1.35839605	0.32544699
C	5.11799479	-1.87602603	-1.73056495
H	5.33083677	-2.94234991	-1.62877202
H	4.28593922	-1.75035906	-2.42660403
H	6.00127411	-1.39672196	-2.16089296
C	2.87848306	-4.10851622	-0.02274200
H	3.24319911	-4.18422222	-1.04838896
H	2.96013188	-5.09073496	0.45025799
H	1.82301402	-3.83262396	-0.04918600
C	1.23439300	2.42315507	1.89363098
H	0.60458398	1.53820801	2.00912189
H	1.42328095	2.83929110	2.88961697
H	0.66645700	3.16303802	1.33149803
C	3.40814400	3.32000208	0.98139799
H	3.43713307	3.89081907	1.91617501
H	4.43800306	3.01596308	0.75713903
C	2.86842895	3.33924794	-1.44027805
H	2.49570298	3.92287397	-2.28947806
H	3.89672494	3.04302192	-1.68092501
C	0.51718801	2.41828990	-1.38486004
H	-0.09373800	1.52534103	-1.22907400
H	0.22677200	3.15042710	-0.63255799
H	0.26739499	2.83507299	-2.36711597
C	2.34721088	1.16456604	-2.50520897
H	3.39661407	0.85998201	-2.47728610
H	1.72550297	0.26636699	-2.52780390
H	2.17044806	1.70420599	-3.44073892
C	3.34356689	1.13519204	2.15162492
H	3.54905009	1.65458405	3.09217906
H	2.78004909	0.23323800	2.41013503
H	4.29515505	0.83639300	1.70695400
C	2.89747405	4.18104076	-0.16910499
H	1.89882600	4.57008791	0.05744400
H	3.54288912	5.05492496	-0.30686599
Cl	-3.05321789	-3.10576296	2.58792901

4.3.2 Frequencies

Mode	IR frequency	IR intensity
1	16.89090000	1.21590000
2	22.27430000	0.77850000
3	27.12500000	0.88950000
4	32.93240000	0.82620000
5	38.81430000	0.66350000

6	40.63350000	0.89550000
7	44.46440000	0.90580000
8	49.26080000	1.60250000
9	56.87480000	8.62970000
10	61.88990000	3.17160000
11	66.17880000	3.00810000
12	70.38280000	4.19300000
13	72.95720000	7.18590000
14	83.30870000	2.97080000
15	92.81140000	3.82840000
16	94.32800000	1.75400000
17	97.68270000	3.25770000
18	116.66800000	7.73940000
19	119.74820000	1.17280000
20	122.94850000	1.58750000
21	128.68100000	1.50900000
22	134.84590000	3.72900000
23	136.81810000	2.75230000
24	158.41640000	0.78010000
25	167.99510000	13.97600000
26	172.91820000	1.18920000
27	191.45370000	18.01740000
28	200.68370000	1.43110000
29	201.21840000	3.67150000
30	205.62190000	15.64140000
31	211.95470000	1.01360000
32	218.31230000	2.76870000
33	239.49870000	1.39820000
34	244.33900000	0.56720000
35	253.40550000	4.71210000
36	258.53600000	8.05560000
37	266.10860000	1.93660000
38	266.62200000	20.62470000
39	272.54870000	0.72030000
40	275.95710000	6.84940000
41	285.08200000	1.12950000
42	293.53600000	21.70010000
43	294.38420000	25.89960000
44	301.36870000	12.50690000
45	305.27950000	12.68570000
46	319.36060000	47.75460000
47	321.56660000	19.23680000
48	326.65970000	9.20220000
49	337.58750000	10.94080000
50	343.27040000	13.38600000
51	351.05300000	8.31420000
52	364.24440000	7.49370000
53	376.97010000	144.01020000
54	392.04540000	25.84330000
55	402.20560000	40.94910000
56	412.97540000	93.97470000
57	413.40600000	0.81320000
58	419.69010000	1.93620000
59	425.01630000	31.28870000
60	431.79260000	42.84470000

61	441.55100000	35.51890000
62	456.53190000	3.66740000
63	469.57650000	2.73210000
64	475.08850000	80.54420000
65	491.39120000	29.17450000
66	496.70860000	0.68510000
67	510.33500000	33.37910000
68	517.05270000	36.34640000
69	527.56980000	25.25130000
70	540.19090000	57.59950000
71	549.48440000	16.78150000
72	590.71690000	5.75670000
73	620.83370000	10.81660000
74	653.56920000	11.60850000
75	696.52620000	36.55990000
76	739.57700000	43.34980000
77	744.50300000	18.87400000
78	759.99720000	0.91170000
79	773.99100000	40.10670000
80	786.59860000	47.87490000
81	803.53970000	12.41710000
82	811.16910000	14.11750000
83	823.88610000	9.37640000
84	844.00150000	59.31780000
85	846.87360000	11.28660000
86	859.89670000	11.56220000
87	860.18390000	5.28410000
88	866.39870000	4.25440000
89	908.83370000	4.12360000
90	909.80830000	42.76750000
91	910.93430000	2.28670000
92	911.29740000	17.53450000
93	915.72170000	3.59440000
94	920.65560000	42.47100000
95	924.58860000	16.92310000
96	931.20350000	0.16450000
97	935.30940000	8.40690000
98	953.16570000	13.87680000
99	961.74910000	4.27620000
100	975.36080000	0.39320000
101	976.37800000	7.35760000
102	1005.37030000	1.72680000
103	1008.26160000	0.32380000
104	1014.38080000	50.93400000
105	1036.15540000	58.07710000
106	1054.11540000	4.00370000
107	1054.48070000	21.98980000
108	1055.28080000	206.63280000
109	1057.83430000	46.09230000
110	1062.48310000	1.33310000
111	1068.29840000	2.02370000
112	1069.72760000	3.25810000
113	1090.17850000	20.49500000
114	1093.99740000	4.11280000
115	1108.87170000	27.78320000

116	1114.36350000	13.74610000
117	1148.34710000	83.84020000
118	1151.72000000	7.71550000
119	1159.63880000	0.86680000
120	1177.95290000	37.92040000
121	1179.20000000	435.26650000
122	1190.96180000	15.89490000
123	1193.47050000	34.88110000
124	1213.76120000	0.17840000
125	1218.18000000	7.86220000
126	1220.49360000	14.61710000
127	1230.30470000	56.25190000
128	1244.39330000	3.53580000
129	1258.19120000	19.50260000
130	1258.52870000	55.08760000
131	1260.48770000	44.73210000
132	1274.89600000	26.07540000
133	1282.18410000	64.61110000
134	1306.70850000	121.88390000
135	1314.94690000	5.11340000
136	1318.87550000	8.75860000
137	1319.34510000	7.47480000
138	1347.99010000	6.15410000
139	1360.33630000	5.24050000
140	1364.18090000	0.38840000
141	1369.25320000	1.61950000
142	1372.02710000	5.55430000
143	1376.30790000	0.79580000
144	1382.19010000	2.04470000
145	1382.43270000	0.92360000
146	1389.66320000	30.16710000
147	1390.71590000	23.32970000
148	1400.32650000	9.21620000
149	1400.96340000	23.74520000
150	1402.34690000	20.54580000
151	1405.10590000	21.55610000
152	1408.79640000	3.61930000
153	1409.47750000	3.61000000
154	1422.97520000	19.84080000
155	1425.74630000	19.59750000
156	1426.20330000	15.83260000
157	1467.82200000	1.67680000
158	1470.15800000	1.30830000
159	1472.42620000	205.56860000
160	1475.77850000	43.53480000
161	1476.69100000	250.22370000
162	1478.28140000	8.22700000
163	1480.84670000	9.07180000
164	1482.34730000	2.23150000
165	1482.60460000	4.14230000
166	1483.32370000	4.74080000
167	1484.26620000	3.23160000
168	1486.31430000	0.86470000
169	1487.89290000	1.32550000
170	1489.79140000	43.50040000

171	1490.55540000	26.59360000
172	1492.34320000	2.13140000
173	1493.48680000	7.61700000
174	1495.01920000	4.71970000
175	1499.54050000	5.11620000
176	1500.57300000	4.23710000
177	1502.73710000	7.68600000
178	1507.06200000	71.41680000
179	1507.90510000	58.21390000
180	1510.00140000	10.12570000
181	1511.09760000	9.09350000
182	1516.18390000	47.24860000
183	1519.39830000	16.52660000
184	1524.35430000	101.39910000
185	1623.63130000	561.10980000
186	2203.87380000	712.57250000
187	2995.99510000	34.62840000
188	2998.82080000	60.44240000
189	3010.64550000	29.66310000
190	3012.14300000	34.60210000
191	3013.04530000	40.33890000
192	3014.35320000	52.24530000
193	3018.98070000	17.42250000
194	3022.00530000	94.05440000
195	3023.28020000	16.04440000
196	3027.61930000	48.29910000
197	3033.05610000	21.07890000
198	3034.46130000	15.14080000
199	3036.57750000	80.16890000
200	3037.30910000	88.59780000
201	3040.96040000	27.54610000
202	3041.30360000	13.24600000
203	3041.63540000	23.27790000
204	3045.08000000	25.69200000
205	3050.35630000	115.91330000
206	3053.00080000	17.50060000
207	3060.51090000	34.33420000
208	3063.58550000	83.03670000
209	3072.73610000	57.18950000
210	3075.07450000	16.05680000
211	3076.65330000	23.92720000
212	3081.83200000	36.16780000
213	3082.40500000	129.08000000
214	3083.93400000	103.56720000
215	3090.14560000	9.01590000
216	3098.16370000	47.69900000
217	3099.79990000	45.69480000
218	3101.96980000	2.28910000
219	3102.16120000	39.62700000
220	3106.42930000	11.09600000
221	3108.19070000	34.84970000
222	3112.74120000	9.07080000
223	3113.96390000	22.35130000
224	3115.06210000	35.97590000
225	3115.79940000	40.02820000

226	3120.60210000	59.41640000
227	3126.81430000	45.27930000
228	3133.72210000	51.43740000
229	3145.76820000	9.43790000
230	3153.89420000	8.80420000
231	3156.59220000	11.89650000

4.4 2nd rotamer of 4 with non-chelated bridged Mg dimer

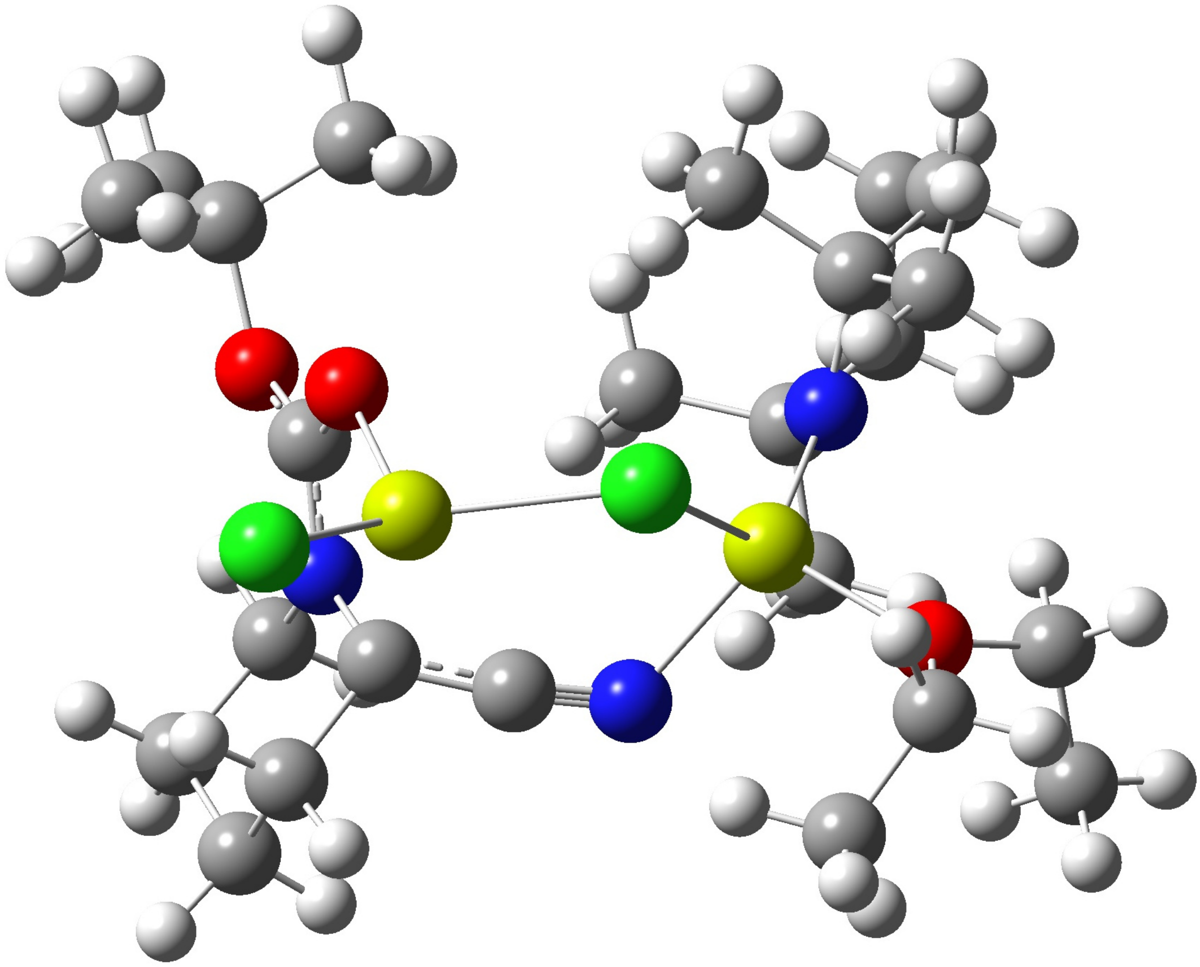

Figure S4: 2nd rotamer of **4** with non-chelated bridged Mg dimer

Route	:	# opt freq b3lyp/6-311g(d,p) scrf=(solvent=diethylether) geom=connectivity empiricaldispersion=gd3bj int=ultrafine pop=(regular,mk)	
SMILES	:	CCO(CC)[Mg](Cl)Cl[Mg]([N][C][C]1CCCCN1C(=O)OC(C)(C)C)(N2C(CCCC2(C)C)(C)C)O(CC)CC	
Formula	:	$C_{28}H_{55}Cl_2Mg_2N_3O_4$	
Charge	:	0	
Multiplicity	:	1	
Dipole	:	37.8604	Debye
Energy	:	-2886.87250936	a.u.
Gibbs Energy	:	-2886.14305300	a.u.

4.4.1 Cartesian Co-ordinates (XYZ format)

94

C	-3.83969092	1.40514696	1.12875795
C	-2.11899090	0.66254503	-0.43640700
C	-3.12154603	0.54623801	-1.59571004
C	-4.52029085	0.25569701	-1.03518701
C	-4.91840506	1.26903498	0.04745500
H	-3.74675202	0.49037600	1.71946895
H	-4.07146215	2.21526003	1.81486297
H	-3.13703704	1.48952401	-2.15812397
H	-2.81519103	-0.24036500	-2.28857589
H	-5.25604391	0.26301599	-1.84462595
H	-4.52376699	-0.75131202	-0.60786301
H	-5.07313681	2.25267601	-0.40953901
H	-5.86609411	0.97910100	0.51139098
N	-2.53813195	1.69379902	0.51409501
C	-2.20226598	3.02567410	0.39010900
O	-2.70745707	3.91497993	1.05317402
O	-1.23810196	3.20019007	-0.53240800
C	-0.59475702	4.51052809	-0.74166900
C	-1.62614799	5.52896976	-1.22475100
C	0.10632100	4.94768095	0.54260498
C	0.42058200	4.20685005	-1.83972299
H	-2.14951301	5.14463377	-2.10386109
H	-2.35364103	5.74915314	-0.44675699
H	-1.11522198	6.45252609	-1.50825298
H	0.79886800	4.17187595	0.87480801
H	0.67659599	5.86007881	0.35168299
H	-0.61528498	5.14291000	1.33378303
H	0.98250598	5.11077118	-2.08457398
H	1.11688197	3.43539906	-1.51150799
H	-0.08625400	3.85539889	-2.74100304
C	-0.77456301	0.68422300	-0.81249797
N	0.34313801	0.52633399	-1.11887097
Mg	-1.83989799	-1.30967605	0.74046302
Mg	1.80675197	-0.84441203	-0.51397699
Cl	-1.66291499	-1.04820001	3.03084803
Cl	0.07208100	-2.56310105	-0.08270200
N	3.30015802	-0.31987900	0.67442900
O	-3.28869700	-2.75503397	0.40332901
O	2.54906392	-1.69477201	-2.21970296
C	3.55533195	1.11039901	0.91570097
C	3.72177792	-1.29389596	1.69702601
C	1.82150602	-2.54150510	-3.14659190
H	1.41671097	-3.36727810	-2.56009889
H	2.53761196	-2.94746494	-3.86293507
C	3.93877292	-1.42610097	-2.54635191
H	4.34933090	-1.01260602	-1.62383902
H	4.42700195	-2.38266206	-2.75190210
C	4.07555819	-0.45641801	-3.70398593
H	3.66629291	-0.86443901	-4.63093710
H	3.56500196	0.48226500	-3.47935510
H	5.13406086	-0.24027900	-3.87122893
C	0.71384001	-1.77238095	-3.84075308
H	1.11417401	-0.94207102	-4.42499304

H	0.17080399	-2.44253111	-4.51266718
H	0.00693700	-1.37192595	-3.11277509
C	-4.13779116	-3.14217997	1.52667105
H	-3.77376604	-2.57283092	2.38001895
H	-3.96213102	-4.20139217	1.72835195
C	-5.59726095	-2.84546208	1.24368000
H	-5.95618677	-3.37076211	0.35572001
H	-6.20253086	-3.16965294	2.09397888
H	-5.75374222	-1.77477705	1.10221195
C	-3.19265795	-3.78062797	-0.62595499
H	-2.37475991	-4.45086098	-0.35066599
H	-4.12655783	-4.34416723	-0.61680198
C	-2.96873498	-3.15831304	-1.98750305
H	-2.02891302	-2.60596991	-2.02589297
H	-2.91318989	-3.95438290	-2.73398495
H	-3.79175401	-2.49350500	-2.25353289
C	2.74865103	-1.40451396	2.90111399
H	1.72927201	-1.58283305	2.55046797
H	3.03023100	-2.23394394	3.55979300
H	2.73329711	-0.49755901	3.50456190
C	5.14488411	-1.00015295	2.22242093
H	5.37950802	-1.68377697	3.04589510
H	5.85836601	-1.20881701	1.41540504
C	4.97430706	1.36106205	1.47664404
H	5.07550097	2.41652894	1.75288701
H	5.69626093	1.16859603	0.67322099
C	2.50811195	1.78179801	1.84350896
H	1.50113499	1.61002505	1.45409703
H	2.54083800	1.38666499	2.85798097
H	2.66848707	2.86427593	1.90383101
C	3.47723699	1.83664799	-0.44000700
H	4.20245600	1.41221595	-1.13854396
H	2.47991705	1.75462699	-0.88012701
H	3.69179010	2.90355897	-0.32542801
C	3.76014495	-2.67951393	1.02679098
H	4.10226822	-3.44497705	1.72953498
H	2.76676512	-2.99066710	0.68580598
H	4.43621492	-2.66941595	0.16895901
C	5.31500816	0.45319799	2.65376401
H	4.66939116	0.67644399	3.50975800
H	6.34217310	0.63405102	2.98797297

4.4.2 Frequencies

**Mode**	**IR frequency**	**IR intensity**
1	16.32510000	0.30200000
2	23.71730000	1.05840000
3	28.40010000	0.51530000
4	32.32260000	2.11380000
5	35.45070000	1.11630000
6	43.68810000	0.13790000
7	46.71200000	1.66780000
8	51.49540000	0.23330000

9	55.51220000	0.75380000
10	58.01250000	1.60350000
11	60.91650000	0.91030000
12	67.11270000	0.57740000
13	71.29220000	3.44420000
14	74.27830000	1.85150000
15	81.60060000	3.28690000
16	87.63800000	2.78760000
17	93.30620000	6.44910000
18	94.80410000	4.00740000
19	101.36420000	1.50370000
20	108.41380000	2.18060000
21	116.87890000	4.04030000
22	118.42250000	5.66070000
23	123.42580000	1.13230000
24	126.36150000	1.87700000
25	129.87700000	2.98970000
26	132.97820000	13.65510000
27	139.67320000	4.49860000
28	144.28290000	4.70660000
29	151.37400000	7.62820000
30	163.02720000	5.98740000
31	168.46690000	2.36300000
32	172.63550000	1.38770000
33	181.25040000	14.33030000
34	194.29520000	7.35760000
35	196.66490000	2.27160000
36	205.84080000	4.41850000
37	207.62770000	0.18470000
38	214.68040000	2.47720000
39	224.25750000	11.59720000
40	232.63690000	5.11310000
41	236.50070000	4.42350000
42	242.15950000	0.51050000
43	248.91570000	0.51410000
44	264.40750000	20.40820000
45	267.83350000	8.08260000
46	271.66050000	0.02040000
47	275.11920000	8.49290000
48	282.46490000	5.95190000
49	291.26250000	57.66560000
50	293.08360000	23.48700000
51	304.65430000	16.03350000
52	307.87980000	6.09540000
53	310.70420000	5.76340000
54	313.89550000	51.59290000
55	325.67560000	18.49690000
56	327.16110000	47.01610000
57	328.42810000	22.18310000
58	330.04360000	36.74880000
59	344.45120000	10.28240000
60	348.29390000	23.32690000
61	350.22590000	2.46780000
62	353.88550000	20.38800000
63	364.76100000	23.20860000

64	378.50850000	12.54670000
65	396.18330000	28.23140000
66	403.02660000	110.00490000
67	411.56650000	10.43410000
68	416.05910000	1.14050000
69	419.31700000	12.48170000
70	427.48810000	20.51690000
71	436.53600000	12.66940000
72	450.34800000	17.85860000
73	457.69530000	10.26240000
74	461.47450000	16.99310000
75	470.65220000	4.79420000
76	473.59280000	86.42520000
77	499.45290000	1.80140000
78	501.90910000	5.83810000
79	511.44580000	40.05460000
80	515.07570000	14.33350000
81	527.90790000	21.58470000
82	538.08390000	29.47000000
83	540.82160000	64.08560000
84	556.30620000	3.76550000
85	590.86620000	5.42590000
86	598.94490000	13.37110000
87	608.98490000	20.82760000
88	670.47030000	51.34670000
89	743.57360000	7.96180000
90	760.47040000	1.20460000
91	767.58070000	4.54680000
92	773.52360000	30.15980000
93	786.19070000	41.87510000
94	794.61970000	25.52390000
95	797.91110000	19.41100000
96	812.96650000	16.14650000
97	821.38140000	10.88670000
98	843.10550000	7.92940000
99	847.11720000	11.12330000
100	851.79340000	11.12560000
101	859.66960000	13.61030000
102	862.52950000	1.08530000
103	867.64130000	4.77190000
104	874.18670000	56.82460000
105	902.68700000	6.78780000
106	909.57640000	0.88520000
107	910.51790000	49.61290000
108	912.84940000	21.20160000
109	913.26630000	38.42620000
110	916.25730000	5.77700000
111	918.54950000	28.25620000
112	928.83630000	8.37250000
113	929.00040000	2.89970000
114	932.00430000	6.20590000
115	962.17060000	19.73820000
116	962.71230000	17.71930000
117	971.31260000	0.06290000
118	976.00250000	6.57930000

119	1009.33780000	1.69140000
120	1013.13430000	0.49630000
121	1015.33280000	46.50020000
122	1021.95630000	85.91880000
123	1036.50620000	65.91150000
124	1039.79870000	41.79190000
125	1046.66200000	180.45440000
126	1052.67660000	0.39410000
127	1055.55680000	206.35250000
128	1060.96700000	3.65490000
129	1068.18380000	2.00520000
130	1071.71880000	5.46780000
131	1075.24590000	28.68710000
132	1095.08080000	4.31930000
133	1106.95800000	20.53470000
134	1107.78730000	4.23380000
135	1108.52990000	33.05300000
136	1114.75890000	12.72780000
137	1131.80500000	3.79380000
138	1137.34640000	117.47180000
139	1147.19780000	90.88330000
140	1164.53590000	77.41490000
141	1174.46450000	20.35070000
142	1177.99230000	47.94660000
143	1185.22690000	33.99580000
144	1186.86780000	411.89740000
145	1194.36270000	19.33540000
146	1214.68300000	0.12850000
147	1214.87440000	21.42220000
148	1217.56080000	7.90180000
149	1220.62960000	15.02070000
150	1238.69280000	32.23160000
151	1245.75910000	2.67910000
152	1258.24980000	84.12100000
153	1260.76910000	50.11120000
154	1270.12010000	19.12510000
155	1272.27890000	46.58260000
156	1278.72950000	78.61780000
157	1296.00310000	189.68650000
158	1310.76590000	22.72020000
159	1314.88550000	4.15370000
160	1320.25140000	17.42540000
161	1323.97870000	2.25310000
162	1332.42500000	11.45870000
163	1346.95090000	5.71810000
164	1354.83790000	5.54730000
165	1364.54900000	0.26560000
166	1371.92580000	5.06510000
167	1373.87490000	17.93200000
168	1376.56280000	0.66070000
169	1384.48720000	153.27150000
170	1385.97760000	0.88700000
171	1391.05340000	20.83140000
172	1394.19620000	25.90200000
173	1395.96210000	43.91720000

174	1398.94030000	23.27170000
175	1400.19880000	1.42300000
176	1402.05490000	53.37200000
177	1406.54490000	5.25920000
178	1406.88500000	21.91070000
179	1408.46190000	1.65970000
180	1415.17380000	3.12260000
181	1415.55360000	9.29280000
182	1422.58810000	23.79540000
183	1422.80650000	24.13960000
184	1423.65070000	13.48020000
185	1427.49030000	16.94670000
186	1440.87730000	17.06330000
187	1467.33620000	0.70480000
188	1471.67150000	0.77580000
189	1477.59190000	2.67650000
190	1478.58020000	3.47650000
191	1478.70450000	5.51540000
192	1482.69740000	4.81110000
193	1483.33670000	3.59800000
194	1484.15550000	9.31630000
195	1484.84010000	1.66090000
196	1485.70390000	6.76790000
197	1486.04670000	1.54970000
198	1486.89670000	4.62170000
199	1488.85870000	3.59130000
200	1491.03270000	23.58470000
201	1493.49760000	1.94610000
202	1493.67860000	1.00560000
203	1494.77790000	7.57520000
204	1495.57210000	2.30680000
205	1499.26070000	4.19110000
206	1500.41700000	1.23970000
207	1501.42220000	5.71440000
208	1503.03370000	9.45110000
209	1507.52380000	7.61010000
210	1510.57310000	15.11480000
211	1510.99540000	2.75750000
212	1514.40600000	10.65560000
213	1516.13710000	11.11020000
214	1517.59620000	3.40450000
215	1520.48770000	12.25580000
216	1522.70060000	25.81500000
217	1523.71280000	9.62950000
218	1547.57300000	30.13200000
219	1566.88090000	6.95350000
220	1726.74000000	595.59660000
221	2220.41550000	781.40480000
222	2989.95290000	54.65410000
223	2995.44210000	28.51330000
224	2997.43160000	67.37670000
225	3011.47160000	33.42200000
226	3012.09290000	56.44370000
227	3015.10640000	55.68790000
228	3018.85160000	60.49950000

229	3021.35800000	38.44680000
230	3024.39410000	47.57880000
231	3036.01760000	31.51530000
232	3036.27820000	65.53110000
233	3036.50360000	64.70270000
234	3036.86680000	10.02940000
235	3037.10700000	73.66160000
236	3039.19210000	33.89220000
237	3039.38440000	39.30930000
238	3041.11400000	18.84660000
239	3041.23580000	18.93870000
240	3047.50620000	29.72630000
241	3048.10810000	14.22500000
242	3050.87900000	16.40220000
243	3051.95160000	107.74830000
244	3058.61070000	47.20240000
245	3059.29320000	32.40190000
246	3061.45770000	35.82810000
247	3070.69350000	52.05210000
248	3076.08670000	37.22270000
249	3076.16080000	19.25890000
250	3080.10970000	33.36580000
251	3085.13200000	103.18450000
252	3088.05850000	82.36810000
253	3088.54840000	116.05220000
254	3089.62850000	8.58910000
255	3094.77190000	62.98170000
256	3097.24100000	15.98600000
257	3099.44110000	16.95800000
258	3101.16840000	21.82820000
259	3102.26400000	3.10460000
260	3102.69780000	59.49990000
261	3103.39970000	30.81640000
262	3107.21500000	16.48500000
263	3109.37190000	25.15610000
264	3110.73340000	68.07940000
265	3111.67720000	13.18560000
266	3112.46630000	43.53370000
267	3114.37860000	27.34100000
268	3117.21760000	24.69190000
269	3128.87150000	51.42160000
270	3131.36050000	60.23690000
271	3133.48250000	18.49710000
272	3133.52560000	17.46700000
273	3135.20670000	25.86370000
274	3141.91570000	5.18810000
275	3146.17820000	8.22550000
276	3147.41710000	15.07490000

4.5 1st rotamer of 4 with chelated Mg non-bridged dimer

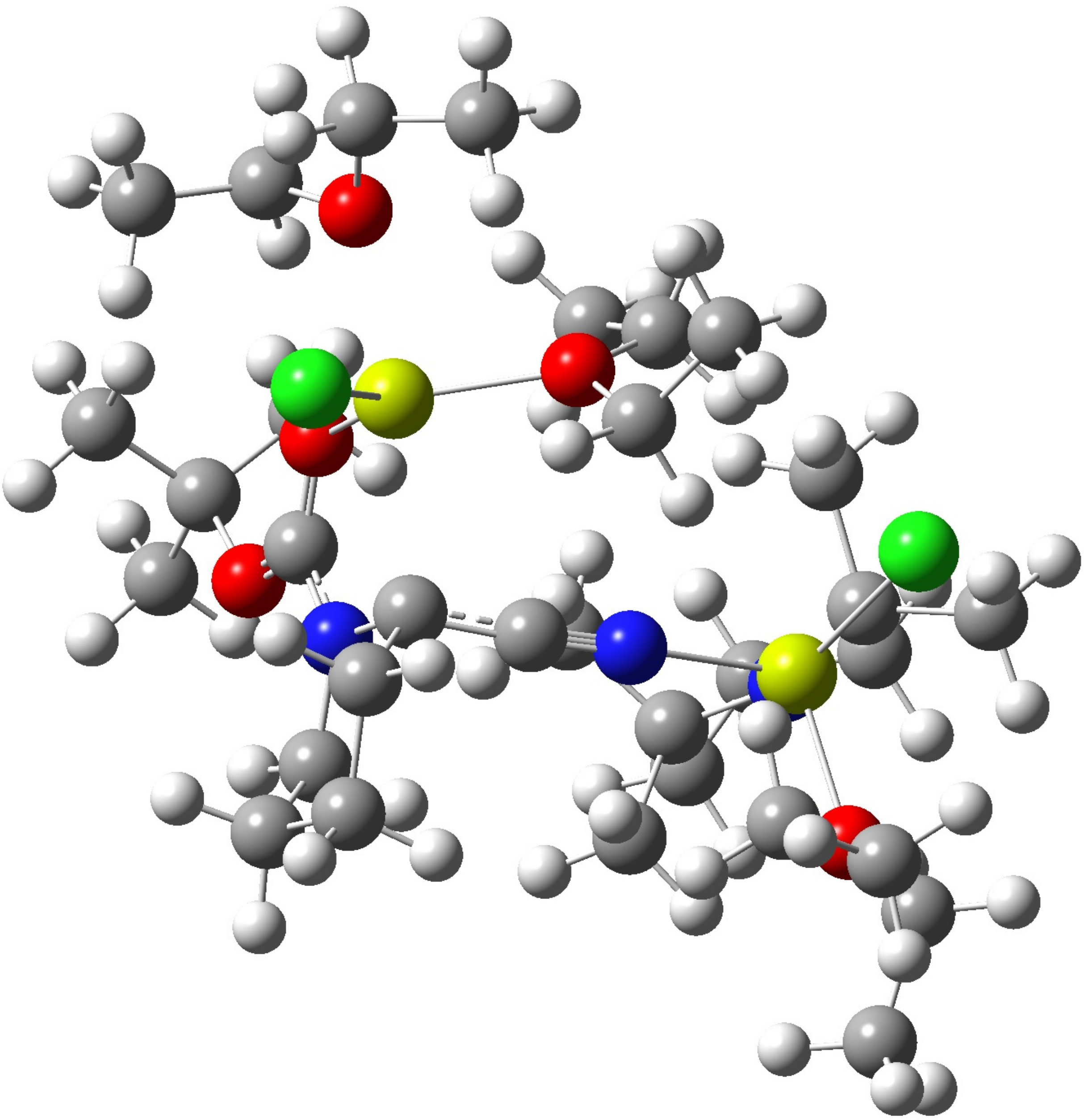

Figure S5: 1st rotamer of **4** with chelated non-bridged Mg dimer.

Route	: # opt freq b3lyp/6-311g(d,p) scrf=(solvent=diethylether) geom=connectivity empiricaldispersion=gd3bj int=ultrafine pop=(regular,mk)
SMILES	: CCO(CC)[Mg]([N]#C[C]1CCCCN1[C](OC(C)(C)C)O[Mg](O(CC)CC)(O(CC)CC)Cl)(N2C(CCCC2(C)C)(C)C)Cl
Formula	: $C_{32}H_{65}Cl_2Mg_2N_3O_5$
Charge	: 0

Multiplicity	: 1	
Dipole	: 20.9834	Debye
Energy	: -3120.67341355	a.u.
Gibbs Energy	: -3119.81296300	a.u.

4.5.1 Cartesian Co-ordinates (XYZ format)

109

C	0.64409101	1.64736402	3.03396297
C	1.26434803	-0.45389000	1.79915595
C	1.55721700	-1.16510797	3.12176991
C	0.74979401	-0.56085300	4.27939177
C	0.97166002	0.95381302	4.35806799
H	0.90323102	2.70169711	3.06589007
H	-0.42580399	1.56628704	2.81505108
H	1.34775400	-2.23129010	3.01080990
H	2.62726688	-1.08609402	3.32870889
H	-0.31703100	-0.76441801	4.12348080
H	1.03054202	-1.03015494	5.22702312
H	0.35458899	1.39722002	5.14465523
H	2.01795793	1.15715098	4.61262083
N	1.38743603	1.00576901	1.93747604
C	2.06157994	1.68178201	0.99585003
O	2.71560597	1.10383999	0.09526800
O	1.96739495	2.99877405	1.09956706
C	2.62373710	3.94573808	0.16041000
C	4.13596296	3.82645202	0.30784801
C	2.13625693	3.69719100	-1.26448298
C	2.12618899	5.29373312	0.67140102
H	4.42909718	3.98777604	1.34741700
H	4.48583221	2.84794497	-0.00808100
H	4.62113094	4.58736706	-0.30752799
H	1.04610395	3.72000599	-1.30026996
H	2.51839209	4.49041986	-1.91099799
H	2.47787690	2.73877597	-1.64726698
H	2.55405211	6.09595490	0.06720700
H	1.03814197	5.34967613	0.60534197
H	2.42360592	5.44338703	1.71091795
C	-0.00255100	-0.73088700	1.25738204
N	-1.02145505	-0.93404198	0.73247403
Mg	2.84048295	-0.95600897	0.07246500
Mg	-2.86516809	-0.93978000	-0.25265199
Cl	4.22001505	-2.43611693	1.27389002
Cl	-2.72081208	-3.00766993	-1.39674306
N	-3.40314293	0.88285100	-0.90035599
O	1.51036596	-2.10561991	-1.04718006
O	-4.19635916	-1.49982405	1.26944196
C	-2.88121510	2.06371403	-0.19025201
C	-3.80493307	1.03701198	-2.30957198
C	-4.22736692	-2.84585690	1.80681396
H	-4.53024912	-3.52341008	1.00380898
H	-4.97708988	-2.87688804	2.59909606

C	-5.50852203	-0.88751400	1.14269197
H	-5.35118914	-0.05825000	0.45096701
H	-6.18333483	-1.61236703	0.67635900
C	-6.04064894	-0.39491901	2.47613192
H	-6.22278881	-1.20839202	3.18132591
H	-5.33994389	0.30819699	2.93015409
H	-6.99007416	0.12244700	2.31402898
C	-2.86525512	-3.21510792	2.35472298
H	-2.55275798	-2.50876904	3.12665892
H	-2.91756010	-4.21348000	2.79631591
H	-2.11254406	-3.23168397	1.56720102
C	0.77157497	-1.67155099	-2.21718311
H	-0.28775600	-1.85550594	-2.02980208
H	1.08562398	-2.28376007	-3.06626105
C	1.03986096	-0.20663901	-2.48286796
H	2.10095310	-0.02823500	-2.66363406
H	0.48424199	0.10309100	-3.36998391
H	0.70759702	0.41511300	-1.65051401
C	0.99265802	-3.32848310	-0.44574401
H	1.62705803	-3.49558806	0.42492700
H	-0.02698700	-3.12561488	-0.11907700
C	1.01790595	-4.52801180	-1.37464499
H	2.02143598	-4.74098682	-1.74337399
H	0.66834998	-5.39851379	-0.81366599
H	0.34359801	-4.39611006	-2.22174311
C	-2.61211395	0.95993298	-3.30124688
H	-2.05677700	0.03268700	-3.13782310
H	-2.95587206	0.96916801	-4.34205723
H	-1.91703296	1.78993201	-3.17352009
C	-4.59066391	2.34407711	-2.56636310
H	-4.75803995	2.46155095	-3.64277697
H	-5.57737207	2.24513507	-2.09741592
C	-3.69665194	3.34389496	-0.48835999
H	-3.20478892	4.20495987	-0.02148400
H	-4.68066311	3.23981810	-0.01510400
C	-1.38198602	2.35934806	-0.47327700
H	-0.78755403	1.46206295	-0.30155900
H	-1.21616006	2.67406511	-1.50349700
H	-0.99987698	3.14963293	0.18229701
C	-3.01320291	1.80598795	1.32212400
H	-4.06224585	1.66482699	1.58641803
H	-2.45861411	0.91871202	1.63546705
H	-2.62519908	2.65301800	1.89674497
C	-4.75261879	-0.12367900	-2.66252899
H	-5.11507320	-0.03213900	-3.69133091
H	-4.25375223	-1.09278297	-2.58043909
H	-5.61546278	-0.12140700	-1.99158394
C	-3.90406489	3.57224798	-1.98084295
H	-2.94563699	3.75223398	-2.48023391
H	-4.51187706	4.46758604	-2.14993811
O	4.26357317	-0.75765598	-1.57265306
C	5.10545921	-1.86999404	-1.98311698
H	5.68053198	-1.53165197	-2.84945011
H	5.79234600	-2.10513496	-1.16882598
C	4.26974392	-3.07586288	-2.34154606

H	4.92857790	-3.85494995	-2.73384094
H	3.76400495	-3.47157407	-1.46449995
H	3.52960205	-2.83084702	-3.10521603
C	4.92766285	0.51587999	-1.76186800
H	4.14837980	1.26414704	-1.65946901
H	5.30564404	0.54301798	-2.78814793
C	6.04106903	0.74851602	-0.75527298
H	5.65309715	0.69846398	0.26410401
H	6.84070921	0.01298100	-0.85551798
H	6.47517681	1.73920405	-0.91234797

4.5.2 Frequencies

Mode	IR frequency	IR intensity
1	-9.52770000	0.82300000
2	17.78970000	0.48620000
3	22.60170000	1.33210000
4	30.57210000	0.69770000
5	31.09910000	1.34120000
6	38.82870000	2.04940000
7	40.97240000	1.01600000
8	44.88660000	0.43130000
9	57.31480000	1.26710000
10	58.00330000	0.10700000
11	59.17340000	2.63110000
12	61.49300000	2.31550000
13	65.72930000	0.34730000
14	68.17040000	2.15050000
15	77.60230000	6.40830000
16	78.77120000	5.01400000
17	82.46780000	1.96860000
18	87.14590000	4.74990000
19	90.28510000	2.63450000
20	92.89830000	6.73150000
21	97.45470000	0.23630000
22	104.50530000	5.88440000
23	106.42030000	5.75130000
24	111.38730000	7.23480000
25	113.96320000	4.36700000
26	118.35310000	2.76080000
27	123.81140000	0.83120000
28	126.54500000	3.46420000
29	127.75400000	7.71230000
30	130.53320000	0.77890000
31	135.89430000	2.06830000
32	141.17750000	0.79810000
33	142.51730000	1.99460000
34	145.11880000	3.81240000
35	151.92660000	7.96560000
36	156.39400000	1.82480000
37	162.97810000	1.22910000
38	167.80430000	3.98170000
39	169.66390000	2.00380000

40	180.78730000	9.16540000
41	184.94670000	6.73080000
42	187.79980000	9.14240000
43	189.84910000	1.67160000
44	196.38630000	0.41060000
45	210.12650000	5.19990000
46	211.43850000	5.59590000
47	215.44320000	4.24740000
48	228.47640000	5.54210000
49	233.09000000	1.55300000
50	238.72260000	0.56820000
51	242.10360000	1.30580000
52	252.09080000	0.18230000
53	253.19660000	1.97390000
54	261.53000000	0.16000000
55	265.49090000	0.28510000
56	271.65380000	7.06030000
57	273.59860000	2.28840000
58	275.25530000	12.58020000
59	283.86100000	103.34360000
60	289.35170000	2.15530000
61	296.69050000	27.54700000
62	297.07540000	1.41040000
63	300.80610000	34.70840000
64	302.78520000	23.02680000
65	312.20990000	4.47830000
66	313.49040000	4.15670000
67	321.24550000	15.87910000
68	326.74970000	8.19960000
69	335.75430000	16.18200000
70	340.00890000	34.34790000
71	342.23880000	86.03930000
72	351.73490000	1.48460000
73	352.84560000	35.99510000
74	354.87120000	8.31120000
75	361.64450000	44.91470000
76	375.02320000	41.25710000
77	386.45060000	50.91620000
78	394.14750000	55.33550000
79	399.92340000	53.98630000
80	408.19160000	25.23440000
81	416.28180000	12.66710000
82	419.20390000	25.17410000
83	426.38820000	37.40560000
84	432.10220000	25.17370000
85	441.48720000	11.58060000
86	456.38480000	27.27110000
87	457.16830000	33.34070000
88	469.85370000	4.22080000
89	477.36550000	47.42510000
90	499.37140000	0.76980000
91	503.73330000	22.40320000
92	508.13100000	18.21280000
93	518.31050000	11.63840000
94	523.55180000	16.82030000

95	526.03600000	23.16620000
96	534.30720000	52.26720000
97	547.40210000	23.20380000
98	591.96450000	2.98270000
99	629.28430000	6.33810000
100	656.83870000	11.18080000
101	701.20350000	47.41770000
102	738.59480000	6.07080000
103	749.46660000	31.64100000
104	759.08900000	0.74980000
105	774.98670000	37.26030000
106	799.44620000	47.72650000
107	804.42740000	31.59230000
108	805.52170000	12.05660000
109	806.52130000	23.83940000
110	815.28780000	9.97820000
111	821.22880000	6.14390000
112	823.09870000	11.81050000
113	834.25340000	3.17990000
114	841.02280000	10.60810000
115	844.44860000	16.00090000
116	846.40950000	17.05910000
117	846.79260000	47.58950000
118	859.04570000	1.95280000
119	860.78450000	16.26030000
120	867.19630000	5.16320000
121	903.26440000	59.22700000
122	908.57540000	1.08430000
123	913.31840000	9.76240000
124	913.41110000	26.59790000
125	915.22260000	9.02120000
126	915.54540000	26.07210000
127	916.55580000	36.37890000
128	917.40330000	2.21840000
129	924.52630000	20.12710000
130	930.27300000	0.13810000
131	933.89180000	13.95430000
132	954.99130000	14.01070000
133	963.19420000	6.50190000
134	972.35090000	0.25250000
135	979.03850000	6.91040000
136	1007.51410000	1.91850000
137	1019.88530000	0.14870000
138	1027.51490000	49.79920000
139	1028.78980000	77.54400000
140	1033.20300000	16.93420000
141	1034.17540000	71.65400000
142	1049.49390000	30.39710000
143	1052.17780000	5.72850000
144	1054.51260000	8.56510000
145	1057.57100000	143.59990000
146	1057.80770000	162.57000000
147	1062.04860000	213.27180000
148	1062.41790000	33.41460000
149	1068.63220000	1.68850000

150	1072.09200000	2.87970000
151	1089.88920000	29.43040000
152	1095.04060000	4.56020000
153	1106.35250000	18.80070000
154	1111.55060000	47.99570000
155	1113.51970000	24.22440000
156	1120.67940000	14.10080000
157	1127.37460000	11.92690000
158	1133.11930000	11.51500000
159	1146.81610000	97.27240000
160	1148.08410000	11.80680000
161	1155.92270000	0.79520000
162	1177.21870000	104.93130000
163	1179.84200000	148.30260000
164	1180.41090000	177.46210000
165	1180.72820000	20.78940000
166	1187.88360000	72.89390000
167	1192.32250000	33.30310000
168	1210.97500000	15.29260000
169	1212.01130000	18.06650000
170	1212.70710000	27.81950000
171	1215.09360000	1.19790000
172	1217.75920000	8.98960000
173	1226.21140000	61.73970000
174	1242.43750000	5.83240000
175	1252.60180000	3.23660000
176	1257.74670000	82.31970000
177	1261.19580000	48.77260000
178	1273.39700000	27.14520000
179	1280.12170000	45.65770000
180	1304.56630000	111.06580000
181	1308.85330000	13.73000000
182	1315.17310000	3.49130000
183	1317.60150000	1.22050000
184	1323.15780000	0.90070000
185	1325.39870000	3.89490000
186	1334.91000000	14.38750000
187	1341.18190000	9.54850000
188	1347.50730000	13.80170000
189	1355.02470000	2.61740000
190	1363.41450000	0.36980000
191	1366.47840000	0.68510000
192	1371.39160000	5.74930000
193	1376.00790000	0.76580000
194	1380.37440000	7.77090000
195	1382.90000000	1.01290000
196	1391.32070000	21.30020000
197	1394.34610000	21.08770000
198	1398.95250000	19.04840000
199	1401.52660000	1.56110000
200	1402.10960000	19.39740000
201	1402.52460000	13.97360000
202	1404.37600000	12.23430000
203	1405.37720000	6.25450000
204	1407.78300000	4.93020000

205	1409.99550000	0.38330000
206	1415.61680000	5.19560000
207	1417.41640000	4.87190000
208	1424.05890000	9.17410000
209	1425.89720000	36.91570000
210	1428.21380000	17.24700000
211	1433.58640000	7.40070000
212	1435.86920000	22.18040000
213	1437.56650000	23.11460000
214	1445.78620000	7.52480000
215	1467.22560000	35.34490000
216	1467.60950000	13.82460000
217	1471.08180000	1.31690000
218	1476.76120000	1.52110000
219	1478.85580000	45.50900000
220	1480.14440000	320.25940000
221	1480.73770000	49.53830000
222	1481.48120000	10.73260000
223	1481.70790000	19.62210000
224	1482.01260000	4.41630000
225	1484.64750000	2.02110000
226	1485.22510000	1.13880000
227	1485.43150000	1.70240000
228	1485.94530000	12.64360000
229	1488.00020000	14.64240000
230	1488.84510000	9.31040000
231	1489.88830000	35.23880000
232	1490.19280000	7.19390000
233	1491.54390000	3.16890000
234	1493.38700000	30.89870000
235	1495.26350000	13.20290000
236	1495.81580000	5.25840000
237	1496.50370000	2.40350000
238	1497.33130000	5.99280000
239	1499.38040000	12.12650000
240	1500.35640000	7.97740000
241	1502.21710000	14.61860000
242	1502.77670000	6.45020000
243	1503.89480000	1.15670000
244	1507.88060000	62.77770000
245	1508.92270000	11.84700000
246	1511.01580000	5.01290000
247	1511.56410000	4.05140000
248	1512.32440000	20.78580000
249	1514.90270000	22.81150000
250	1518.26350000	37.46620000
251	1518.35040000	52.91770000
252	1520.17730000	7.72460000
253	1524.31750000	85.92730000
254	1531.52860000	8.82200000
255	1632.72570000	684.47370000
256	2258.42610000	729.68190000
257	2996.17230000	32.16970000
258	2997.93310000	58.59830000
259	3005.95090000	34.47000000

260	3008.88040000	39.76660000
261	3012.45140000	40.13040000
262	3016.88810000	52.66980000
263	3019.43770000	60.95360000
264	3019.65610000	31.02640000
265	3026.32900000	72.13120000
266	3028.06660000	27.18920000
267	3034.27800000	104.79620000
268	3036.09670000	87.86150000
269	3036.68650000	18.67350000
270	3036.90540000	28.08320000
271	3037.11230000	16.91540000
272	3041.53930000	10.59630000
273	3041.72110000	26.90870000
274	3041.82160000	17.05130000
275	3042.77720000	18.18130000
276	3043.26410000	61.14000000
277	3043.93230000	47.25050000
278	3045.96990000	21.13880000
279	3046.88660000	12.59420000
280	3047.64440000	21.69320000
281	3048.93580000	117.20630000
282	3049.20240000	47.10700000
283	3052.77780000	15.34250000
284	3054.92890000	28.51660000
285	3059.37120000	91.29970000
286	3069.49970000	67.75570000
287	3073.54670000	32.16890000
288	3073.71590000	8.63550000
289	3080.87780000	113.41010000
290	3083.76020000	19.20500000
291	3085.76510000	46.24630000
292	3088.89770000	37.30070000
293	3090.58960000	23.61770000
294	3093.20390000	12.92520000
295	3094.45140000	26.08400000
296	3096.26360000	27.88330000
297	3096.51780000	70.73570000
298	3102.21310000	31.64700000
299	3106.22270000	11.87010000
300	3106.35910000	43.61440000
301	3106.54190000	17.06030000
302	3109.24590000	24.37530000
303	3110.58950000	58.23560000
304	3110.98700000	61.82300000
305	3113.71770000	28.65320000
306	3113.83100000	5.10180000
307	3115.88720000	15.50590000
308	3116.14490000	35.52780000
309	3117.56310000	40.74220000
310	3119.51520000	4.02760000
311	3119.66970000	16.51770000
312	3120.71130000	52.69810000
313	3129.97170000	51.56840000
314	3132.55590000	27.74960000

315	3136.02190000	25.60810000
316	3140.66760000	23.34650000
317	3149.04240000	12.00680000
318	3156.70460000	18.45860000
319	3157.32740000	17.52120000
320	3167.51830000	8.90960000
321	3172.49760000	10.33820000

4.6 2nd rotamer of 4 with non-chelated non-bridged Mg dimer

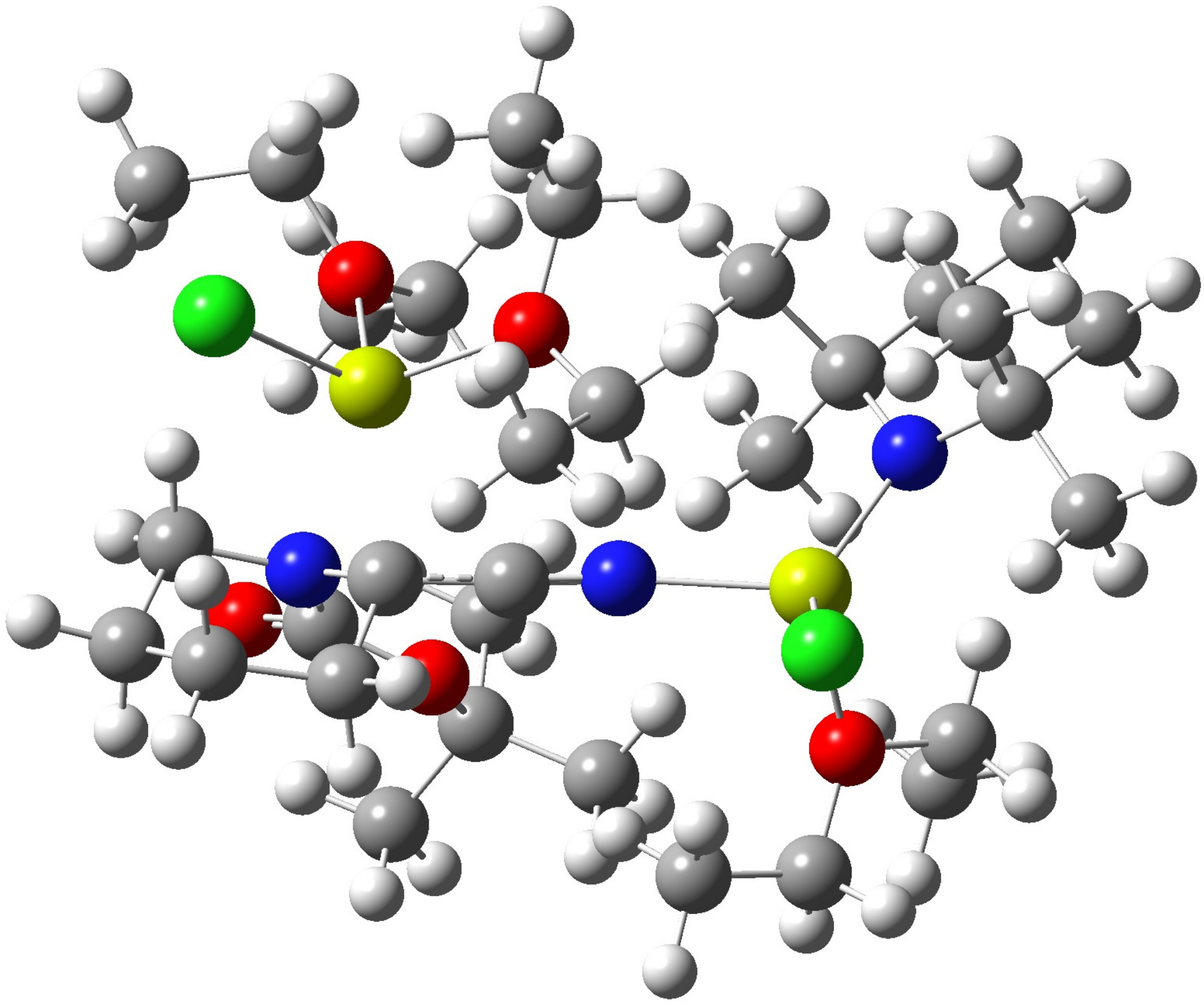

Figure 6: ***2nd rotamer of 4 with non-chelated non-bridged Mg dimer***

Route	: # opt freq b3lyp/6-311g(d,p) scrf=(solvent=diethylether) geom=connectivity empiricaldispersion=gd3bj int=ultrafine pop=(regular,mk)	
SMILES	: CCO(CC)[Mg](O(CC)CC)Cl.CCO(CC)[Mg]([N]#C[C]1CCCCN1C(=O)OC(C)(C)C)(N2C(CCCC2(C)C)(C)C)Cl	
Formula	: $C_{32}H_{65}Cl_2Mg_2N_3O_5$	
Charge	: 0	
Multiplicity	: 1	
Dipole	: 17.7328	Debye
Energy	: -3120.64945304	a.u.
Gibbs Energy	: -3119.78947500	a.u.

4.6.1 Cartesian Co-ordinates (XYZ format)

109

C	-4.30285501	1.34812403	-0.20562799
C	-2.09233093	0.71177602	-1.02565002
C	-2.46830201	1.05754697	-2.48167610
C	-3.98376393	0.92986000	-2.68394208
C	-4.76258612	1.71351099	-1.61976504
H	-4.54775095	0.30526099	0.01347200
H	-4.78910923	1.96455300	0.54472297
H	-2.15278006	2.08583689	-2.70375609
H	-1.93394995	0.39773199	-3.16976190
H	-4.25139189	1.28475404	-3.68293810
H	-4.27225924	-0.12523600	-2.63278008
H	-4.61354208	2.78948689	-1.76389694
H	-5.83462477	1.51872504	-1.71248603
N	-2.85083699	1.53492796	-0.07462300
C	-2.43517089	2.71025610	0.50381303
O	-3.15350509	3.38960910	1.22090304
O	-1.14727104	2.98513198	0.22637700
C	-0.44943899	4.08932209	0.92440802
C	-1.12994802	5.42547083	0.62988299
C	-0.38473600	3.77634501	2.41786909
C	0.94206899	4.05962086	0.30537301
H	-1.25247204	5.55087805	-0.44872400
H	-2.10427690	5.49121189	1.10652196
H	-0.49431399	6.23571205	0.99597102
H	0.09554700	2.80870605	2.57834601
H	0.21079400	4.54052782	2.92277002
H	-1.38033795	3.76278090	2.85733199
H	1.58616304	4.77455282	0.82090902
H	1.38459098	3.06829309	0.38320100
H	0.89767301	4.33690119	-0.74856502
C	-0.70288599	0.62614000	-0.83212101
N	0.43853000	0.40712801	-0.77843899
Mg	-2.67145705	-1.38626003	-0.39716101
Mg	2.48802590	-0.03167000	-0.98433501
Cl	-4.61259508	-2.49929404	-0.97242498
Cl	2.45936990	-0.95751399	-3.16565108
N	3.41215396	-0.84963399	0.59937900
O	-2.81775308	-1.22119498	1.64471602
O	3.26736212	1.89366698	-1.23549604
C	2.73999906	-0.78158098	1.90691900
C	4.36333513	-1.95337105	0.36666000
C	3.07893491	2.77116609	-2.37289190
H	3.87304592	2.55691504	-3.09565401
H	3.19506097	3.79990411	-2.02586198
C	4.53443289	2.10198689	-0.54954797
H	4.63612413	1.22028899	0.08508800
H	5.32919979	2.09527397	-1.30231500
C	4.56101513	3.37175608	0.28133100
H	4.45802402	4.27617693	-0.32095999
H	3.76924896	3.36015010	1.03125501
H	5.52142096	3.42792892	0.80080700
C	1.71168303	2.57380509	-2.99305391
H	0.91900402	2.74537301	-2.26636910

H	1.59527695	3.29160810	-3.80925894
H	1.60367894	1.56988800	-3.40178800
C	-1.85067999	-0.47554499	2.43503594
H	-1.17368901	-0.02911800	1.70923197
H	-1.28055704	-1.19885695	3.02240801
C	-2.47025609	0.58964199	3.31677389
H	-3.08541393	0.15804701	4.10784817
H	-1.66385198	1.15067804	3.79551005
H	-3.06937289	1.28975499	2.73533607
C	-3.75411010	-2.03409004	2.41409993
H	-3.83321595	-2.98278308	1.88304102
H	-3.30060196	-2.22137904	3.38963199
C	-5.11676407	-1.38023198	2.53018904
H	-5.55892706	-1.24911797	1.54237497
H	-5.77526712	-2.03209305	3.11082411
H	-5.06265306	-0.41244900	3.02829194
C	3.67569590	-3.28858900	-0.03075600
H	3.00400400	-3.11593103	-0.87525100
H	4.41333199	-4.04140091	-0.33124000
H	3.09026098	-3.71009898	0.78672802
C	5.28998184	-2.20105791	1.57993901
H	5.89328814	-3.09748912	1.39857602
H	5.98541212	-1.35583901	1.65387404
C	3.70567489	-1.04206395	3.08596611
H	3.13709998	-1.07717204	4.02238178
H	4.39215183	-0.18967600	3.15813589
C	1.52788603	-1.74417496	2.03197002
H	0.83866799	-1.58207500	1.20070004
H	1.83370697	-2.78940105	2.01204300
H	0.98501599	-1.57886195	2.96939301
C	2.19611692	0.64713699	2.09233904
H	3.01001906	1.37139797	2.02426195
H	1.44732106	0.89727199	1.33820796
H	1.71844006	0.75860298	3.07108903
C	5.27799606	-1.56022096	-0.80816698
H	6.02184105	-2.34075093	-0.99591100
H	4.71309519	-1.42464995	-1.73365998
H	5.80758810	-0.63189203	-0.58222097
C	4.53003883	-2.30975795	2.89649510
H	3.88228607	-3.19330502	2.89546800
H	5.22734880	-2.44101095	3.73079300
O	-1.13653195	-2.70865512	-0.61747199
C	-0.30742401	-2.80500197	-1.81817603
H	0.42880800	-3.58972096	-1.64231205
H	0.22507700	-1.86239803	-1.90034497
C	-1.12749696	-3.07628989	-3.06366897
H	-1.82652605	-2.26139593	-3.26947093
H	-0.44859099	-3.14823794	-3.91687608
H	-1.69435799	-4.00472021	-2.98422003
C	-0.99278402	-3.80439091	0.32443300
H	-1.44363201	-3.44342589	1.24903798
H	0.07412000	-3.94375300	0.50795501
C	-1.66367197	-5.07688522	-0.15507400
H	-1.18590903	-5.46710777	-1.05569398
H	-1.58399296	-5.84049177	0.62318999

H -2.71964693 -4.89915180 -0.36588401

4.6.2 Frequencies

Mode	IR frequency	IR intensity
1	7.71980000	0.19460000
2	23.03210000	0.83910000
3	26.46080000	0.25930000
4	36.29190000	0.45150000
5	41.41640000	0.92090000
6	42.83610000	0.58150000
7	46.34940000	0.06760000
8	48.42410000	0.67800000
9	52.14360000	0.50320000
10	56.08900000	2.63940000
11	58.72600000	1.96140000
12	65.09300000	0.71750000
13	70.32210000	7.53040000
14	71.22280000	8.08490000
15	73.96380000	2.73030000
16	76.46630000	4.03840000
17	82.04050000	1.49310000
18	83.36900000	5.30410000
19	89.61320000	1.23880000
20	92.96320000	2.78370000
21	101.95960000	7.27100000
22	102.43000000	1.01820000
23	105.28130000	3.76950000
24	107.22910000	10.95660000
25	111.71000000	4.77110000
26	114.80760000	6.65640000
27	118.68240000	3.28970000
28	121.13610000	2.16110000
29	130.49750000	0.25200000
30	132.10910000	4.13110000
31	134.19470000	4.54380000
32	139.08860000	1.64590000
33	142.14310000	2.00530000
34	143.30150000	10.13930000
35	151.16520000	1.41460000
36	163.11580000	2.40570000
37	163.86170000	9.52950000
38	170.76860000	1.97720000
39	174.23900000	4.36180000
40	180.79770000	3.86150000
41	183.82970000	5.75100000
42	188.07970000	8.06230000
43	190.91660000	2.88920000
44	205.36320000	14.06320000
45	217.55240000	4.27730000
46	221.07100000	0.68860000
47	228.33220000	5.42420000
48	233.04270000	6.07430000
49	233.81250000	2.90650000

50	241.90190000	2.29600000
51	255.87640000	1.30440000
52	259.39840000	3.45600000
53	262.20630000	3.97380000
54	265.24520000	3.65860000
55	270.56670000	0.77810000
56	284.58670000	1.72990000
57	286.17420000	13.58030000
58	294.53780000	1.04150000
59	296.28650000	0.69870000
60	299.36620000	8.77810000
61	300.28340000	43.47580000
62	303.76430000	40.40100000
63	313.79540000	3.01610000
64	316.32940000	18.61300000
65	322.03810000	18.02330000
66	324.79120000	3.30430000
67	327.26550000	33.33640000
68	337.03580000	47.96780000
69	339.81120000	18.45360000
70	344.44240000	43.02970000
71	345.13610000	6.61910000
72	356.21140000	12.82370000
73	358.13390000	9.69150000
74	364.92100000	56.97590000
75	386.91800000	22.65430000
76	389.28200000	30.09950000
77	396.36290000	29.48960000
78	401.69530000	32.64890000
79	405.07050000	5.54850000
80	413.85200000	0.26830000
81	417.99170000	25.07470000
82	426.52330000	108.36160000
83	432.51310000	13.25480000
84	440.38020000	21.55080000
85	455.24510000	21.41100000
86	461.29820000	6.94060000
87	468.84870000	5.02330000
88	483.28780000	72.43880000
89	495.88220000	0.34340000
90	511.28200000	14.64720000
91	512.61440000	2.74140000
92	521.14510000	38.78960000
93	523.70050000	22.06790000
94	527.21430000	16.49440000
95	535.96460000	50.67490000
96	537.34200000	1.52090000
97	544.29190000	43.66580000
98	582.63820000	5.59210000
99	589.31400000	4.53170000
100	604.04190000	29.63020000
101	667.51160000	53.62380000
102	736.80580000	8.19550000
103	756.98630000	0.61890000
104	763.59030000	15.26370000

105	771.58040000	39.12390000
106	784.54650000	52.12450000
107	789.94160000	40.74990000
108	792.97480000	4.91240000
109	805.56780000	7.03480000
110	810.17680000	35.02580000
111	816.60890000	5.49010000
112	829.14180000	4.53490000
113	837.59470000	7.99160000
114	843.55170000	5.57580000
115	847.60510000	15.03510000
116	851.80360000	12.44490000
117	857.78660000	1.20870000
118	866.06300000	6.17030000
119	870.40270000	23.52640000
120	870.76790000	40.67380000
121	898.15320000	11.30230000
122	902.05610000	53.26420000
123	906.32040000	45.79570000
124	907.33070000	24.64350000
125	909.00040000	8.32990000
126	912.78120000	2.10750000
127	913.06240000	37.98150000
128	915.89760000	9.64070000
129	926.68420000	23.79050000
130	930.12930000	0.47860000
131	936.14360000	1.46090000
132	957.73770000	27.27230000
133	961.22990000	6.56610000
134	973.94210000	0.55880000
135	977.38950000	8.45760000
136	1005.81290000	1.24060000
137	1010.68960000	7.75110000
138	1014.44960000	58.67430000
139	1018.34640000	27.38190000
140	1031.77870000	50.43640000
141	1032.37660000	68.60440000
142	1032.90850000	49.54420000
143	1048.41750000	180.45820000
144	1052.57950000	14.50130000
145	1053.37640000	163.49280000
146	1061.27200000	183.21080000
147	1062.27470000	1.00320000
148	1068.83940000	4.76370000
149	1069.06380000	3.37390000
150	1076.23420000	20.90670000
151	1094.59620000	4.13510000
152	1101.94270000	34.20480000
153	1108.41380000	32.48630000
154	1111.81290000	22.99610000
155	1113.92120000	20.92550000
156	1115.98730000	31.41140000
157	1125.25960000	119.36210000
158	1128.67680000	7.67120000
159	1139.06410000	24.05070000

160	1146.69880000	101.64350000
161	1154.82480000	51.35760000
162	1172.56520000	52.18800000
163	1173.49600000	51.06130000
164	1179.40430000	29.59810000
165	1184.28270000	83.73630000
166	1185.55190000	294.96100000
167	1193.01630000	30.75140000
168	1212.79580000	16.00140000
169	1213.75400000	8.22500000
170	1217.81840000	15.63330000
171	1221.02720000	10.30830000
172	1224.97090000	15.81950000
173	1230.07040000	18.10150000
174	1242.91050000	4.05710000
175	1257.69310000	90.25450000
176	1260.44630000	47.74680000
177	1270.35310000	18.80900000
178	1270.83570000	20.08880000
179	1280.78440000	128.14020000
180	1296.31140000	120.39290000
181	1303.74790000	15.41010000
182	1315.81730000	4.63630000
183	1318.80140000	7.56570000
184	1319.13910000	2.63770000
185	1331.42270000	12.70990000
186	1339.86590000	11.50700000
187	1347.84460000	6.61000000
188	1353.81160000	5.25830000
189	1363.75440000	0.40140000
190	1371.43390000	5.96620000
191	1372.54300000	10.72640000
192	1374.35710000	5.55020000
193	1376.41910000	0.58070000
194	1378.53030000	0.24670000
195	1379.95850000	17.48010000
196	1386.62450000	24.26030000
197	1390.93020000	27.61250000
198	1394.86200000	25.39220000
199	1396.03200000	95.44290000
200	1396.60030000	107.25030000
201	1401.54630000	13.33370000
202	1402.76470000	96.34640000
203	1405.29560000	8.98330000
204	1406.53380000	33.20360000
205	1407.13670000	7.18680000
206	1412.63730000	1.26750000
207	1413.28710000	8.65090000
208	1414.25510000	17.53420000
209	1420.84480000	26.62640000
210	1426.12930000	17.16330000
211	1426.74830000	19.52770000
212	1436.76960000	14.33810000
213	1436.91720000	7.11180000
214	1440.02290000	24.56200000

215	1443.80680000	19.96510000
216	1468.93020000	3.20820000
217	1469.72750000	0.20050000
218	1477.59280000	4.36390000
219	1477.93000000	0.49230000
220	1478.04670000	8.65500000
221	1481.15830000	9.04610000
222	1483.44230000	3.49010000
223	1483.60910000	7.66680000
224	1484.14920000	10.41730000
225	1484.25180000	4.72800000
226	1484.94130000	10.66070000
227	1486.08480000	2.15910000
228	1487.64990000	5.37830000
229	1489.69630000	19.36780000
230	1491.13830000	12.13580000
231	1491.85750000	3.82710000
232	1492.87420000	2.57700000
233	1493.27230000	4.50280000
234	1493.81260000	6.48980000
235	1497.22090000	12.81180000
236	1497.76100000	2.10940000
237	1498.21910000	0.83540000
238	1499.19160000	2.61960000
239	1502.04690000	3.43260000
240	1505.19880000	11.75580000
241	1505.65380000	9.67210000
242	1507.16460000	10.91600000
243	1508.82380000	8.47740000
244	1510.84640000	3.19140000
245	1512.20430000	9.59800000
246	1515.66200000	4.46140000
247	1516.17650000	4.15080000
248	1517.96630000	14.25090000
249	1518.74100000	6.40740000
250	1523.52530000	6.47380000
251	1524.92190000	18.41530000
252	1525.31320000	46.30040000
253	1529.88830000	13.44440000
254	1611.44900000	6.58380000
255	1713.65400000	569.30330000
256	2266.17620000	744.81130000
257	2989.01090000	51.83120000
258	2995.58250000	29.84850000
259	2997.33670000	62.01030000
260	3010.09430000	40.08540000
261	3011.43070000	55.81470000
262	3013.46650000	47.71640000
263	3021.06850000	33.59580000
264	3021.31290000	47.76310000
265	3024.46070000	77.24730000
266	3025.69850000	9.28410000
267	3028.88140000	94.67990000
268	3032.26980000	25.20870000
269	3034.11610000	95.39580000

270	3036.46110000	103.13430000
271	3038.51230000	17.41520000
272	3038.61150000	27.45020000
273	3040.90020000	11.58190000
274	3041.52400000	20.82620000
275	3042.99940000	7.09860000
276	3045.17450000	16.29680000
277	3047.87980000	17.54570000
278	3048.81080000	35.21720000
279	3049.14560000	31.34170000
280	3049.26440000	112.21870000
281	3054.47400000	30.38750000
282	3059.66130000	5.51240000
283	3061.86620000	51.47490000
284	3066.89510000	44.61330000
285	3067.72440000	29.36430000
286	3069.13320000	49.26200000
287	3075.37370000	31.40920000
288	3077.58980000	78.79580000
289	3079.33240000	36.05340000
290	3082.31740000	90.32430000
291	3082.99080000	28.78130000
292	3084.07490000	21.16650000
293	3084.51400000	48.41410000
294	3089.25650000	84.08730000
295	3098.03840000	20.20970000
296	3098.31190000	39.88300000
297	3102.17660000	18.43730000
298	3103.98980000	33.33060000
299	3104.16940000	37.96350000
300	3104.81130000	49.48200000
301	3106.46640000	9.78680000
302	3107.11250000	26.55350000
303	3110.66070000	55.24900000
304	3111.37510000	12.07130000
305	3111.55000000	11.22470000
306	3113.72210000	21.91960000
307	3116.76380000	34.67860000
308	3118.47690000	21.44140000
309	3119.94990000	28.32880000
310	3124.26900000	29.95950000
311	3128.15540000	50.70410000
312	3130.16590000	42.92180000
313	3130.66590000	15.36590000
314	3140.09290000	22.44450000
315	3141.92820000	10.04470000
316	3145.12270000	26.13660000
317	3146.96760000	9.81300000
318	3149.28890000	13.93910000
319	3149.62680000	9.10350000
320	3157.21740000	11.22190000
321	3164.67560000	5.32460000

6. HPLC/GC traces

tert-Butyl 2-Cyano-2-(2-phenylsulfanyl)piperidin-1-carboxylate 5a

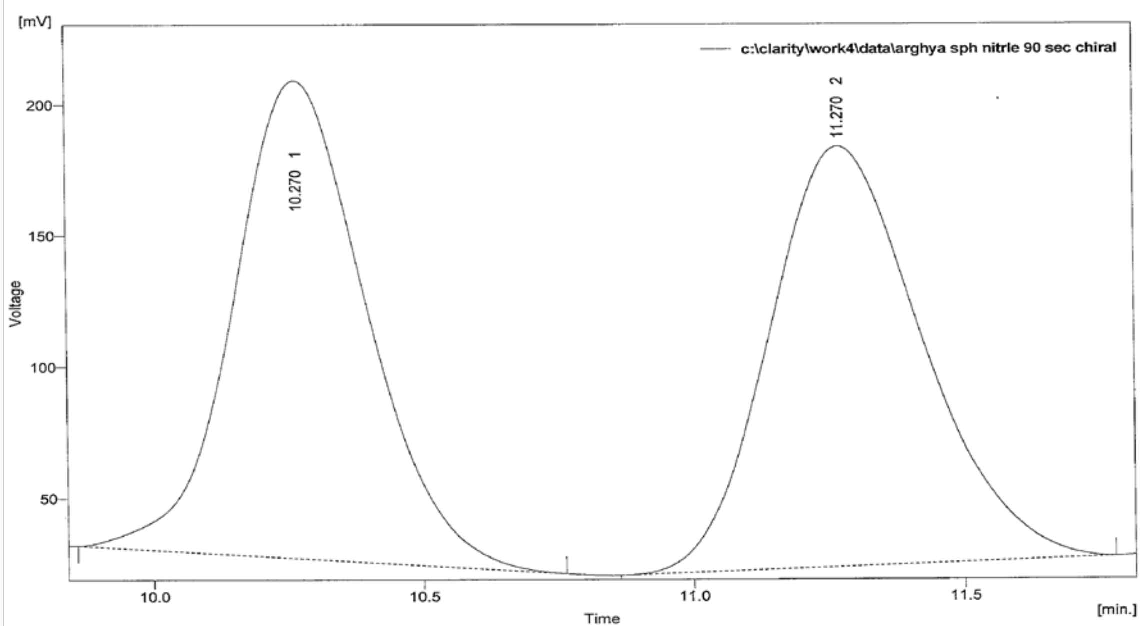

Result Table - Calculation Method Uncal

	Reten. Time [min]	Area [mV.s]	Height [mV]	Area [%]	Height [%]	W05 [min]	Response Factor
1	10.270	3104.899	182.312	49.8	53.2	0.26	0.0
2	11.270	3129.136	160.583	50.2	46.8	0.31	0.0
	Total	6234.035	342.895	100.0	100.0		-

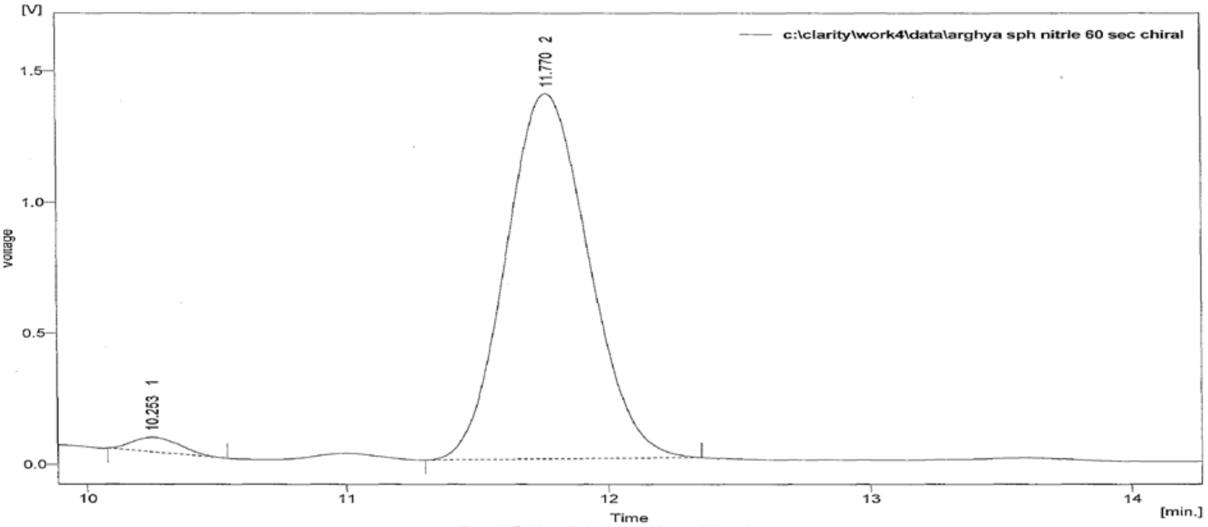

Result Table - Calculation Method Uncal

	Reten. Time [min]	Area [mV.s]	Height [mV]	Area [%]	Height [%]	W05 [min]	Response Factor
1	10.253	716.416	55.254	2.3	3.8	0.21	0.0
2	11.770	30561.612	1392.200	97.7	96.2	0.35	0.0
	Total	31278.028	1447.454	100.0	100.0		-

tert-Butyl 2-Cyano-2-(*p*-tolylsulfanyl)piperidin-1-carboxylate 5b

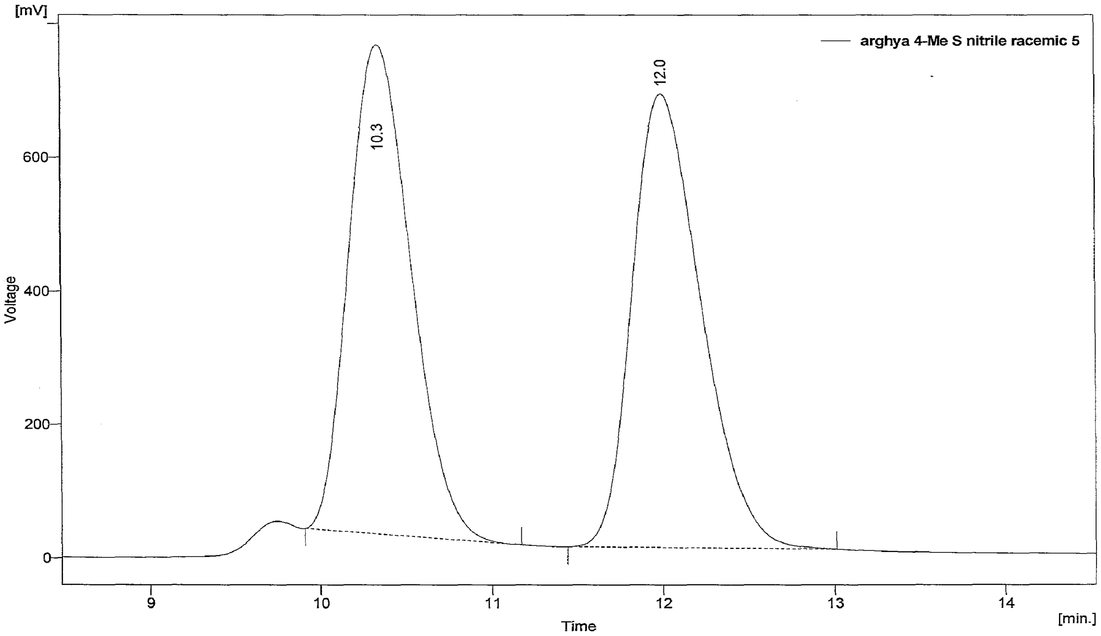

Result Table - Calculation Method Uncal

	Reten. Time [min]	Area [mV.s]	Height [mV]	Area [%]	Height [%]	W05 [min]
1	10.337	18430.202	732.364	49.2	51.9	0.40
2	11.993	19007.543	678.906	50.8	48.1	0.44
	Total	37437.745	1411.269	100.0	100.0	

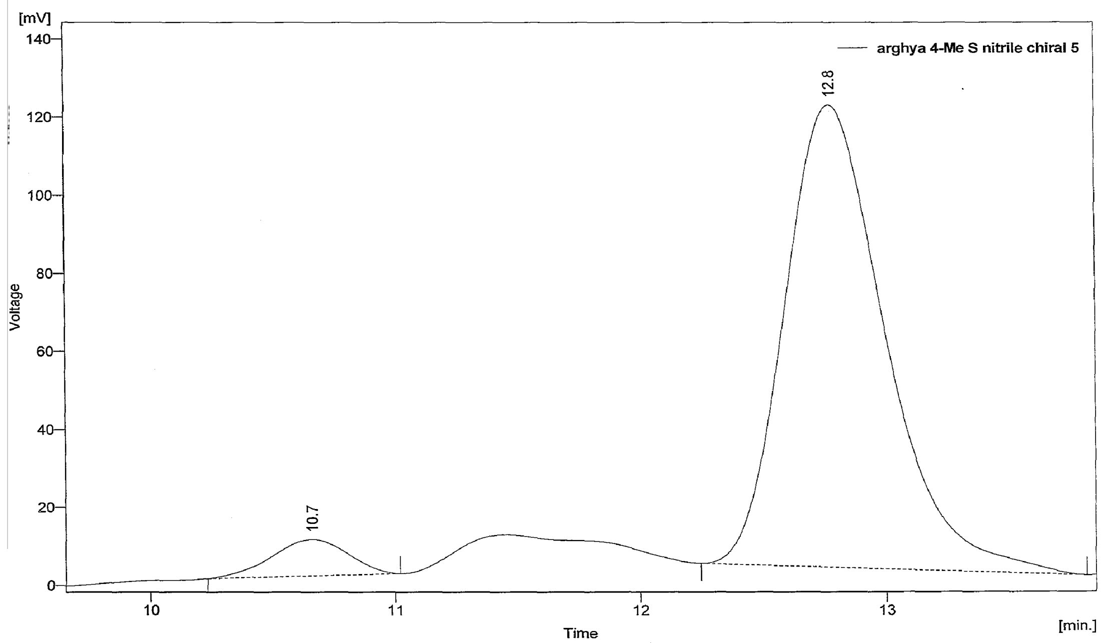

Result Table - Calculation Method Uncal

	Reten. Time [min]	Area [mV.s]	Height [mV]	Area [%]	Height [%]	W05 [min]
1	10.663	199.629	9.298	5.6	7.3	0.35
2	12.773	3348.863	118.515	94.4	92.7	0.43
	Total	3548.492	127.813	100.0	100.0	

tert-Butyl 2-Cyano-2-(_o_-methoxyphenylsulfanyl)piperidin-1-carboxylate 5c

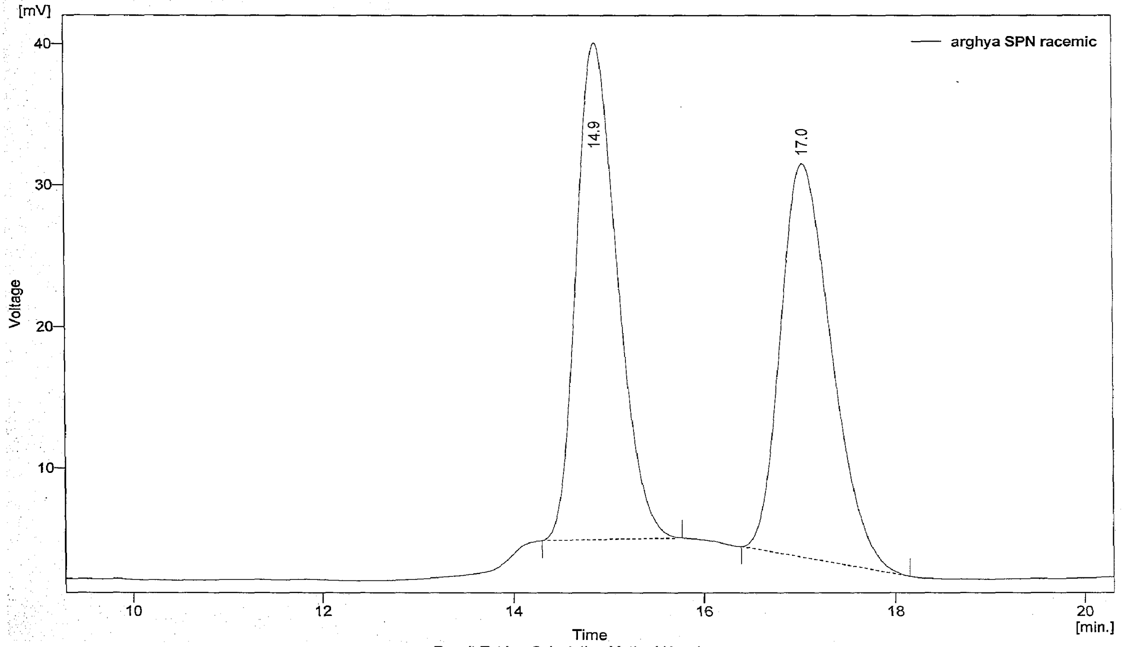

Result Table - Calculation Method Uncal

	Reten. Time [min]	Area [mV.s]	Height [mV]	Area [%]	Height [%]	W05 [min]
1	14.870	1051.692	35.167	50.5	55.8	0.47
2	17.040	1029.016	27.883	49.5	44.2	0.57
	Total	2080.708	63.050	100.0	100.0	

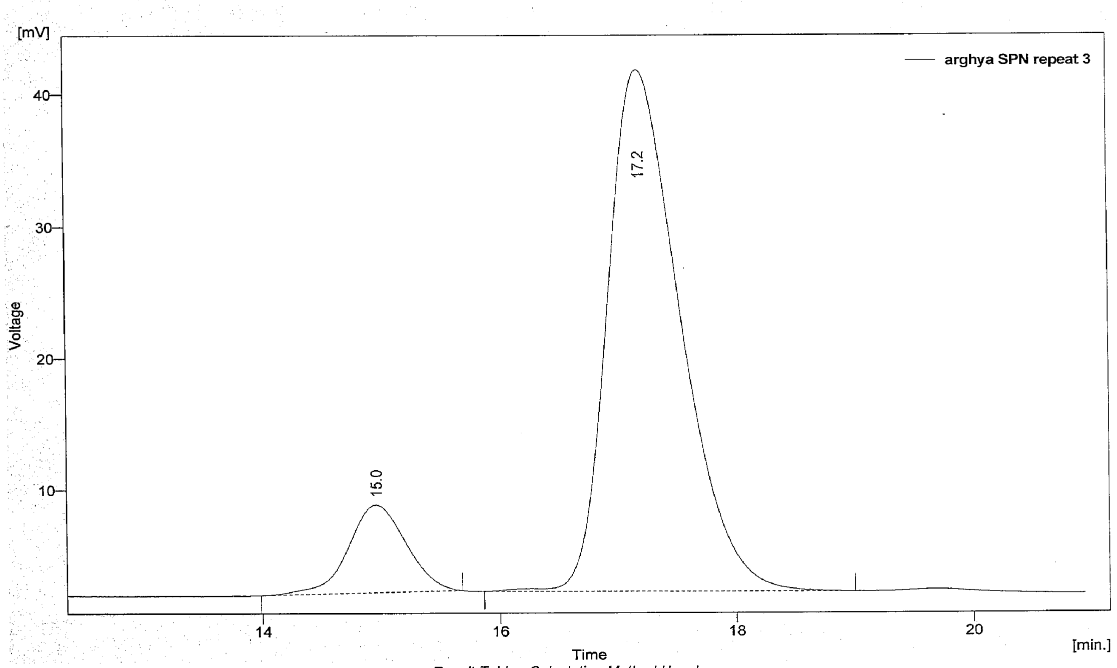

Result Table - Calculation Method Uncal

	Reten. Time [min]	Area [mV.s]	Height [mV]	Area [%]	Height [%]	W05 [min]
1	14.963	225.980	6.643	11.8	14.4	0.52
2	17.200	1685.058	39.582	88.2	85.6	0.66
	Total	1911.038	46.225	100.0	100.0	

tert-Butyl 2-Cyano-2-[hydroxy(phenyl)methyl]piperidine-1-carboxylate 5d

Diastereomer A

06/06/2016 10:28 Chromatogram c:\clarity\work4-2014-2015\arghyabennit rac 1st spt Page 1 of 1

Created	: 20/03/2015 16:33:27	By	: Clarity
Project	: WORK1	Report Style	: Chromatogram
ISTD Amount	: 0	Inj. Volume	: 10
Sample ID	: arghyabennit rac 1st spt	Sample	: arghyabennit rac 1st spt
Calibration	: arghyabennit rac 1st spt	Chromatogram	: c:\clarity\work4-2014-2015\arghyabennit rac 1st spt

Method	: Cellulose-2	By	: ch1rjh
Description	: arghyabennit rac 1st spt		
Created	: 13/03/2001 08:37	Modified	: 20/03/2015 17:33

Column	: Cellulose-1	Detection	: UV/Vis at 220nm
Mobile Phase	: 10% IPA in hexane	Temperature	: RT
Flow Rate	: 1mL/min	Pressure	:
Note	:		

Autostop	: None	External Start	: Start Only, Down
Detector 1	: Signal 1	Range 1	: Bipolar, 1250 mV, 10 Samp. per Sec.
Subtraction chromatogram	: (None)	Matching	: No Change

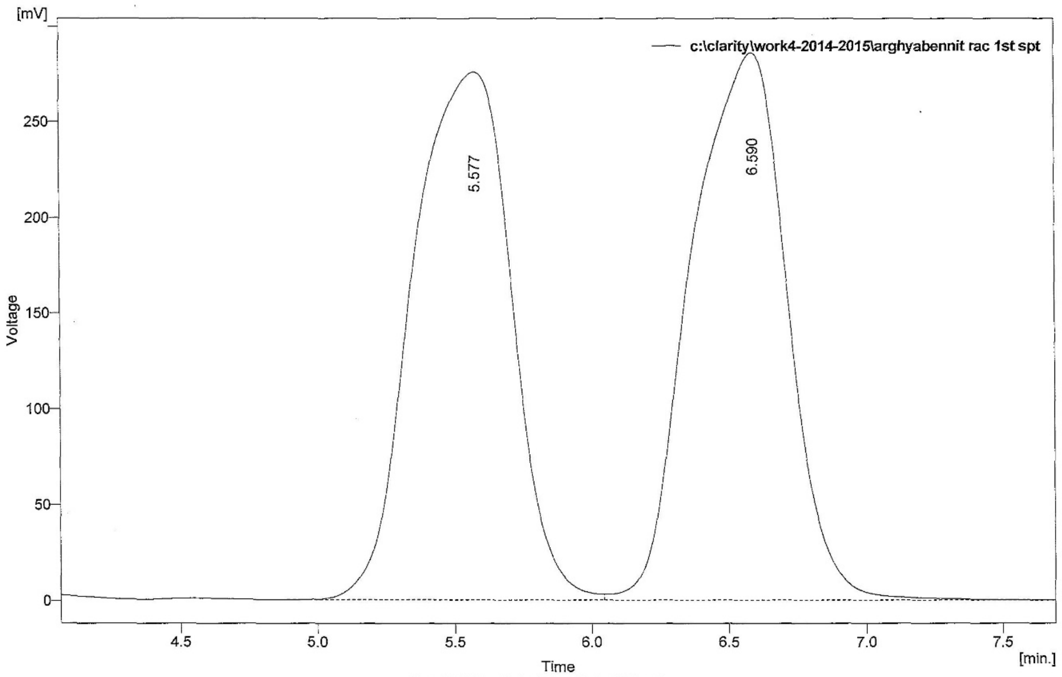

Result Table - Calculation Method Uncal

	Reten. Time [min]	Area [mV.s]	Height [mV]	Area [%]	Height [%]	W05 [min]	Response Factor
1	5.577	6866.274	275.830	49.9	49.1	0.41	0.0
2	6.590	6891.348	286.154	50.1	50.9	0.40	0.0
	Total	13757.622	561.984	100.0	100.0		-

Diastereomer B

Created	: 20/03/2015 17:00:25	By	: Clarity
Project	: WORK1	Report Style	: Chromatogram
ISTD Amount	: 0	Inj. Volume	: 10
Sample ID	: arghyabennit rac 2ndspt2	Sample	: arghyabennit rac 2ndspt2
Calibration	: arghyabennit rac 2ndspt2	Chromatogram	: c:\clarity\work4-2014-2015\arghyabennit rac 2ndspt2
Method	: Cellulose-2	By	: ch1rjh
Description	: arghyabennit rac 2ndspt2		
Created	: 13/03/2001 08:37	Modified	: 20/03/2015 18:00
Column	: Cellulose-1	Detection	: UV/Vis at 220nm
Mobile Phase	: 10% IPA in hexane	Temperature	: RT
Flow Rate	: 1mL/min	Pressure	:
Note	:		
Autostop	: None	External Start	: Start Only, Down
Detector 1	: Signal 1	Range 1	: Bipolar, 1250 mV, 10 Samp. per Sec.
Subtraction chromatogram	: (None)	Matching	: No Change

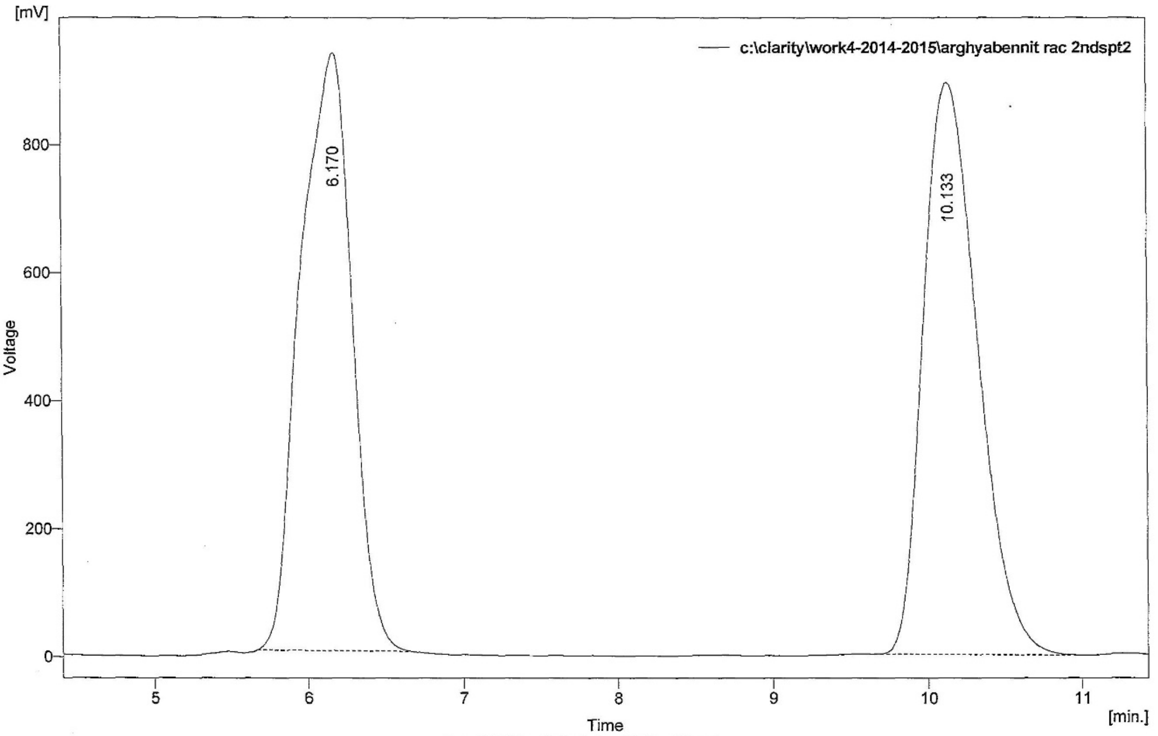

Result Table - Calculation Method Uncal

	Reten. Time [min]	Area [mV.s]	Height [mV]	Area [%]	Height [%]	W05 [min]	Response Factor
1	6.170	21575.307	934.599	49.6	51.1	0.39	0.0
2	10.133	21927.637	893.946	50.4	48.9	0.39	0.0
	Total	43502.944	1828.545	100.0	100.0		-

***tert*-Butyl 2-Cyano-2-(2-hydroxypropan-2-yl)piperidin-1-carboxylate 5e**

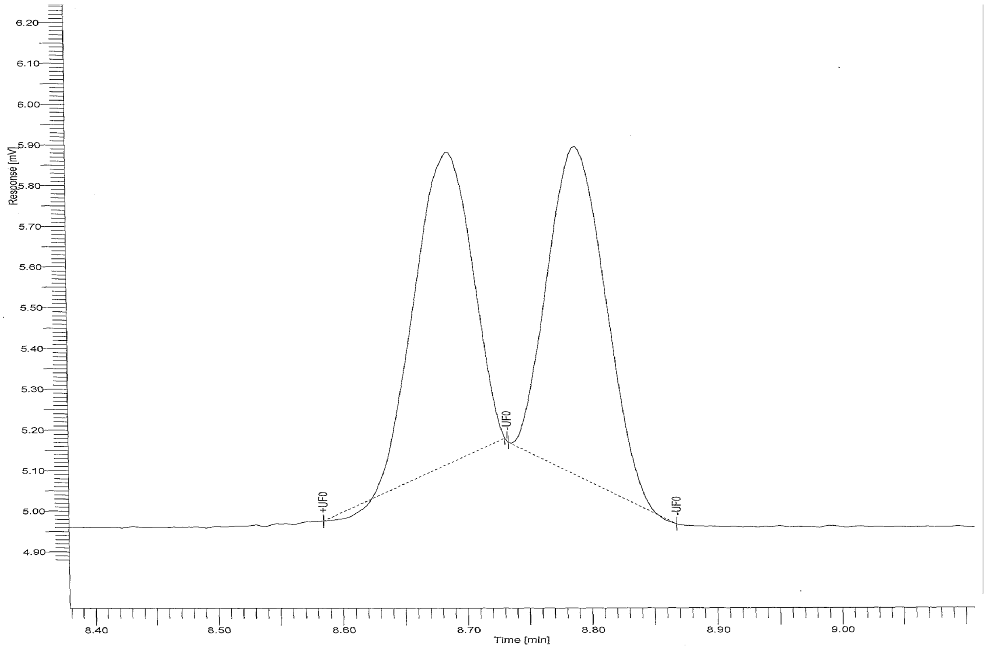

Peak #	Time [min]	Area [μV·s]	Height [μV]	Area [%]	Norm. Area [%]	BL	Area/Height [s]
1	8.685	2490.88	764.57	48.92	48.92	*MM	3.2579
2	8.789	2600.85	810.08	51.08	51.08	*MM	3.2106
		5091.73	1574.66	100.00	100.00		

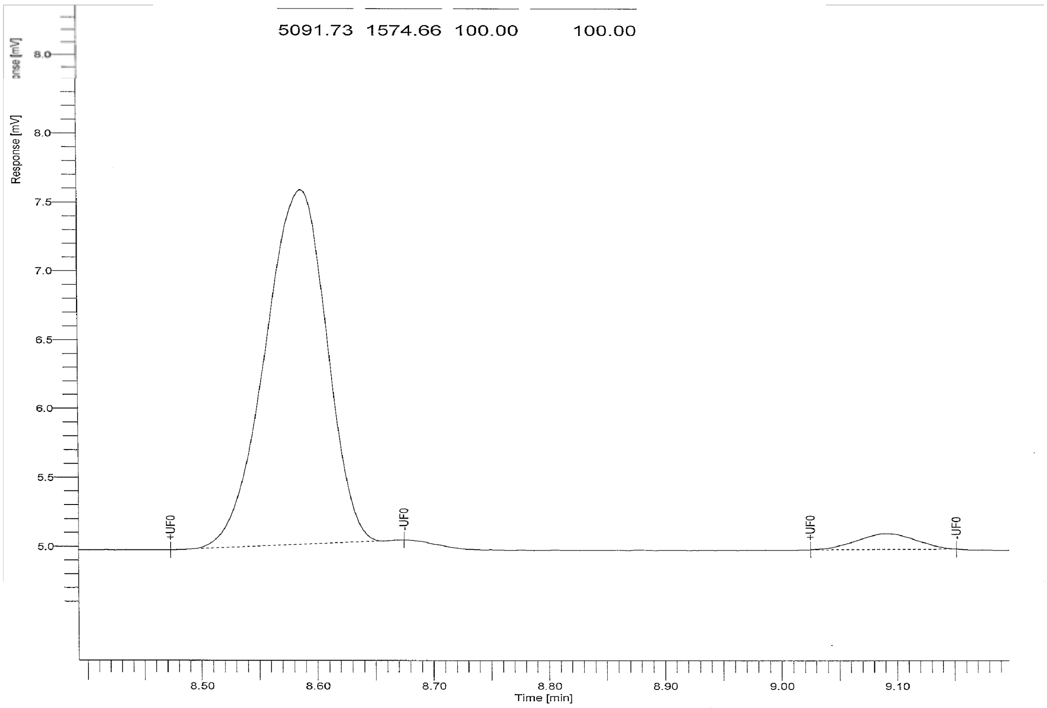

Peak #	Time [min]	Area [μV·s]	Height [μV]	Area [%]	Norm. Area [%]	BL	Area/Height [s]
1	8.587	9476.01	2575.19	95.93	95.93	*MM	3.6797
2	9.091	401.54	113.21	4.07	4.07	*MM	3.5470
		9877.56	2688.40	100.00	100.00		

tert-butyl 2-cyano-2-(1-hydroxycyclobutyl)piperidine-1-carboxylate 5f

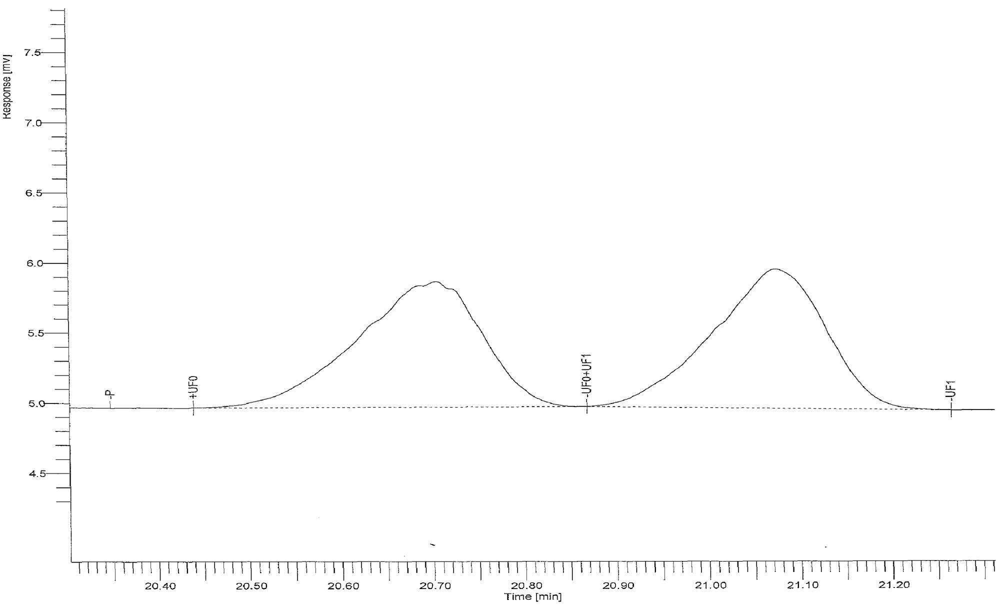

Peak #	Time [min]	Area [µV·s]	Height [µV]	Area [%]	Norm. Area [%]	BL	Area/Height [s]
1	20.704	8506.01	895.35	49.97	49.97	*MM	9.5002
2	21.075	8517.69	994.40	50.03	50.03	*MM	8.5657
		17023.71	1889.75	100.00	100.00		

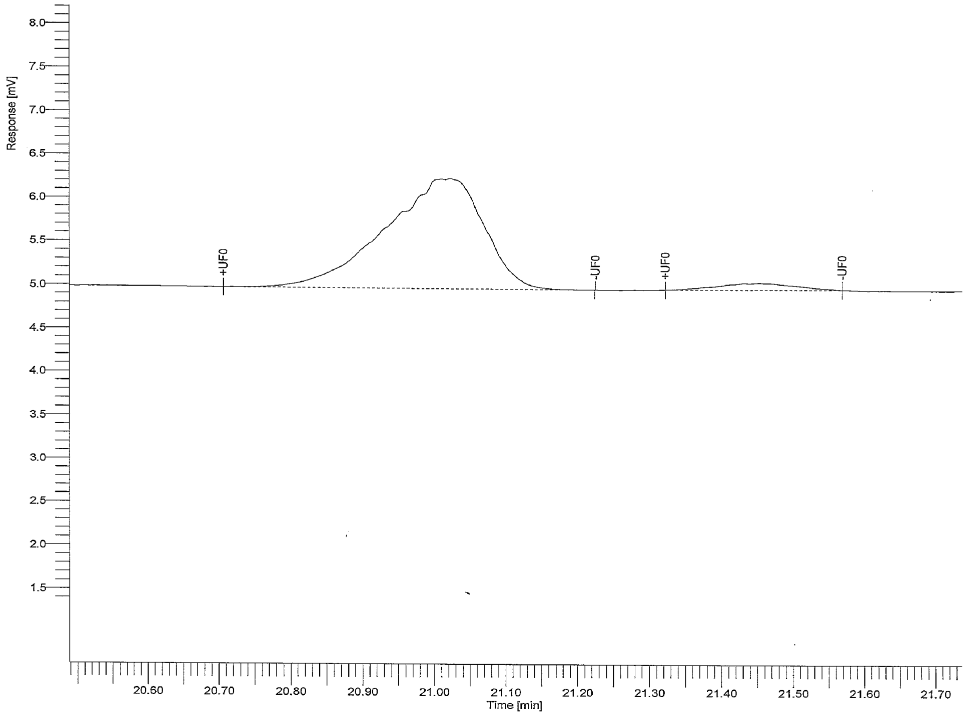

Peak #	Component Name	Time [min]	Area [uV*sec]	Height [uV]	Area [%]	Norm. Area [%]	Cal. Range	Volt Range	BL	Raw Amount
1		21.021	12045.92	1264.81	95.41	95.41		+	*MM	---------
2		21.451	579.54	77.21	4.59	4.59			*MM	0.0006
			12625.46	1342.02	100.00	100.00				0.0006

tert-Butyl 2-(4-Bromobenzoyl)-2-cyanopiperidin-1-carboxylate 5g

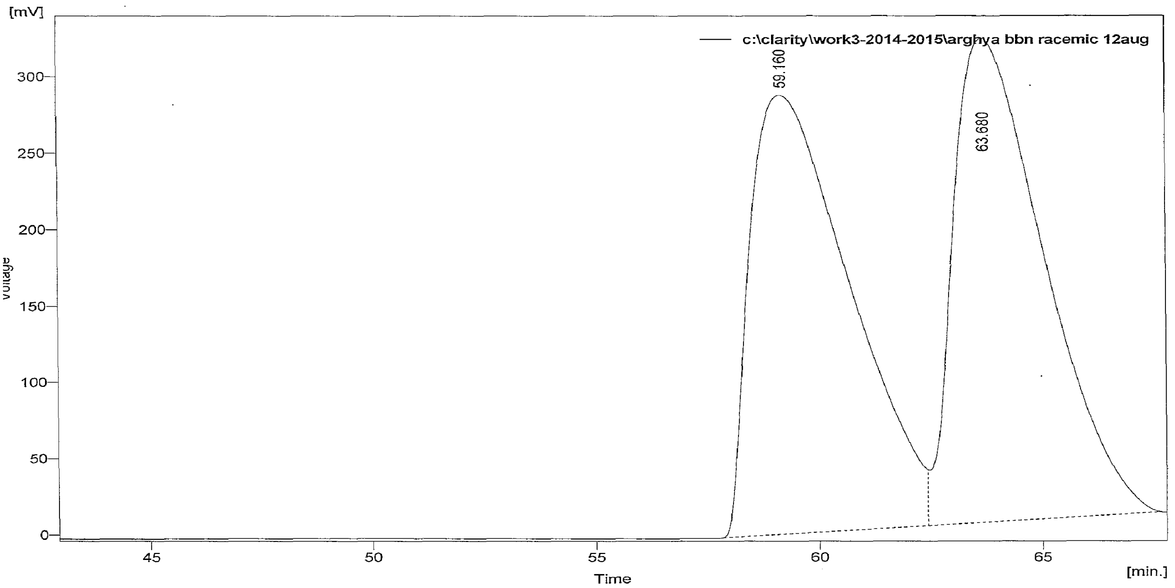

Result Table - Calculation Method Uncal

	Reten. Time [min]	Area [mV.s]	Height [mV]	Area [%]	Height [%]	W05 [min]	Response Factor
1	59.160	44396.607	287.480	49.6	47.5	2.56	0.0
2	63.680	45042.678	317.305	50.4	52.5	2.27	0.0
	Total	89439.285	604.785	100.0	100.0		-

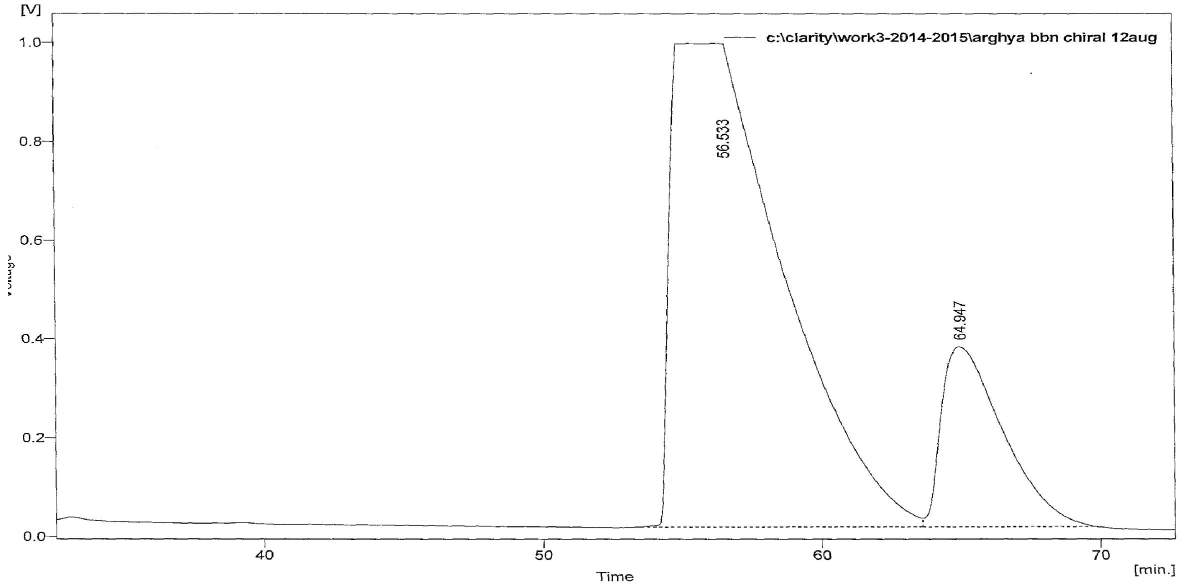

Result Table - Calculation Method Uncal

	Reten. Time [min]	Area [mV.s]	Height [mV]	Area [%]	Height [%]	W05 [min]	Response Factor
1	56.533	271529.967	979.024	82.9	72.9	4.31	0.0
2	64.947	56169.594	363.539	17.1	27.1	2.41	0.0
	Total	327699.560	1342.563	100.0	100.0		-

7. $^{1}H/^{13}C$ NMR spectra

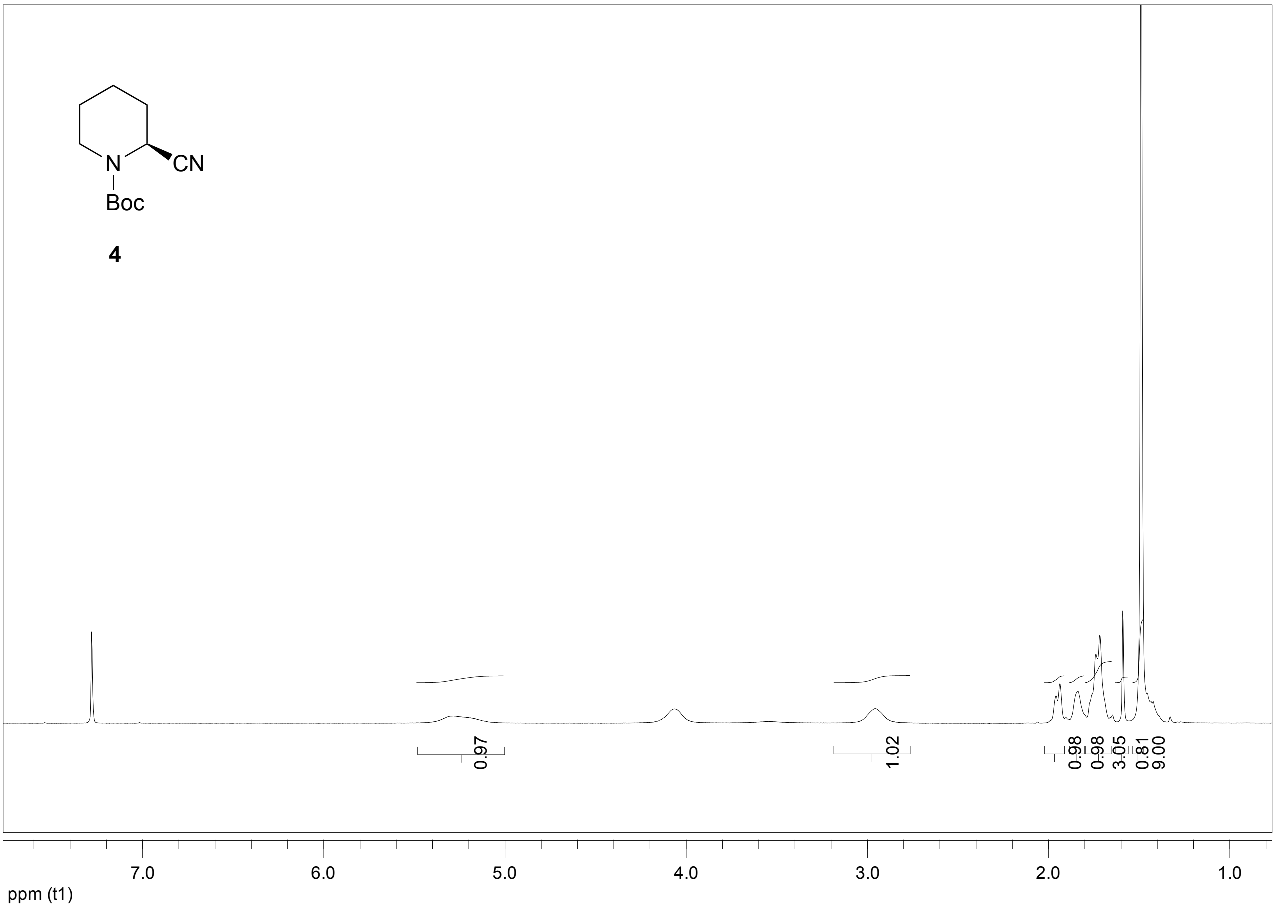

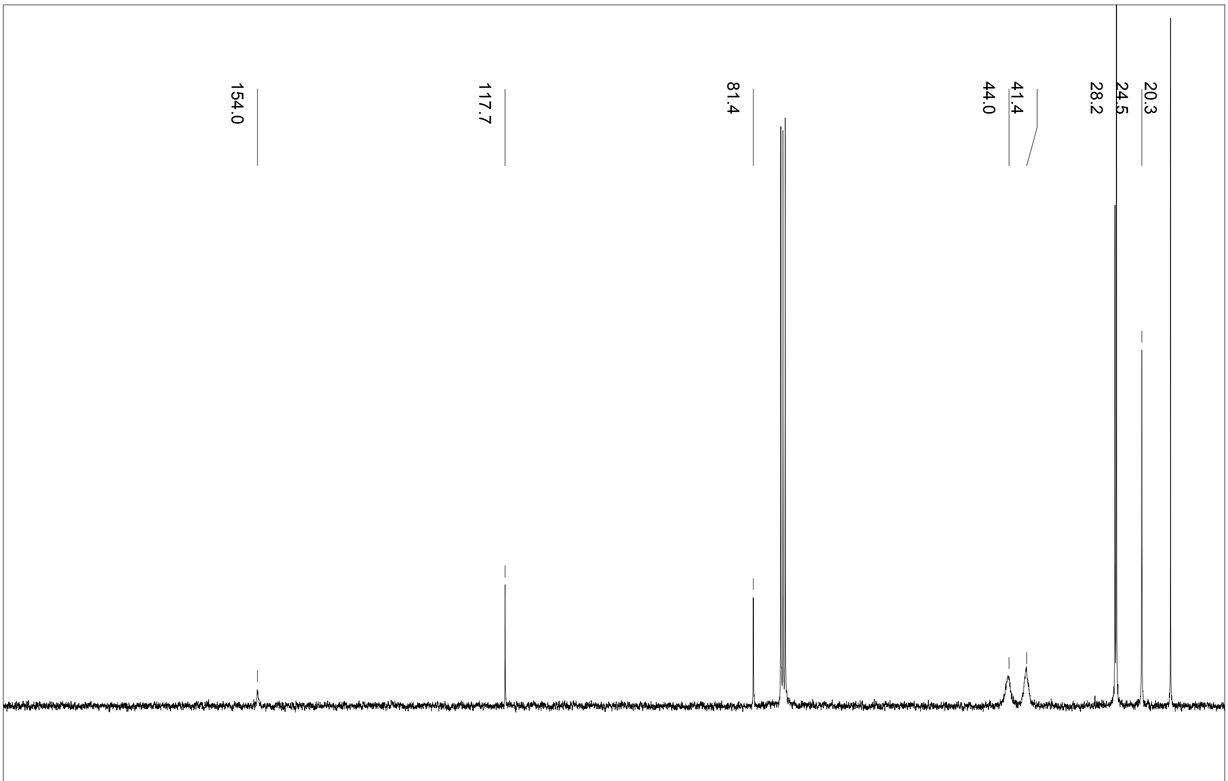

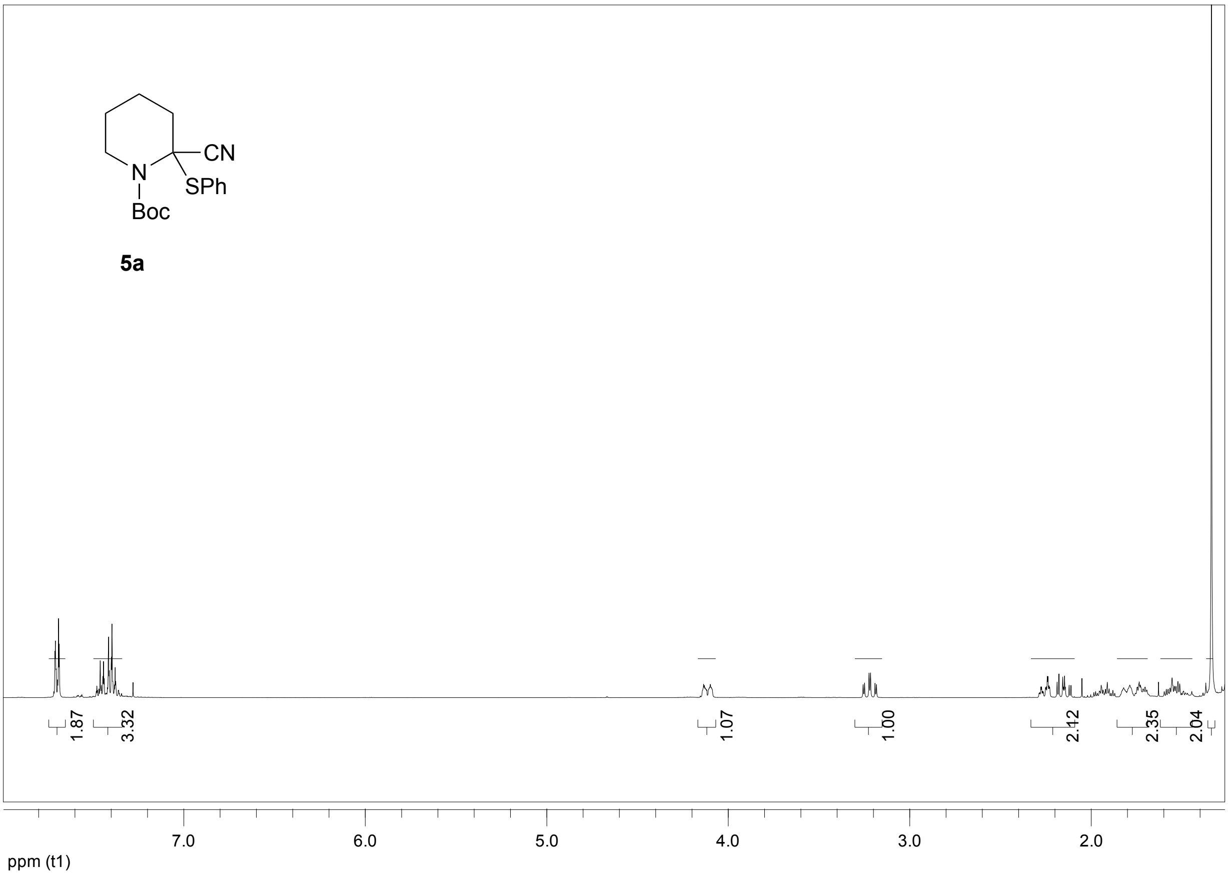
CN
N
SPh
Boc
5a
1.87
3.32
1.07
1.00
2.12
2.35
2.04
7.0
6.0
5.0
4.0
3.0
2.0
ppm (t1)

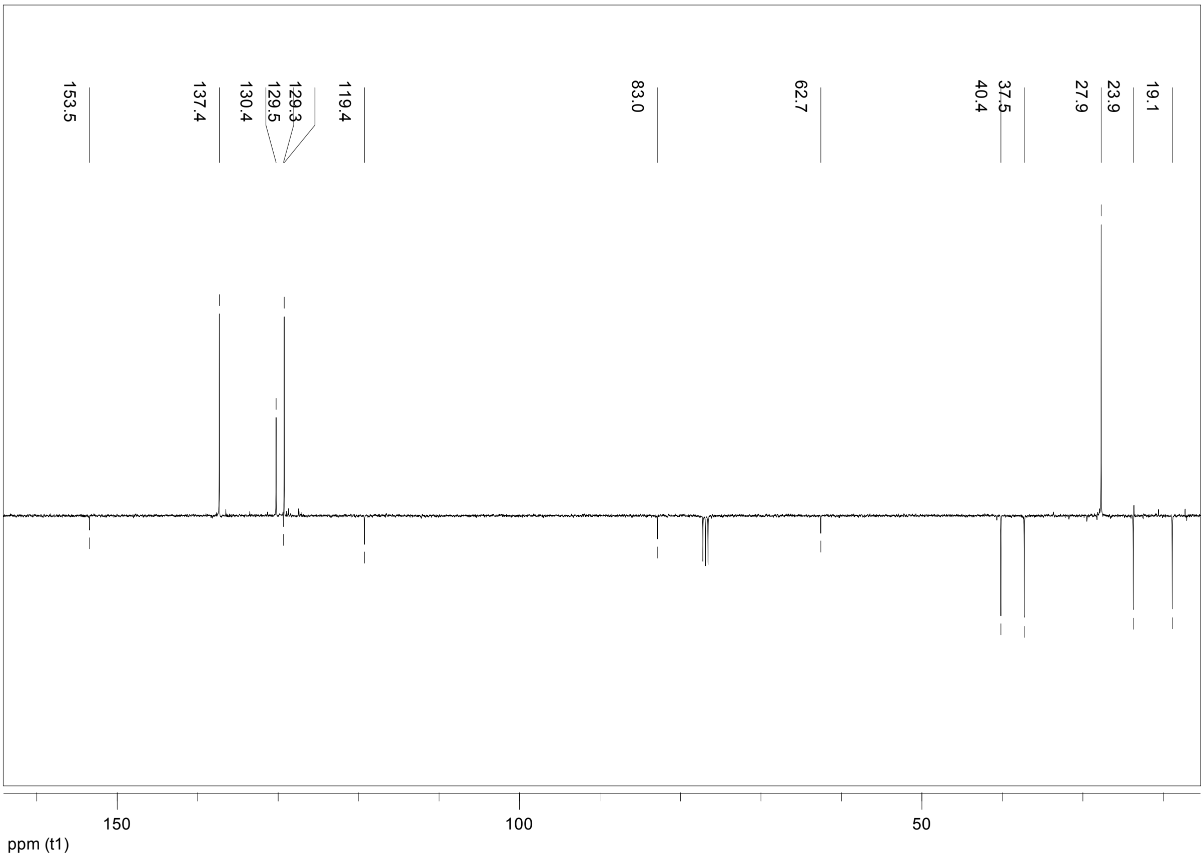
153.5
137.4
130.4
129.5
129.3
119.4
83.0
62.7
40.4
37.5
27.9
23.9
19.1
150
100
50
ppm (t1)

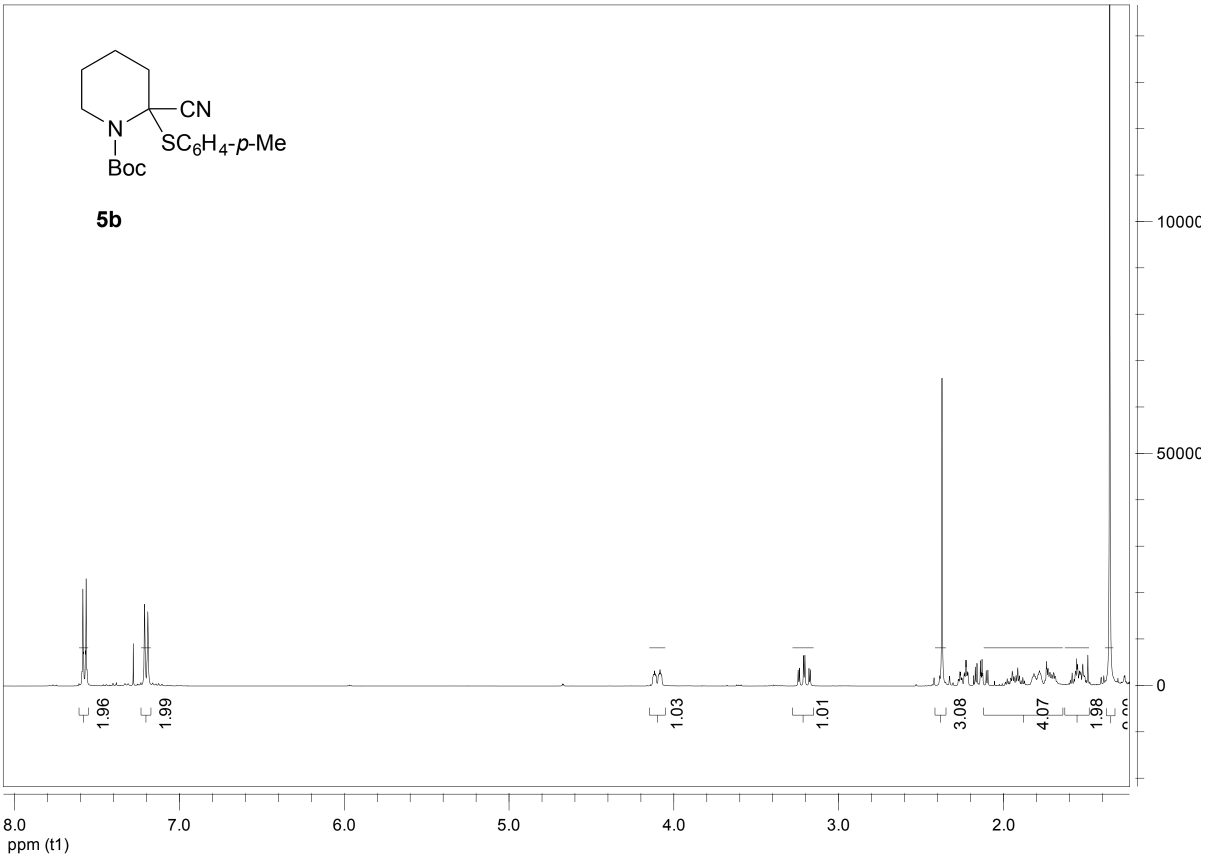
CN
N
SC6H4-p-Me
Boc
5b
1.96
1.99
1.03
1.01
3.08
4.07
1.98
10000
50000
0
8.0
7.0
6.0
5.0
4.0
3.0
2.0
ppm (t1)

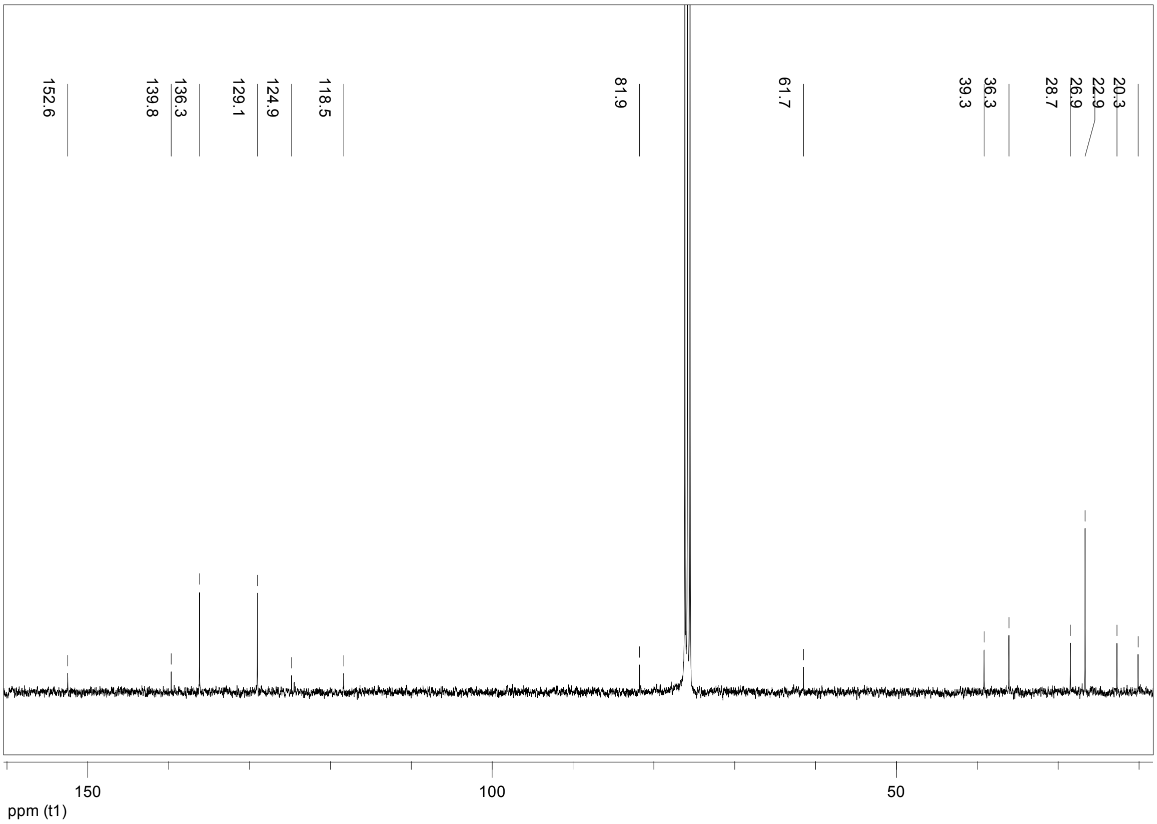
152.6
139.8
136.3
129.1
124.9
118.5
81.9
61.7
39.3
36.3
28.7
26.9
22.9
20.3
150
100
50
ppm (t1)

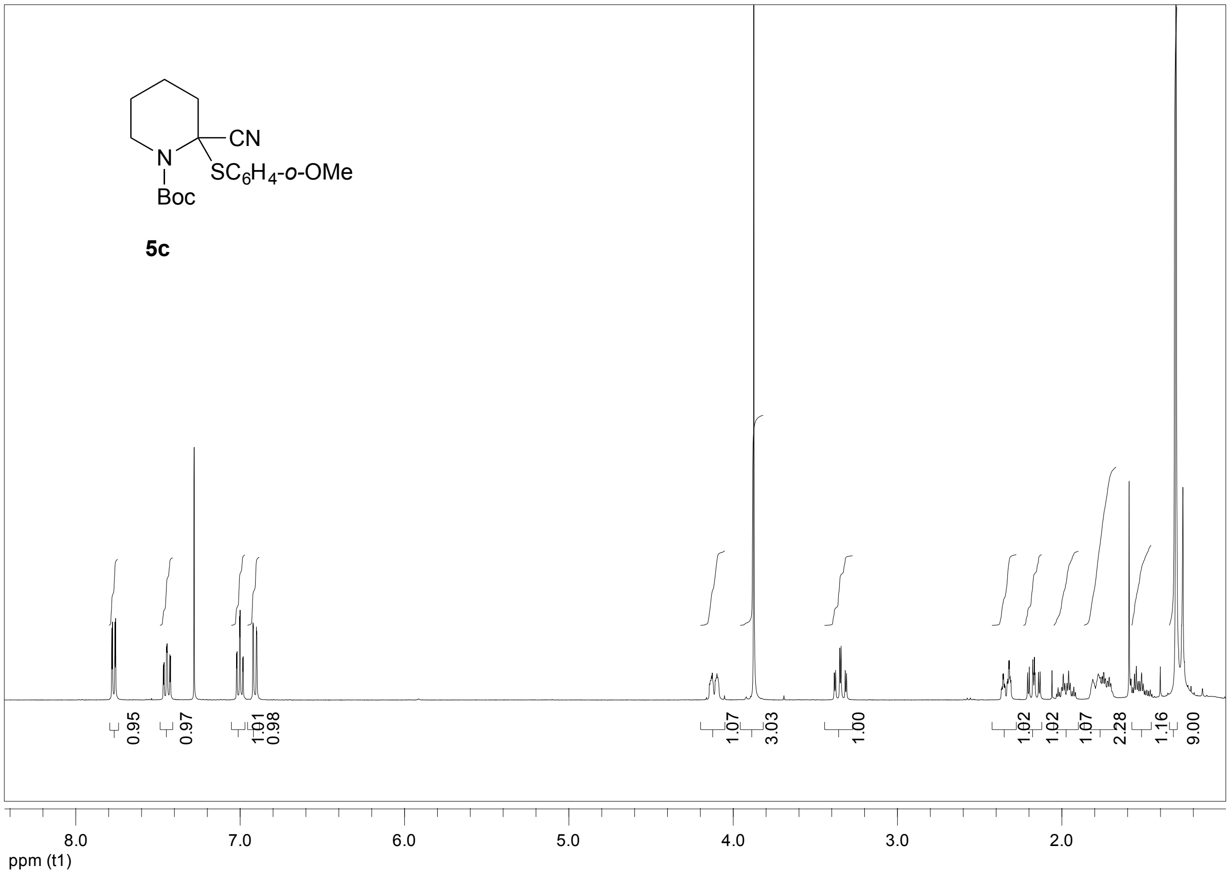
CN
N
SC6H4-o-OMe
Boc
5c
0.95
0.97
1.01
0.98
1.07
3.03
1.00
1.02
1.02
1.07
2.28
1.16
9.00
8.0
7.0
6.0
5.0
4.0
3.0
2.0
ppm (t1)

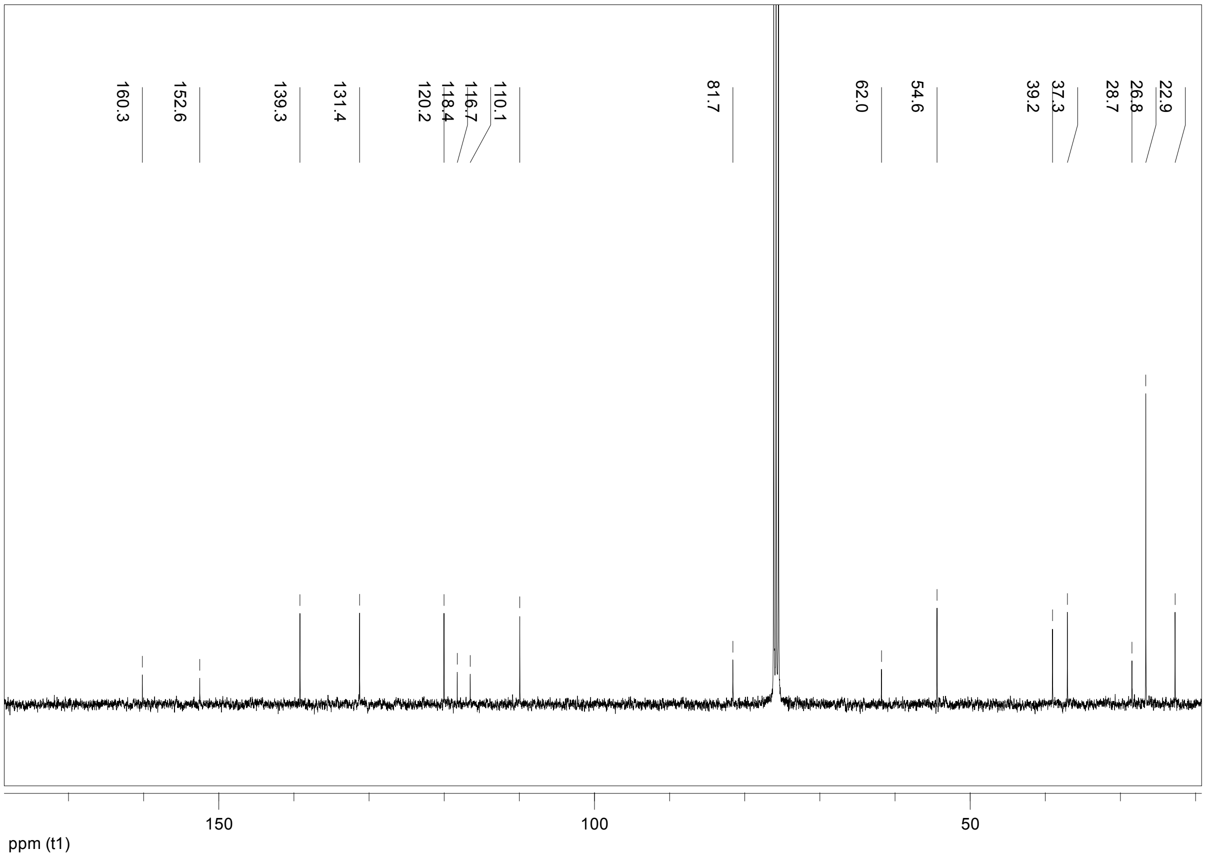
160.3
152.6
139.3
131.4
120.2
118.4
116.7
110.1
81.7
62.0
54.6
39.2
37.3
28.7
26.8
22.9
150
100
50
ppm (t1)

Diastereomer A

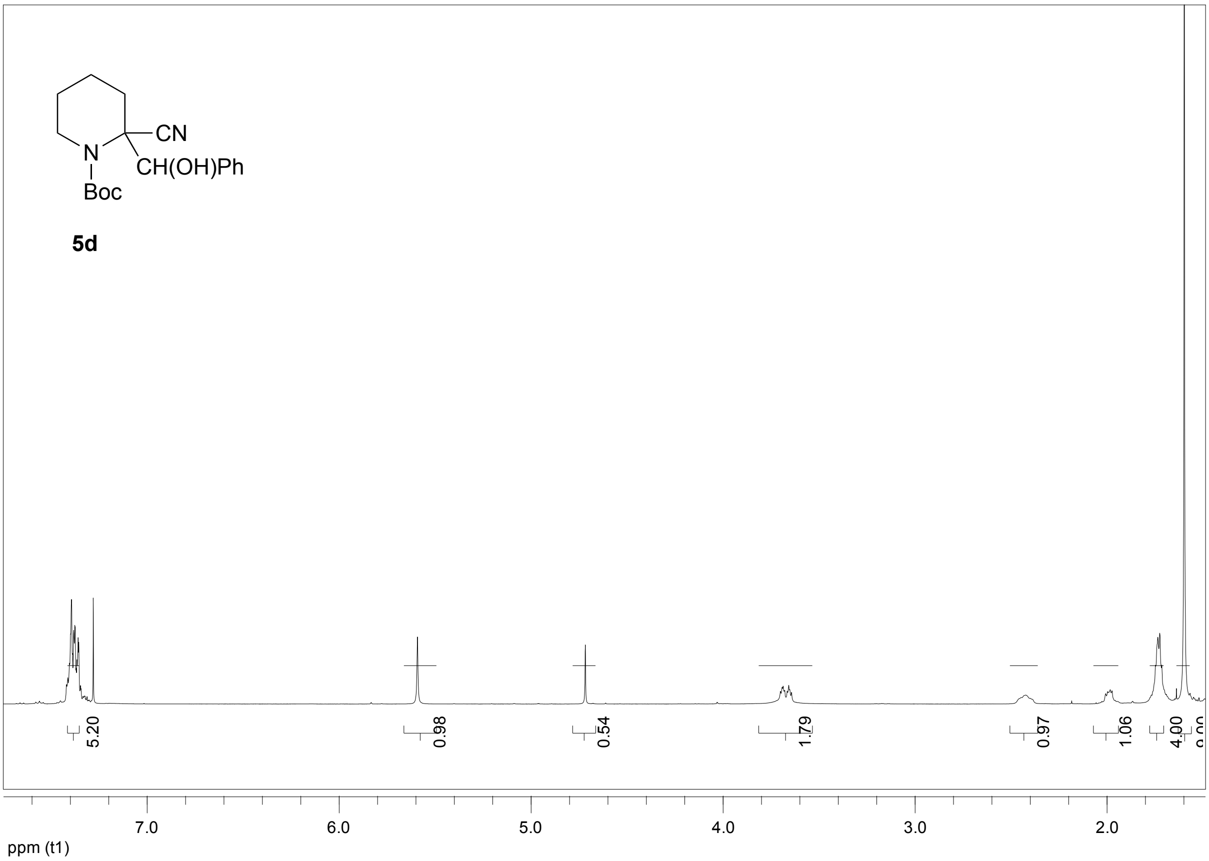

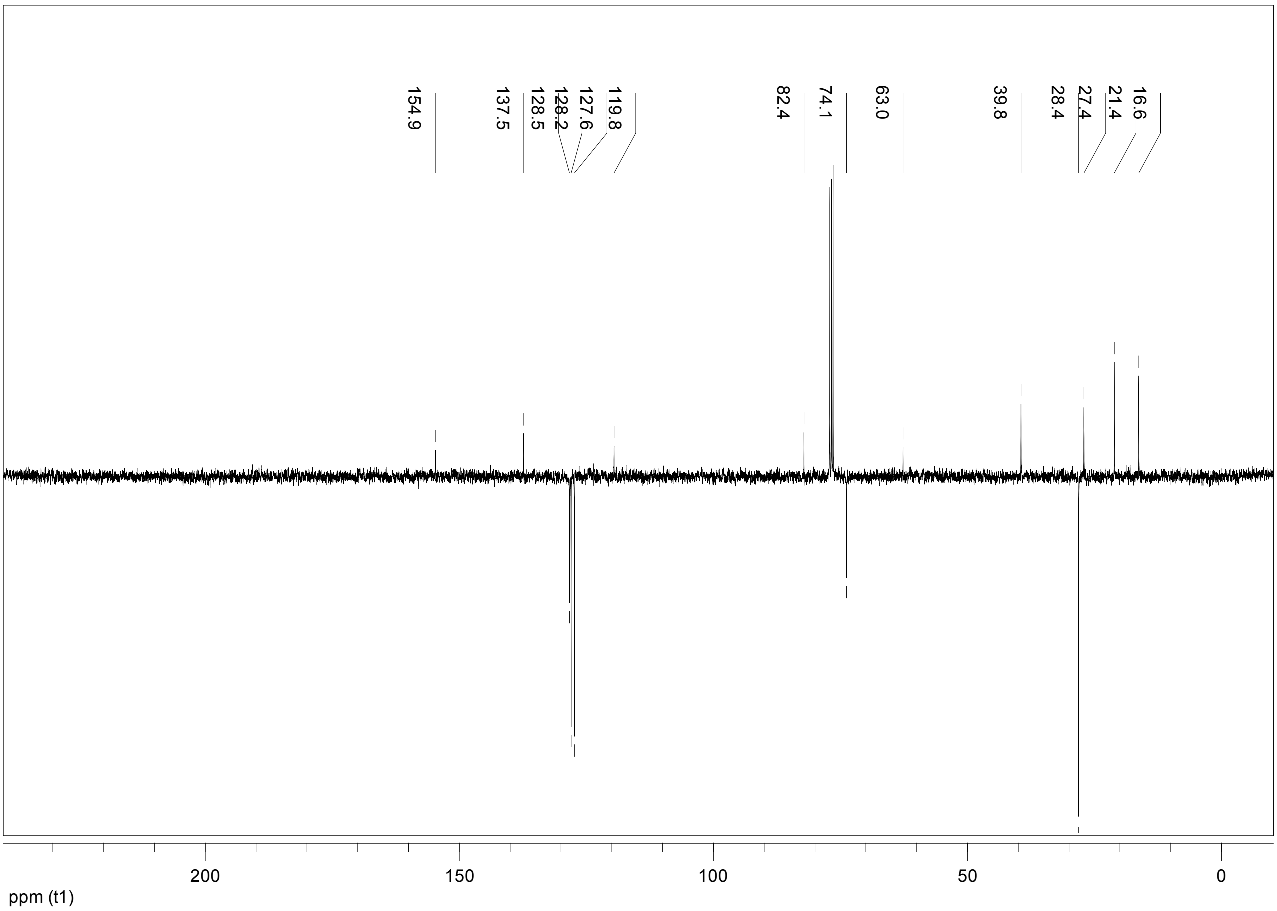

Diastereomer B

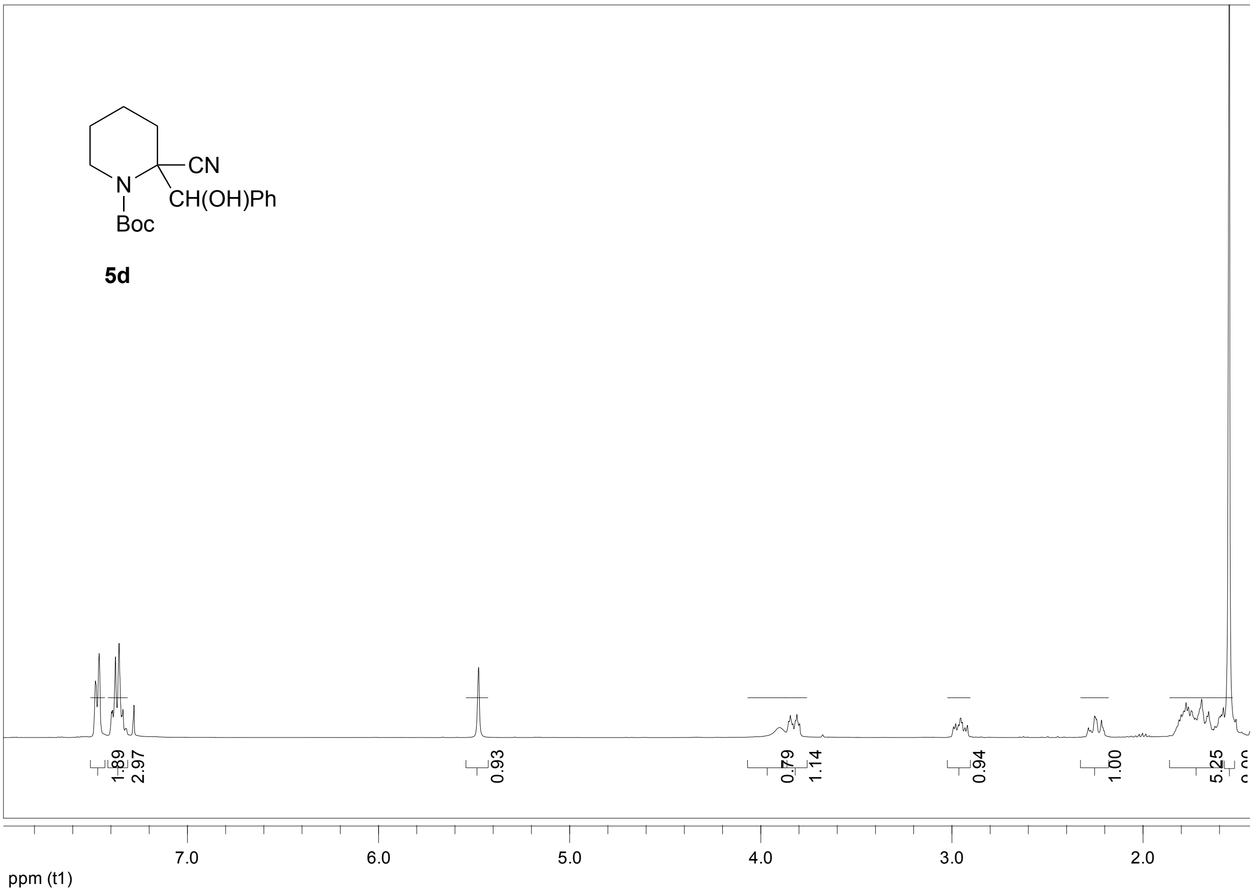

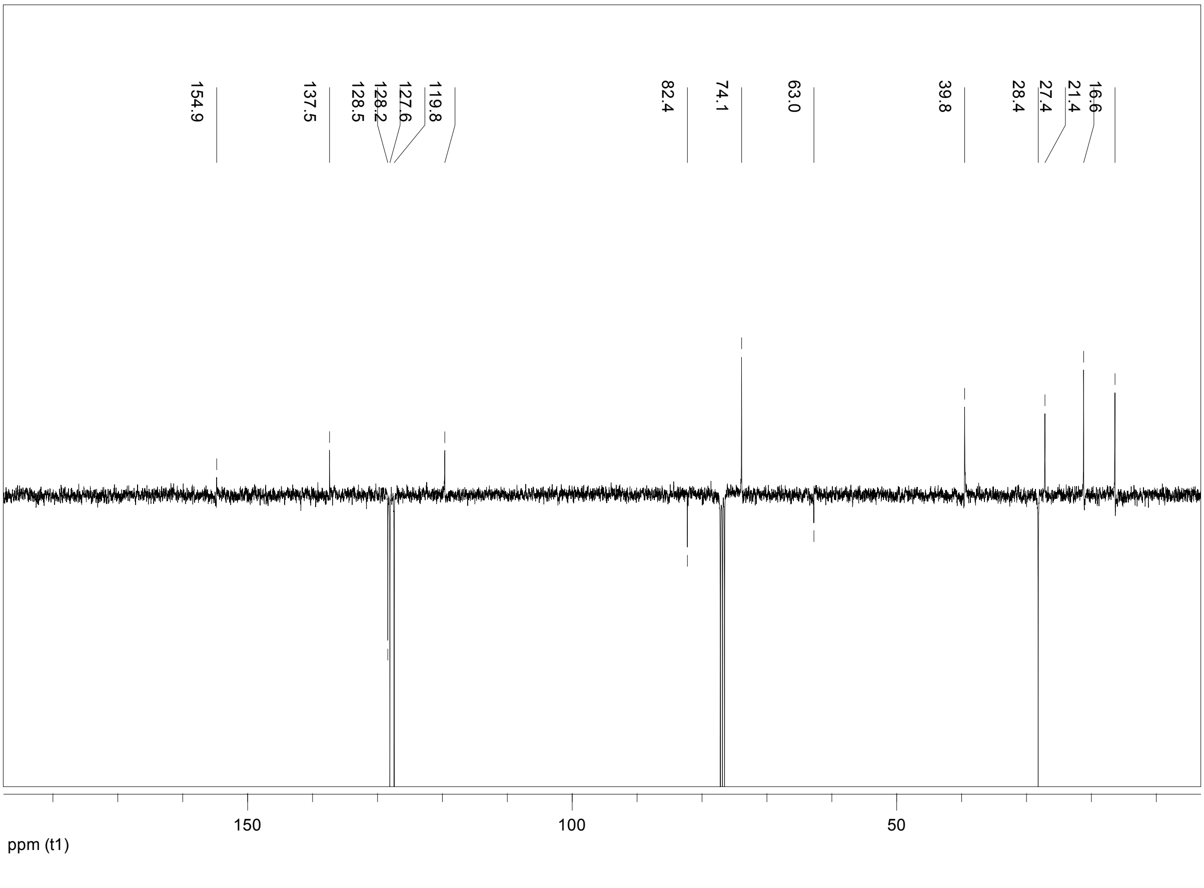

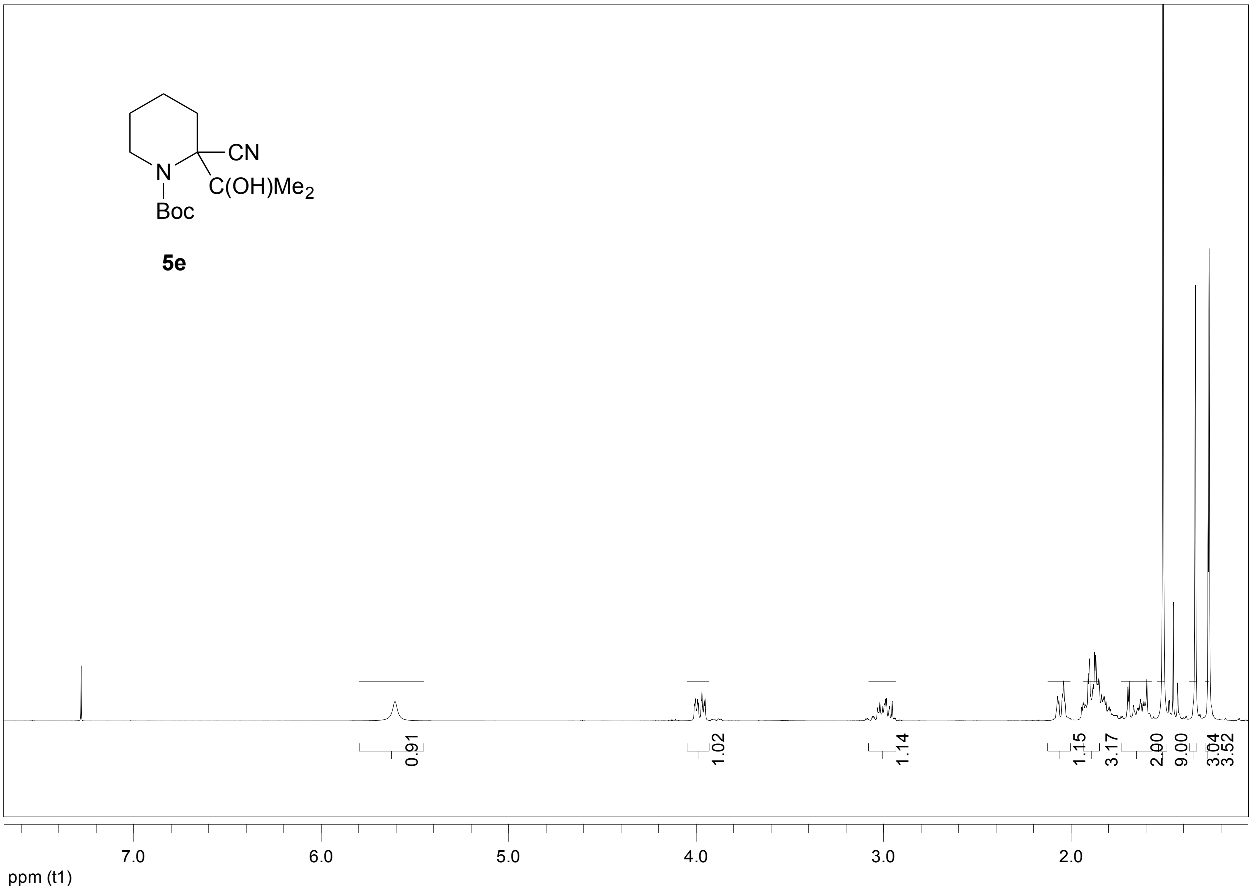
CN
N
C(OH)Me2
Boc
5e
0.91
1.02
1.14
1.15
3.17
2.00
9.00
3.04
3.52
7.0
6.0
5.0
4.0
3.0
2.0
ppm (t1)

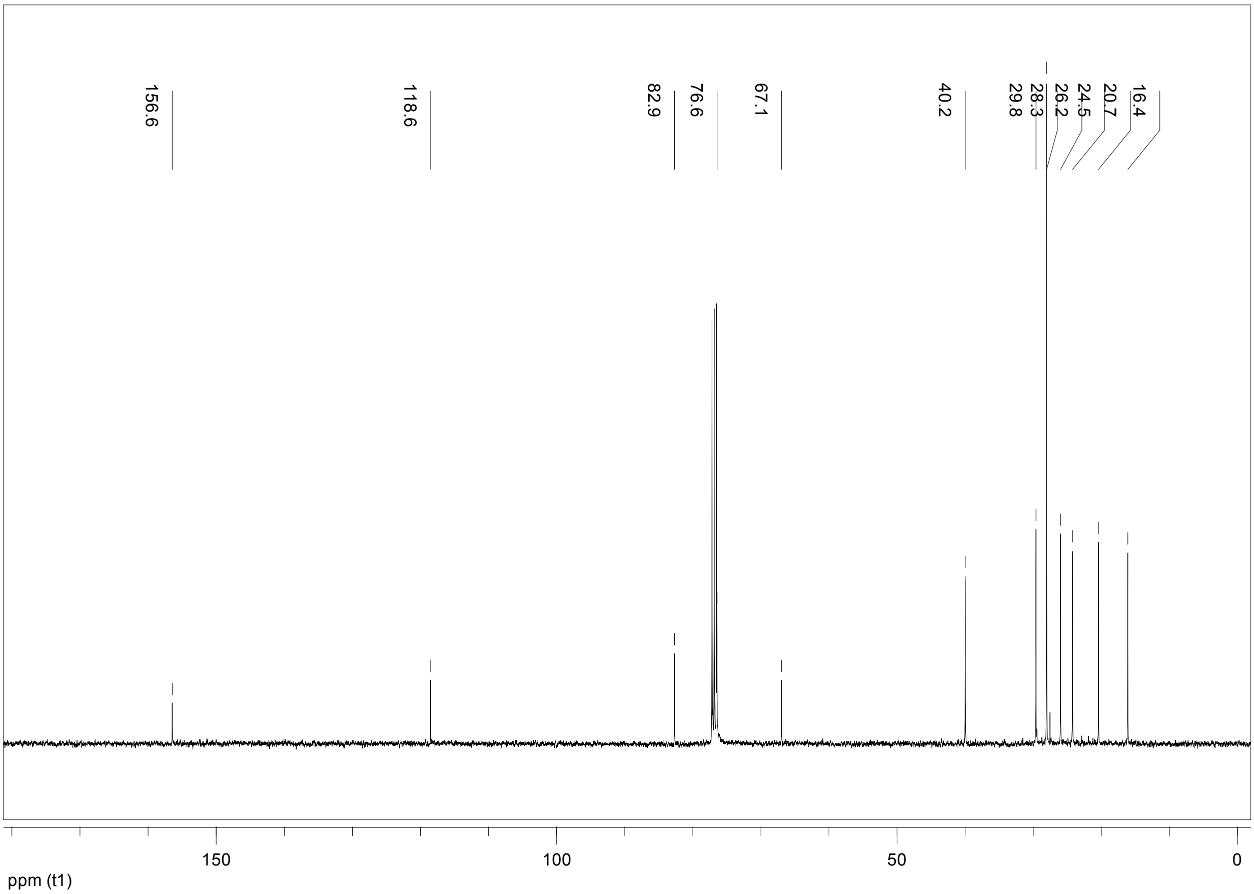
156.6
118.6
82.9
76.6
67.1
40.2
29.8
28.3
26.2
24.5
20.7
16.4
150
100
50
0
ppm (t1)

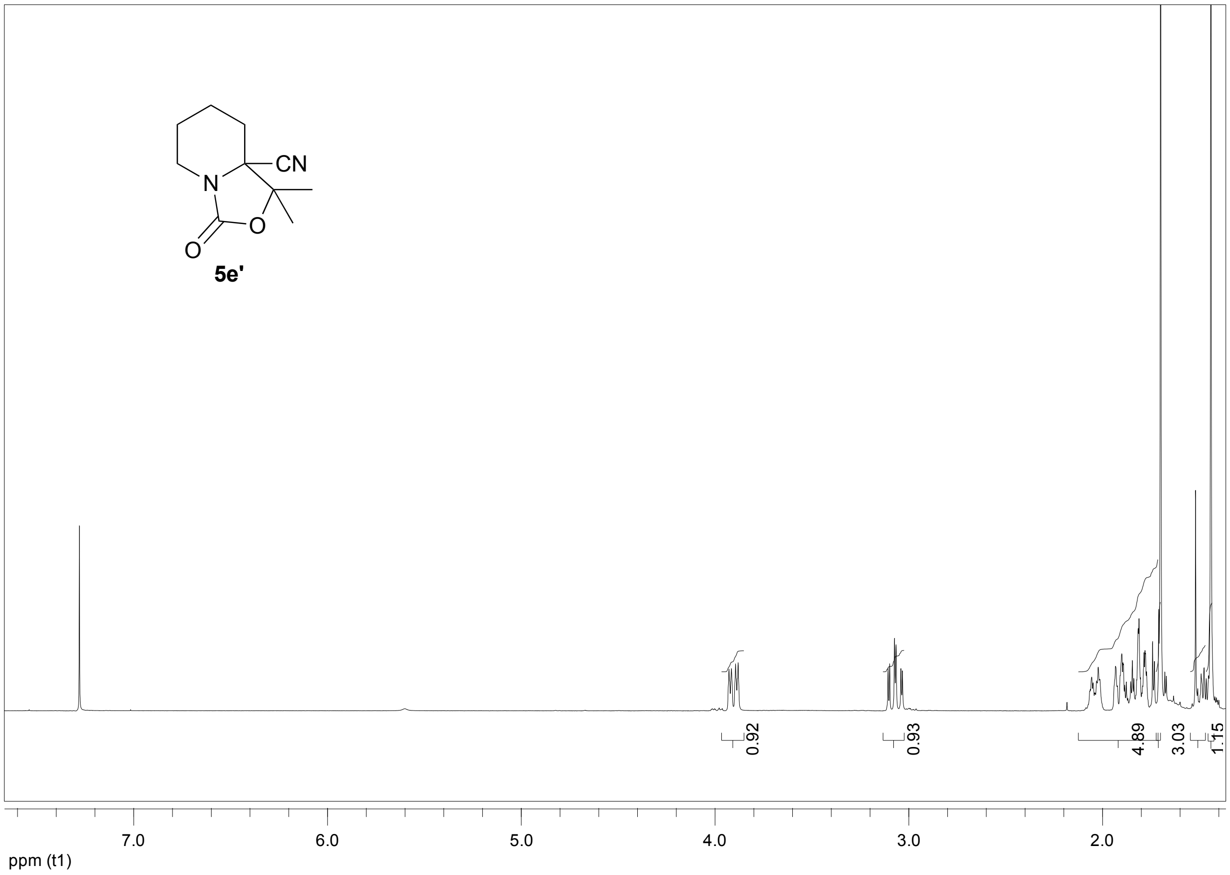
CN
N
O
O
5e'
0.92
0.93
4.89
3.03
1.15
7.0
6.0
5.0
4.0
3.0
2.0
ppm (t1)

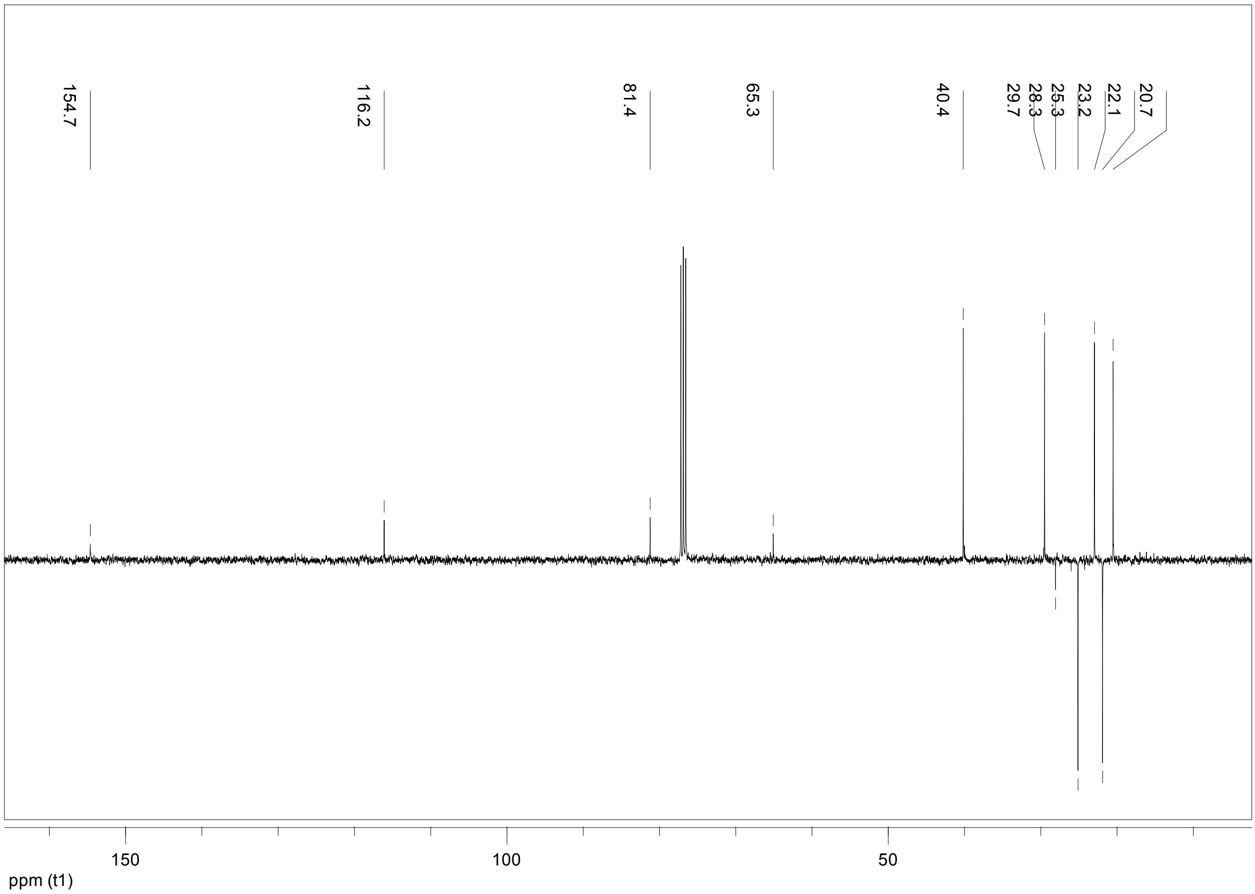
154.7
116.2
81.4
65.3
40.4
29.7
28.3
25.3
23.2
22.1
20.7
150
100
50
ppm (t1)

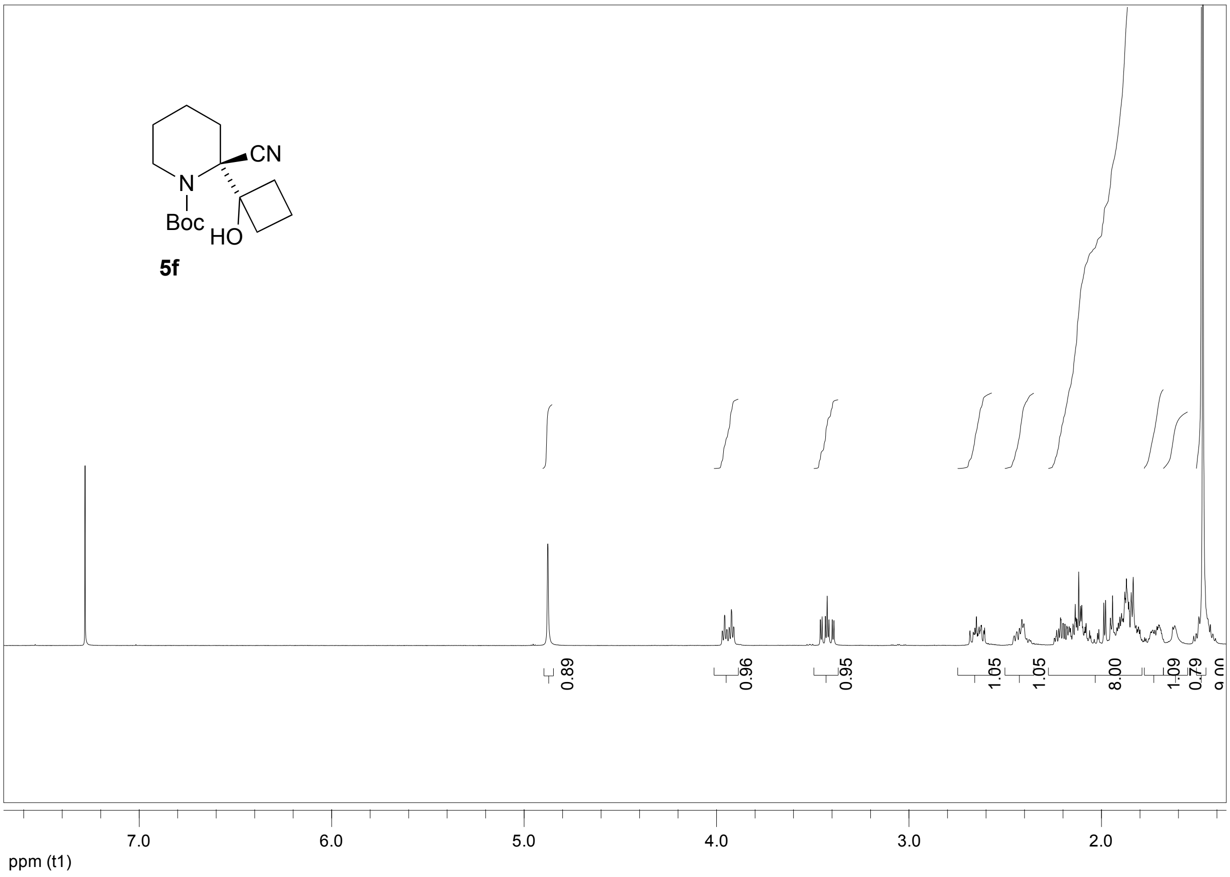
CN
N
Boc
HO
5f
0.89
0.96
0.95
1.05
1.05
8.00
7.0
6.0
5.0
4.0
3.0
2.0
ppm (t1)

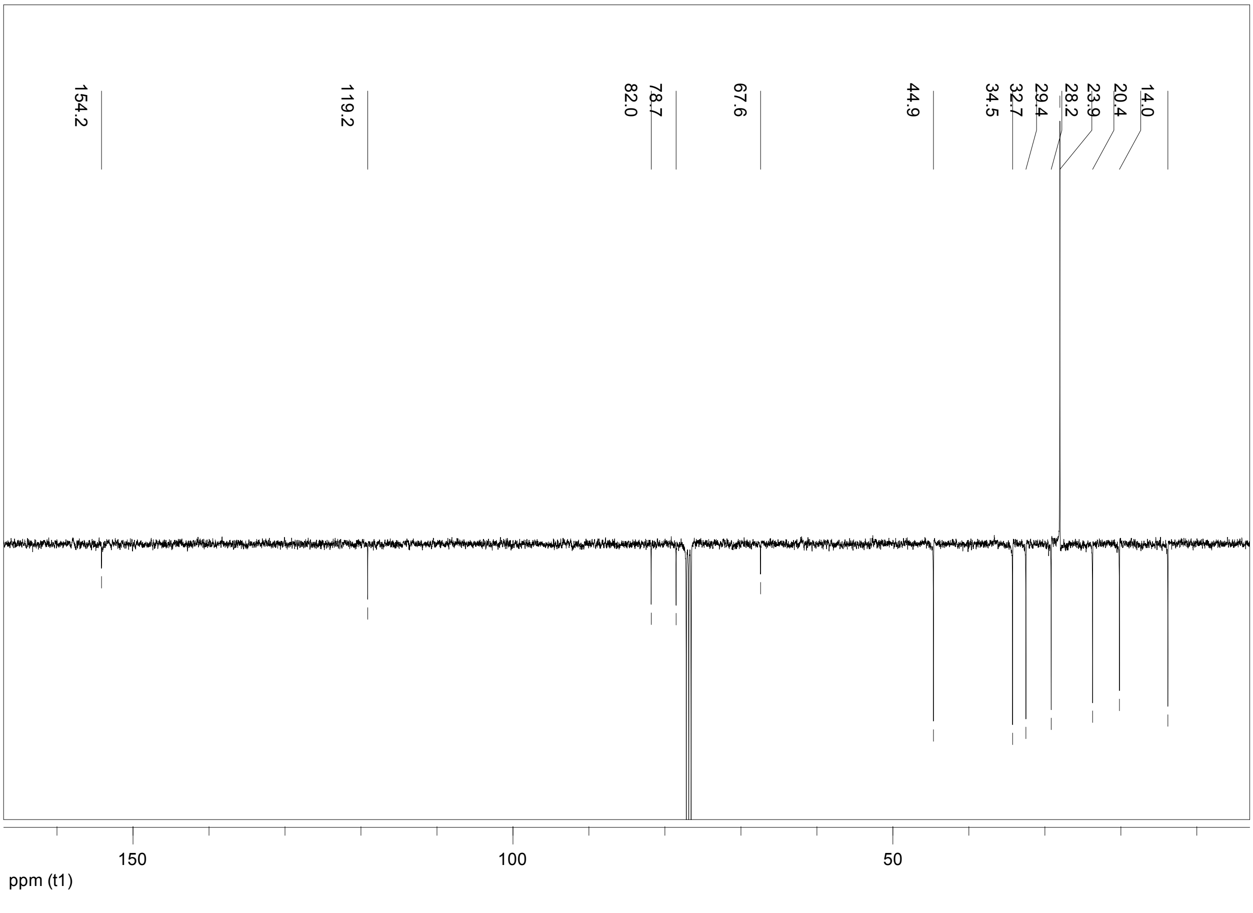
154.2
119.2
82.0
78.7
67.6
44.9
34.5
32.7
29.4
28.2
23.9
20.4
14.0
150
100
50
ppm (t1)

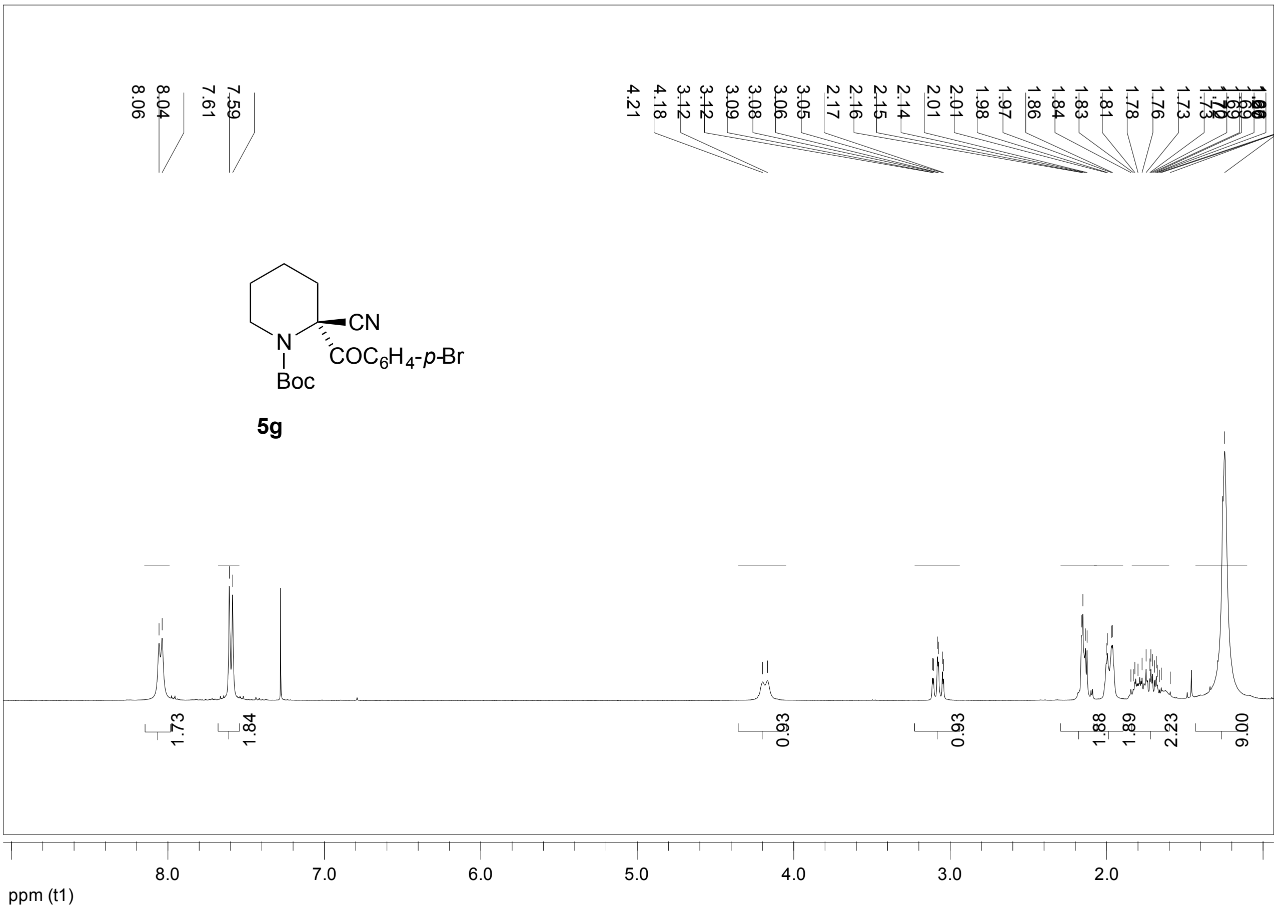
8.06 8.04 7.61 7.59
4.21 4.18 3.12 3.12 3.09 3.08 3.06 3.05 2.17 2.16 2.15 2.14 2.01 2.01 1.98 1.97 1.86 1.84 1.83 1.81 1.78 1.76 1.73
CN
N
COC6H4-p-Br
Boc
5g
1.73 1.84 0.93 0.93 1.88 1.89 2.23 9.00
8.0 7.0 6.0 5.0 4.0 3.0 2.0
ppm (t1)

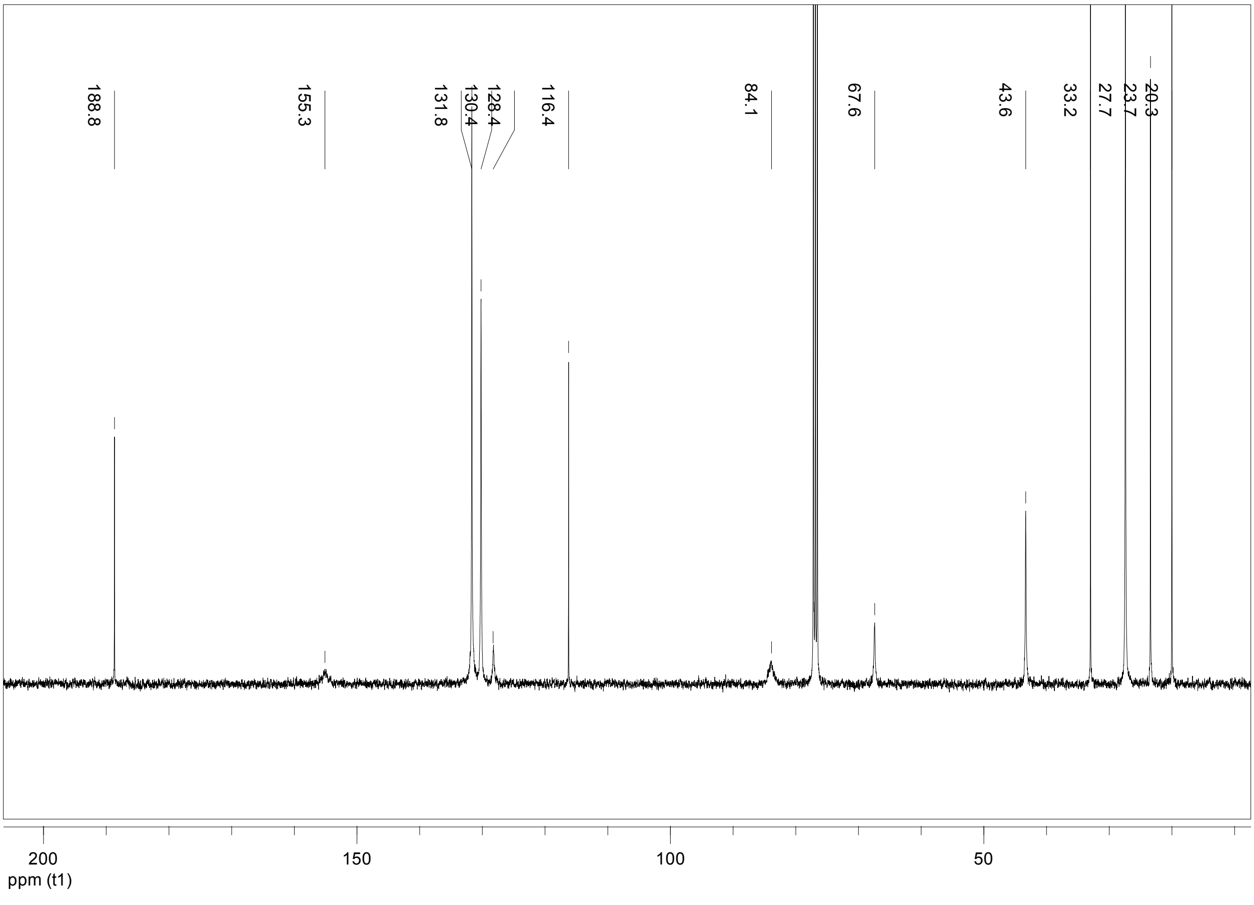
188.8 155.3 131.8 130.4 128.4 116.4 84.1 67.6 43.6 33.2 27.7 23.7 20.3
200 150 100 50
ppm (t1)
